# Supporting physical activity through co-production in people with severe mental illness (SPACES): study protocol for a pragmatic, parallel-group randomised controlled trial

**DOI:** 10.1136/bmjopen-2025-115528

**Published:** 2026-10-08

**Authors:** Trys Burke, Philip Hodgson, Gareth Jones, Rebecca J Beeken, Eleanor Bowes, Cindy Cooper, Robert Copeland, Sam Dawson, Matthew Faires, Zoe Guy, Andrew Hill, Emily Hillison, Michelle Horspool, Jinshuo Li, Katarzyna Karolina Machaczek, Freya Marks, Steve Parrott, Helen Quirk, Ines Rombach, Brendon Stubbs, Garry Tew, Gemma Traviss-Turner, Emily Turton, Stephen J Walters, Scott Weich, Ellie Wildbore, Emily Jane Peckham

**Affiliations:** 1 School of Health Sciences, Bangor University, Bangor, UK; 2 Institute for Health and Care Improvement, York St John University, York, UK; 3 Tees, Esk and Wear Valleys NHS Foundation Trust, Darlington, UK; 4 Advanced Wellbeing Research Centre, Sheffield Hallam University, Sheffield, UK; 5 Leeds Institute of Health Sciences, University of Leeds, Leeds, UK; 6 School of Medicine and Population Health, The University of Sheffield, Sheffield, UK; 7 PPIE Representative, Sheffield Partnership University NHS Foundation Trust, Sheffield, UK; 8 Sheffield Centre for Health and Related Research, The University of Sheffield, Sheffield, UK; 9 Department of Health Sciences, University of York, York, UK; 10 Centre for Applied and Social Care Research, Sheffield Hallam University, Sheffield, UK; 11 Institute of Psychiatry, Psychology and Neuroscience, King’s College London, London, UK

**Keywords:** Exercise, Schizophrenia & psychotic disorders, Peer Group, MENTAL HEALTH, Randomized Controlled Trial, Physical Therapy Modalities

## Abstract

**Introduction:**

Life expectancy is reduced by around 15–20 years for people with severe mental illness (SMI) compared with those without this condition. Many early deaths stem from preventable physical health problems linked to behaviours such as poor diet, smoking and low physical activity. Increasing physical activity can improve overall health. People with SMI are typically less active than the general population and supporting them to increase activity could help reduce the life expectancy gap. This study aims to explore the clinical and cost-effectiveness of a co-produced physical activity programme tailored to people with SMI (the SPACES intervention).

**Methods and analysis:**

This two-arm, multi-site, pragmatic, parallel-group randomised controlled superiority trial will assess the clinical and cost-effectiveness of a co-produced physical activity intervention across National Health Service (NHS) mental health trusts in England and health boards in Wales. A total of 550 participants (275 per arm) will be randomised 1:1 into the intervention or usual care arms. The usual care arm will receive treatment as usual, while the intervention arm will receive treatment as usual plus an 18-week physical activity programme comprising walking sessions, indoor activities and community taster sessions. The intervention group will also receive six one-to-one sessions: four long-form consultations (entry, midpoint and one either side of the exit) and two shorter check-ins (between the entry and midpoint and between midpoint and the first exit consultation). The primary outcome is objective accelerometer-derived minutes per day of moderate to vigorous physical activity at 6 months post randomisation. Secondary outcomes are light physical activity, sedentary behaviour, self-reported physical activity and sedentary behaviour, body mass index, depression, anxiety, loneliness, health-related quality of life, healthcare resource use, predictive behaviour and adverse events. Measures will be taken at baseline and at 3, 6 and 12 months post randomisation.

**Ethics and dissemination:**

This trial was provided ethical approval by the West of Scotland NHS Research Ethics Committee and the Health Research Authority on 14 May 2024 (ref: 24/WS0040). All participants will provide written informed consent before participation. Findings from this study will be shared publicly and disseminated through publication in peer-reviewed journals, conference presentations and social media outlets.

**Trial registration number:**

ISRCTN23019026.

**STRENGTHS AND LIMITATIONS OF THIS STUDY**

- Codesigned study addressing physical activity to reduce health disparities in people with severe mental illnesses.
- Builds on prior research by exploring feasibility and acceptability within National Health Service (NHS) care pathways.
- It is unclear if the primary outcome—a 6-minute daily increase in moderate to vigorous physical activity—will yield meaningful mental health or quality-of-life gains (assessed as secondary outcomes).
- Variation across NHS trusts may introduce site-level confounding.
- Unavoidable unblinded follow-up assessments may bias self-reported measures, though the primary outcome is objective.

## Introduction

 Individuals living with severe mental illness (SMI), such as schizophrenia, bipolar disorder and schizoaffective disorder, die on average of 15–20 years earlier than the general population.^1^ More than 70% of these premature deaths are linked to preventable physical health conditions, including cardiovascular disease and diabetes.^2^ Despite rising life expectancy in the general population, it has declined for people with SMI^3^, highlighting a growing health inequality that the National Health Service (NHS) Long Term Plan aims to address.^4^

Sedentary behaviour (waking activities with low energy expenditure; eg, sitting or reclining) and physical inactivity (insufficient movement to meet activity guidelines) are among the leading causes of preventable mortality and morbidity in the UK.^5^ The WHO, Public Health England and Public Health Wales recognise tackling these behaviours as equally important as promoting smoking cessation.^4 6 7^ However, research indicates that people with SMI engage in significantly less physical activity (PA) and more sedentary behaviour than the general population.^3 8^ Barriers to PA for individuals with SMI include increased mental health symptoms, lack of social support and medication side effects.^9^

Traditional treatment approaches for SMI predominantly focus on pharmacotherapy and/or psychotherapy and often fail to improve physical health or quality of life (QoL).^10^ Obesity and inactivity remain major contributors to poor physical health, and behavioural interventions could help offset the weight gain caused by psychotropic medications.

In the wider population, higher PA levels and reduced sedentary behaviour are linked to lower risks of cardiovascular disease, diabetes and metabolic syndrome.^5^ While small-scale studies suggest that PA may improve mental health symptoms in people with schizophrenia and major depression,^11^ evidence on the effectiveness, acceptability and feasibility of specific interventions for SMI is still limited. A recent systematic review found that existing interventions fail to engage people with SMI effectively, highlighting the need for tailored, accessible programmes.^8^ Currently, there is little consistency in how PA interventions are offered to people with SMI in NHS settings.^12^ Our work with service users confirms that current programmes are difficult for individuals with SMI to access.

Prior to this study, a feasibility study of our co-produced intervention was conducted.^13^ The feasibility study investigated the acceptability and practicality of the co-produced PA intervention for individuals with SMI across six NHS mental health trusts in England. The study’s findings informed the design of the randomised controlled trial (RCT) reported in this protocol paper. The integration of PA into community-based healthcare aligns with recovery frameworks such as the Connectedness, Hope & Optimism, Identity, Meaning, and Empowerment^14^ framework that emphasise the importance of connectedness, hope, identity, meaning and empowerment for a person’s recovery. This is distinct from interventions delivered outside the healthcare system. Therefore, bespoke PA programmes designed to enhance QoL, mental health and physical health are urgently needed for people with SMI.

Our feasibility study demonstrated that the co-produced and tailored SPACES intervention is both deliverable and acceptable to people with SMI.^15^ The next step is to evaluate the clinical and cost-effectiveness through a full-scale RCT.

### Objectives

The main objective of this study is to establish the clinical effectiveness of the SPACES intervention for people with SMI compared with usual care, measured by minutes of moderate to vigorous physical activity (MVPA) per day. The secondary objective is to establish the cost-effectiveness of the intervention for people with SMI compared with usual care.

## Methods and analysis

### Patient and public involvement

Development of an acceptable and feasible complex intervention requires co-production with service users and clinicians as well as researchers. Patient and public involvement and engagement (PPIE) is essential for this study. The PPIE group is led by PPIE leads. PPIE has been and will continue to be integrated into all stages of the study, and we have public contributors on the Programme Steering Committee (PSC). The PPIE group aims to consist of up to eight members, including a mix of age, gender, SMI type, illness experience and varying engagement in PA. The PPIE group meets as frequently as required for the SPACES study (roughly every 6 months) to discuss various components of the trial including, but not limited to, design, development and training for intervention, decisions about outcome measures, recruitment, topic guides, patient information sheets, analysis and dissemination. The PPIE group participants are not eligible to be a participant for the SPACES trial due to their involvement in the PPIE group.

### Trial design

This study is a two-arm, multi-site, pragmatic, parallel-group randomised controlled superiority trial. Eligible participants will be randomised into one of two study arms: usual care plus SPACES intervention aimed at increasing PA or usual care alone. Outcome measures will be collected at baseline and at 3, 6 and 12 months. See online supplemental file 1 for a structured summary of the SPACES RCT.

### Trial setting

The study will be conducted across geographically dispersed NHS mental health trusts and primary care services in England and Wales. The intervention will be delivered at community-based venues. The characteristics of trial participants and how they will be recorded are shown in table 1.

**Table 1:** Characteristics of trial participants

Characteristic	The people we would expect to see included
Age	18 years or older
Sex	Male or female as assigned at registration of birth
Gender	Man, woman, prefer not to say, prefer to self-describe as…
Race, ethnicity and ancestry	White English/Welsh/Scottish/Northern Irish/British Irish Gypsy or Irish Traveller Roma Any other white background (please specify) Black/African/Caribbean/black British African background (please specify) Caribbean Any other black/black British or Caribbean background (please specify) Asian/Asian British Indian Pakistani Bangladeshi Chinese Any other Asian background (please specify) Mixed/multiple ethnic groups White and black Caribbean White and black African White and Asian Any other mixed/multiple ethnic background (please specify) Other ethnic group Arab Any other ethnic group (please specify) Prefer not to say
Socioeconomic status	Based on earnings: £0–300 per week (equals to about £0–1290 per month or £0–15 490 per year) £0–300 per week (equals to about £0–1290 per month or £0–15 490 per year) £491–740 per week (equals to about £2111–3230 per month or £25 341–38 740 per year) £741–1111 per week (equals to about £3231–4830 per month or £38 741–57 930 per year) £1112 or more per week (equals to about £4831 or more per month or £57 931 or more per year)
Geographical location	The study will be conducted across geographically dispersed NHS mental health trusts and primary care services in England and Wales.
Other characteristics relevant to the trial	ICD-10 or DSM-V diagnosis of SMI as documented in GP or psychiatric notes (schizophrenia (F20 and subcodes); schizoaffective disorder (F25 and subcodes); other psychotic and delusional disorders (not of organic origin) (F21–F24 and F26–F29 and subcodes); bipolar and manic episodes (F30 and F31 and subcodes)).

DSM-V, Diagnostic and Statistical Manual of Mental Disorders, Fifth Edition; GP, general practitioner; ICD-10, International Classification of Diseases, Tenth Revision; NHS, National Health Service; SMI, severe mental illness.

### Inclusion and exclusion criteria

#### Inclusion criteria

1. Aged 18 years or more.
2. International Classification of Diseases, Tenth Revision or Diagnostic and Statistical Manual of Mental Disorders, Fifth Edition diagnosis of SMI as documented in general practitioner (GP) or psychiatric notes (schizophrenia (F20 and subcodes); schizoaffective disorder (F25 and subcodes); other psychotic and delusional disorders (not of organic origin) (F21–F24 and F26–F29 and subcodes); bipolar and manic episodes (F30 and F31 and subcodes)).
3. Able to walk unaided.
4. Willing to wear an accelerometer.

#### Exclusion criteria

Patients will not be eligible for the study if they meet one or more of the exclusion criteria:

1. People who lack capacity to participate in the trial as guided by the Mental Capacity Act 2005.^16^
2. Primary diagnosis of drug or alcohol abuse.
3. Medical contraindication of PA, ascertained by the GP or mental health team.
4. Already physically active (eg, >300 min/week of self-reported MVPA).
5. Non-English speakers.
6. People who took part in the feasibility study.

### Consent

Participants will be invited to take part in the study by their GP or mental health professional. If interested, they will be referred to a SPACES researcher who will discuss involvement in the trial with them. If interested in taking part, informed consent will be collected face to face with both the participant and the SPACES researcher signing and dating the consent form (see online supplemental file 2). Copies of informed consent will be sent to both the participant and their GP/health professional.

### Intervention and comparator description

#### Usual care

Usual care is defined as ‘the wide range of care that is provided in a community whether it is adequate or not, without a normative judgement’.^17^ Including a usual care group enables the study to identify any differences in outcomes for people with SMI based on whether they received the intervention or not. Participants in both arms will receive their usual NHS care as per local protocols. Acknowledging that usual care may differ widely between sites, data on what constitutes usual care have been collected across recruiting institutions. Care coordinators of participants in both arms can signpost to local services on a participant’s request.

#### SPACES intervention

The SPACES intervention is designed to support individuals in starting and sustaining increased PA while reducing sedentary behaviour. It consists of two key components: weekly group-based sessions and one-to-one sessions with a trained physical activity coordinator (PAC). PACs can come from a range of backgrounds. During the feasibility study, these included occupational therapists, PA therapists, PA instructors, mental health workers, care workers, peer mentors and from within the sites’ own research team. PAC training comprises 2 days and introduces the intervention and the role and responsibilities of a PAC, including General Data Protection Regulation (GDPR) issues, withdrawals and adverse and serious adverse events. Day 1 also includes training on core knowledge and skills for delivery to people with SMI. Day 2 provides a full run through each component of the intervention and involves several practical tasks for PACs to complete in small groups.

Participants assigned to the intervention arm will be offered up to 18 weekly group-based sessions and six one-to-one sessions (including four ‘long-form’ PA consultations—entry, midpoint and either one side of the exit—plus two shorter check-ins—between the entry and midpoint, and between midpoint and the first exit consultation). All one-to-one sessions will be delivered by a trained PAC. The weekly group-based sessions will last up to 2 hours and include three parts: up to 60 min of PA, up to 30-minute themed discussion and 30 min of social time. The PA portion will rotate between outdoor walking, indoor activity classes and community taster sessions. Weeks 1 and 18 will involve gentle PA and a greater focus on the social component. These 2 weeks will not include a themed discussion (figure 1).

**Figure 1:**
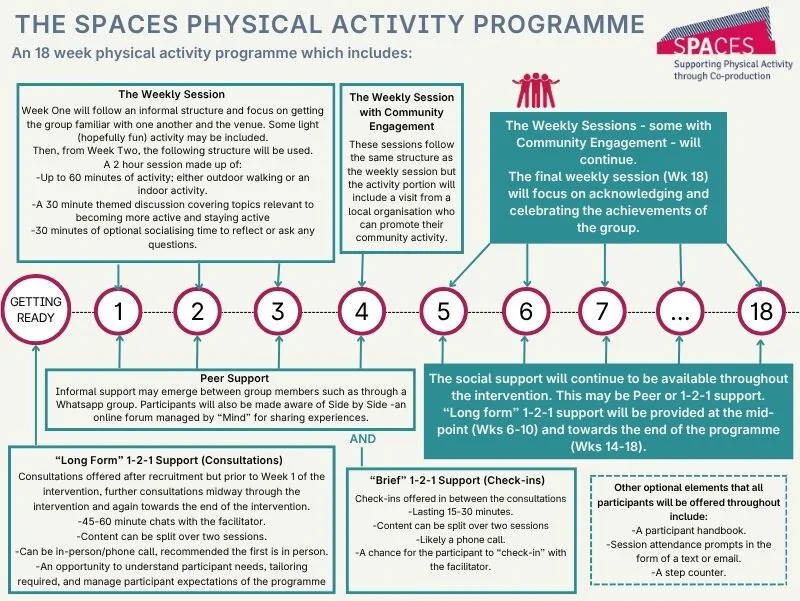
SPACES intervention infographic.

During weeks 2–17, it is recommended that PACs include six outdoor sessions, six indoor sessions and four community taster sessions. Themed discussions will also take place during weeks 2–17 and focus on sharing practical information and strategies related to PA, covering topics such as getting started with PA, maintaining motivation, the benefits of reducing sedentary time, overcoming barriers and discovering local indoor and outdoor activities. The final segment will allow time for informal social interaction. Although participants will be encouraged to take part in all three components, they are free to opt out of any element. The group sessions will take place in community venues and will run weekly for 18 weeks. Two additional weeks follow at the end to support participants, post intervention.

In addition to the group sessions, participants will be offered one-to-one sessions with a PAC. These will begin with a ‘long-form’ consultation before the first group session to assess the participant’s current activity levels, set goals and address any specific needs or concerns. Additional one-to-one long-form consultations will be offered midway through the intervention, towards the end and post intervention. These will be used to review progress, discuss any challenges and reset goals. Participants will also have the option of up to four extra short-form ‘check-in’ sessions, scheduled between the long-form consultations if required. All one-to-one sessions can be conducted face to face, by phone or via video call, depending on the participant’s preference. Video calls will use approved platforms (eg, NHS ‘Attend Anywhere’), while face-to-face sessions will take place either at the participant’s home or in an NHS or community setting.

Participants will have the option to self-monitor their activity using a wearable device (eg, step counter), which will be supplied by the study team if not already owned. A participant handbook will also be supplied, offering further opportunities for self-monitoring via an activity diary. The handbook also contains key study information, NHS staff contact details, PA guidelines from the chief medical officer^18^ and practical tasks to help participants stay engaged and motivated throughout the intervention.

Participants could opt to receive attendance prompts (eg, brief telephone/text reminders) for group sessions and one-to-one consultations. Social support will be provided through peer interactions in the group sessions and professional support from the PACs. Participants in the intervention group will continue to receive their usual care or treatment alongside the PA programme, with no treatment being withheld.

The intervention was developed using a range of inputs, including existing literature,^19^ expertise in intervention design, focus and consensus groups and psychological theory (eg, self-determination theory^20^ and the Behaviour Change Technique Taxonomy^21^). The intervention development process and full details of the intervention are described elsewhere. Additionally, evidence from a PA questionnaire for individuals with SMI^22^ informed the process. The intervention was co-produced^23^ with input from people with SMI, professionals who support them and academic experts. People with lived experience of SMI, carers and physical and mental health clinicians took part in (1) focus groups to identify barriers and enablers and determine the key elements of the intervention and (2) a separate consensus group that advised on each stage of intervention development (see online supplemental file 3 for an infographic depicting the intervention development). The final intervention incorporated 14 behaviour change themes encompassing 32 individual behaviour change techniques^24^ (see table 2).

**Table 2:** BCTs identified and implemented in the SPACES intervention https://theoryandtechniquetool.humanbehaviourchange.org/tool

No	BCT theme	Specific BCT
1	1. Goals and planning	1.1 Goal setting (behaviour) 1.2 Problem solving 1.3 Goal setting (outcome) 1.4 Action planning 1.5 Review behavioural goal(s)
2	2. Feedback and monitoring	2.2 Feedback on behaviour 2.3 Self-monitoring of behaviour
3	3. Social support	3.1 Social support (unspecified) 3.2 Social support practical 3.3 Social support (emotional)
4	4. Shaping knowledge	4.1 Instruction on how to perform behaviour
5	5. Natural consequences	5.1 Information about health consequences 5.2 Salience of consequences 5.3 Information about social and environmental consequences 5.6 Information about emotional consequences
6	6. Comparison of behaviour	6.1 Demonstration of the behaviour 6.2 Social comparison
7	7. Associations	7.1 Prompts/cues
8	8. Repetition and substitution	8.1 Behavioural practice/rehearsal 8.7 Graded tasks
9	9. Comparison of outcomes	9.1 Credible source 9.2 Pros and cons
10	10. Reward and threat	10.4 Social reward
11	11. Regulation	11.2 Reduce negative emotions
12	12. Antecedents	12.1 Restructuring the physical environment 12.2 Restructuring the social environment 12.5 Adding objects to the environment
13	13. Identity	13.2 Framing/reframing
14	15 Self-belief	15.1 Verbal persuasion about capability 15.3 Focus on past success 15.4 Self-talk

BCT, behaviour change technique.

There are no specific criteria for discontinuing or modifying allocated interventions; however, there will be some flexibility within the PA intervention as PACs will use rating of perceived exertion scales^25^ to enable participants to exercise at an appropriate intensity for themselves. To facilitate this further, within the PAC manual, PACs will be provided with possible modifications to the indoor exercises to increase or decrease exercise intensity. Participants who choose to withdraw from the intervention or are advised by their medical practitioner to do so due to health reasons are encouraged to continue with the study follow-up.

Following lessons learnt from the feasibility trial,^15^ the plans below will be used to promote participant retention throughout the trial period.

- Regular communication with study participants will be provided throughout the study, with participants being provided with SPACES research team contact details for any potential queries. Participants randomised to the usual care arm will be contacted by SPACES researchers between follow-ups to maintain engagement with the trial during the intervention period. Participants in both arms will be contacted to provide study updates between the 6- and 12-month follow-ups.
- Participants will be advised that they are able to contact a member of the research team if they require assistance with completing a questionnaire.
- Participants will be asked to provide contact details of up to three people who can be contacted if the SPACES team is unable to contact the participant directly.

Participants will receive usual care throughout the trial. There are no prohibited factors to trial participation.

There is no post-trial care planned.

### Outcomes

Data collection will take place across four time points: baseline and 3-, 6- and 12-month follow-ups post randomisation. The following outcomes will be measured at each time point.

#### Primary outcome measure

The primary outcome measure will be 6-month post-randomisation objective accelerometer-derived minutes of MVPA per day (defined as a minimum of 16 hours/day of wear time for at least 3 days over the 10-day collection period).

#### Accelerometer details

An accelerometer is a device that tracks movement such as a person’s PA. When used to measure how active someone is, it is usually worn on the wrist and looks like a watch. It records how much and how intensely someone moves over time. These devices can also track sleep patterns and how much time a person spends being sedentary. The data collected from the accelerometer can be used to estimate time spent sitting, doing light, moderate or vigorous activity each day. This information is reported as the average time per day across all valid days the device was worn. In this study, participants will wear the Axivity AX6 accelerometer^26^ for 10 days. SPACES site researchers will use software (OMGUI for the Axivity AX6) to download the data after each 10-day period. These data will then be securely sent to the central SPACES study team and further analysed using GGIR version 2.9-0^27 28^ in R V.4.3.3; RStudio V.2023.06.0+421.^29^ A minimum of 16 hours/day of wear time for at least 3 days will be accepted as valid wear time over the 10-day collection period. Intensity gradient cut-off points of <30, 30–100, 101–400 and >401 mg (inactive, light, moderate and vigorous activities, respectively) will be used. Mean MVPA per day is calculated on 5 s epochs with 1-minute bout duration, inclusion criteria of >80% (which defines what fraction of a bout needs to be above the relevant intensity threshold).

If there are any problems with the accelerometer data, the participant will be contacted and asked if they are willing to wear the accelerometer for a further 10 days. It will be made clear that they do not have to do this and not doing it will not affect their participation in the study in any way.

#### Secondary outcomes

The following outcome measures will be assessed at baseline and 3, 6 and 12 months post randomisation:

- *PA (light PA and MVPA) and sedentary behaviour* will be collected by wrist-worn accelerometers as described above.
- *Self-reported PA and sedentary behaviour* will be measured using the Simple Physical Activity Questionnaire (SIMPAQ), a validated self-report instrument. The SIMPAQ measures MVPA, light PA, sedentary behaviour and sleep.
- *Body mass index (BMI)* will be calculated by measuring height and body weight at baseline and body weight (using portable scales) at baseline and follow-up.
- *Patient Health Questionnaire-9 (PHQ-9*)^30^ is a measure of depression severity and response to treatment using a 3-point Likert scale across nine items. PHQ-9 is measured on a scale from 0 to 27; higher scores indicate more severe depressive symptoms.
- *Generalized Anxiety Disorder-7 (GAD-7)*^31^ is a brief measure of generalised anxiety disorder and its severity using a 3-point Likert scale across seven items. GAD-7 is measured on a scale from 0 to 21; higher scores represent increasing severity of anxiety.
- *EuroQoL 5D-5L (EQ-5D-5L)*^32^ is an instrument for measuring QoL. The EQ-5D measures five domains of health, including mobility, self-care, usual activities, pain/discomfort and anxiety/depression, using 5-point Likert scales, along with a visual analogue scale (VAS) that measures overall health. Current National Institute for Health and Care Excellence guidelines^33^ recommend the use of Hernández Alava *et al*^34^ for estimating the EQ-5D-5L utility score, which is measured on a scale from –0.224 to 1.00 (full health). EQ-5D VAS is measured on a scale from 0 (worst imaginable health state) to 100 (best imaginable health state).
- *Recovering Quality of Life (ReQoL)*^35^ is a 20-item measure of QoL, split into the 10-item index score (first 10 questions) and an overall score. The score range for 10 items is 0–40; for 20 items the score range is 0–80. A higher score indicates a better QoL.
- *University of California, Los Angeles (UCLA) 3-Item Loneliness Scale*^36^ is an instrument for measuring loneliness. The scale comprises three questions that measure three dimensions of loneliness and has three response categories: hardly ever, some of the time and often. The scores range from 3 to 9, with a higher score indicating higher loneliness.
- *Healthcare resource use:* a bespoke healthcare questionnaire will be used to provide information on the participant’s use of primary, secondary and common service use of healthcare services, with special attention paid to PA and mental health-related services.
- *Predictive behaviour questionnaire:* a questionnaire designed to predict participant PA has been created, based on the theory and planned behaviour^37^ and the Basic Psychological Needs in Exercise Scale.^38^ The questionnaire was designed with lived experience coapplicants and PPIE members to ensure wording and questionnaire length were appropriate. The questionnaire comprises 26 items, each rated on a 7-point Likert scale, with higher scores indicating more favourable outcomes.

Participants are asked to complete demographic data and provide information about health behaviours (smoking and diet) at baseline only (see table 3 for more details).

**Table 3:** Schedule of enrolment, interventions, assessments and visits for participants

Assessment	Time required	Timeline (months post randomisation)			
		Baseline	3-month follow-up	6-month follow-up	12-month follow-up
Eligibility and consent					
Eligibility	NA	X			
Consent	15 min	X			
Background					
Relevant contact details	5 min	X			
Body mass index	5 min	X	X	X	X
Health behaviours (diet and smoking)		X			
Mental health details					
Mental health history		X			
Current mental health status		X	X	X	X
Current medications		X	X	X	X
Referrals to mental health services		X	X	X	X
Admissions to hospital related to mental health		X	X	X	X
Physical activity					
Accelerometer data collection Minutes of moderate to vigorous physical activity (MVPA) per day	10 days	X	X	X	X
Adverse event reporting		Ongoing collection			
Questionnaires					
Patient Health Questionnaire-9 (PHQ-9)		X	X	X	X
Generalized Anxiety Disorder-7 (GAD-7)		X	X	X	X
Simple Physical Activity Questionnaire (SIMPAQ)		X	X	X	X
Health-state utility (Euro Quality of Life 5 Dimensions (EQ-5D))		X	X	X	X
Recovering Quality of Life (ReQoL)		X	X	X	X
University of California, Los Angeles (UCLA) 3-Item Loneliness Scale		X	X	X	X
Healthcare resource use		X	X	X	X
Predictive behaviour questionnaire		X	X	X	X

### Harms

The SPACES trial is considered a low-risk research study. However, given the physical nature of the intervention, some adverse events are to be expected. For example, transient muscle soreness, falls and musculoskeletal injury are possible (although unlikely) consequences of PA.

Adverse events will be documented on an individual basis as they occur; they will be categorised as serious if they meet any of the following criteria: life threatening; results in death; requires unplanned or prolonged hospitalisation; results in persistent or significant disability or incapacity; and results in a congenital abnormality or birth defect. All serious adverse events will be reviewed within 24 hours of being reported by both the chief investigator and an independent reviewer. An ongoing summary of adverse events will be maintained and presented to the Data Monitoring and Ethics Committee (DMEC) during their scheduled meetings. This summary will include details such as the nature of the event, date of onset, severity, any actions taken, outcome, whether the event was anticipated and an assessment of its potential link to the study procedures.

All adverse event reports will be anonymised, with participants identified solely by their study ID number—no personal identifying information will be included. Examples of adverse events may include, but are not limited to:

- Muscle soreness that does not resolve within 72 hours after a PA session.
- A significant worsening of existing pain persisting beyond 72 hours post-session.
- The onset of new musculoskeletal pain lasting more than 72 hours following a PA session.
- Injuries sustained while travelling to or from a PA session.
- Skin rash, irritation or reaction relating to wearing the accelerometer during data collection.

All deaths will be recorded regardless of causality.

### Participant timeline

The time schedule of enrolment, interventions, assessments and visits for participants is depicted in table 3. Figure 2 provides a schematic of the participants’ journey throughout the study.

**Figure 2:**
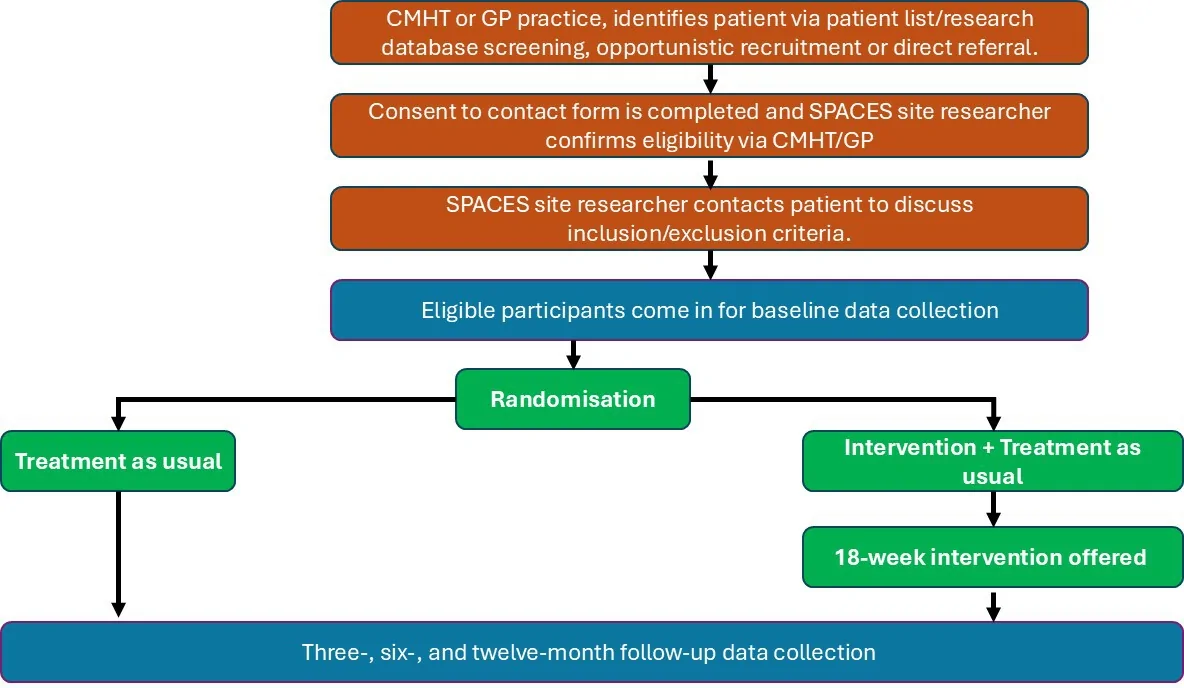
Schematic depicting the participants’ journey throughout the study. CMHT, community mental health team; GP, general practitioner.

### Sample size

The SPACES trial will randomise 550 participants (275 per arm). This sample size is based on a target difference of 6 min of MVPA per day, an SD of 28 (equivalent to a small standardised effect size of 0.21), a baseline to 6-month post-randomisation correlation of 0.60 in the primary outcome measure, 80% power, a 5% two-sided significance and 20% attrition. The target difference is derived from the Ekelund systematic review and harmonised meta-analysis^39^ on the dose–response associations between accelerometry-measured PA and sedentary time and all-cause mortality. It was estimated that the HR for all-cause mortality was a 0.70 (0.62–0.79) reduction for a 6-minute increase in minutes of MVPA per day compared with 0 (min/day). A 30% relative risk reduction in mortality for a 6-minute increase in minutes of MVPA per day is likely to be clinically and practically important.

The SD is based on estimates from the feasibility trial, in which an SD of 28.2 (95% CI 23.1 to 36.2) was observed at 6 months (n=42). The expected amount of attrition is appropriate based on data availability observed in the feasibility trial and additional plans to minimise missing data. The feasibility study did not indicate evidence of the relevant amount of intracluster correlation, which is why this was not allowed in the sample size estimation.

The recruitment and randomisation of participants to the trial were slower than anticipated. Following a review of recruitment and after discussions with the funder (National Institute for Health and Care Research (NIHR)), it was agreed to revise the target sample size downward by reducing the power to 80% (compared with 90%), with all other parameters in the sample size calculation being unchanged. The revised target sample size is 550 participants (275 per arm). The revised sample size and lower power of the study have been approved by the independent members of the Trial Steering Committee and Data Monitoring and Ethics Committee (DMEC), as well as by the funder and sponsor. The sample size amendment was made without access to comparative outcome data by randomised group. The study protocol has been amended in light of this, and this amendment to the protocol has been approved by the West of Scotland NHS Research Ethics Committee (REC) who originally approved the study and by the Health Research Authority (HRA).

### Recruitment

#### Recruitment of NHS sites

Sites will be recruited via two routes: (1) an initial email invitation from the SPACES study team, followed by a face-to-face visit, or (2) study details will be available on the NIHR portfolio, and potential sites can contact the SPACES team for further details.

#### Recruitment of participants

Participants will be identified by researchers via secondary care through clinical teams or via primary care practices. The recruitment sample aims to reflect a mix of age, gender, ethnicity, SMI type, illness experience and socioeconomic status.

Secondary care sites will use four methods to recruit participants for the SPACES trial: research database screening, direct referral, patient list screening and advertisements:

- *Research database screening:* NHS research databases will be screened to identify eligible individuals who have previously agreed to be contacted. NHS staff will contact those eligible via phone, text or letter. If interested, a meeting will be arranged to explain the study in more detail and obtain written consent if the person wishes to take part.
- *Direct referral:* mental health professionals will be asked to explain the study to eligible patients during appointments. Those who are interested in taking part will be invited to meet with a researcher where the study will be explained in more detail.
- *Patient list screening:* researchers will work with clinicians to screen patient lists for eligible participants. Those selected will receive a patient information pack, including an invitation letter and consent to contact form. Those interested in taking part will be invited to meet with a researcher where the study will be explained in more detail.
- *Advertisements:* posters and flyers will be displayed in sites that are recruiting to SPACES and on social media. Interested patients can contact their mental health professional for more information. If eligible, they will receive an information pack and, if they are still interested in participating, a meeting will be arranged with a researcher.

GP surgeries will use two recruitment methods for the SPACES trial: SMI list screening and opportunistic recruitment:

- *SMI list screening:* GP surgeries will screen their SMI Quality and Outcomes Framework registers for eligible participants. Those eligible to take part will be sent an information pack as per community mental health team list screening.
- *Opportunistic recruitment:* during health screens or other routine appointments, GPs will inform eligible patients about the study and provide them with a patient information pack. Recruitment will proceed as described above in secondary care direct referral.

### Randomisation and blinding

The randomisation schedule will be created by the study statistician at the Sheffield Clinical Trials Research Unit (CTRU) before the study begins using their own secure web-based randomisation and minimisation system.

Allocation (1:1) will be implemented using block randomisation with randomly permuted blocks of varying sizes (integer multiples of 2). Stratification by site and diagnosis of SMI will be used, namely schizophrenia (F20 and subcodes) versus schizoaffective disorder (F25 and subcodes) versus other psychotic and delusional disorders (not of known organic origin) (F21–F24 and F26–F29 and subcodes) versus bipolar disorder and manic episodes (F30–F31 and subcodes).

Allocation concealment will be maintained by the secure web-based system, which will not reveal the randomisation code until the participant is officially enrolled in the trial, after all baseline measurements are completed. The sequence is generated by the study statistician at the Sheffield CTRU before the study begins using their own secure web-based CTRU (SCRAM) randomisation system. This statistician does not participate in the randomisation process, and the details of block sizes will not be shared with the study team.

When the second baseline data are collected (ie, when the accelerometer is returned after 10 days), the SPACES researcher will contact the central team to obtain the participant’s randomisation assignment. Participants will then be informed by the researcher at the second baseline interview of their group allocation.

Due to the nature of the interventions in this study, blinding of participants, clinicians and researchers is not feasible. The trial statistician will be blind to group allocation until the statistical analysis plan is signed off. The senior statistician will be unblinded to be able to contribute to DMEC meetings.

There are no circumstances under which unblinding these personnel is permissible, and thus a procedure for revealing a participant’s allocated interventions during the trial to them is not necessary. Since clinicians and researchers are not blinded to group allocation, there is no need for an unblinding procedure.

### Data collection and management

Data will be collected at four time points: baseline and at 3, 6 and 12 months after randomisation. Demographic and medical history information will be gathered at baseline only. At each time point, including baseline, a researcher will collect primary and secondary outcome data during a face-to-face meeting. If an in-person meeting is not possible, data will be collected by phone or through a postal questionnaire where feasible. Full details are provided in table 3.

The following plans will be used to promote participant retention throughout the trial period. Regular communication with study participants will be provided throughout the study, with participants being provided with SPACES research team contact details for any potential queries. Participants randomised to the usual care arm will be contacted by SPACES researchers between follow-ups to maintain engagement with the trial during the intervention period. Participants in both arms will be contacted to provide study updates between the 6- and 12-month follow-ups.

Participants will be advised that they are able to contact a member of the research team if they require assistance with completing a questionnaire. Participants will be asked to provide contact details of up to three people who can be contacted if the SPACES team is unable to contact the participant directly.

Data management will be handled by the University of Sheffield CTRU, following their standard operating procedures for all aspects of data management, including data protection and archiving. The study will use the CTRU’s in-house data management system, Prospect, for capturing and storing participant data specific to the study. Designated staff at each site will enter data from source documents into the study-specific Prospect database. Once data have been entered, electronic validation rules are applied to the data on a regular basis; discrepancies are tracked and resolved through the Prospect database.

All collection, storage, processing and disclosure of personal information will be performed in compliance with the UK GDPR and Data Protection Act 2018.^40^ All investigators and study site staff will uphold the Act’s core principles. Any communications, reports or published results will not contain any personal data that could allow identification of individual participants. The end of study will be when the last participant has completed their 12-month follow-up. We will inform the REC once this has been completed.

Participants will be informed about their right to confidentiality. All information collected during the study will be treated with the utmost confidentiality. Data will be securely stored on paper in each NHS site’s trial management file; electronic copies will be sent to and stored at the Sheffield CTRU at the University of Sheffield.

Access to Prospect and the data contained therein is controlled through usernames and encrypted passwords. Designated staff at each site will input data from source documents into the Prospect database. Once entered, electronic validation rules are regularly applied to the data, and any discrepancies are identified and resolved within the system. All processes related to the collection, storage, processing and disclosure of personal information will comply with the GDPR and the Data Protection Act 2018.^40^

### Statistical methods

As the trial is a pragmatic, parallel-group RCT design, the statistical analysis will be performed on an intention-to-treat (ITT) basis and will be reported and presented according to the 2025 Consolidated Standards of Reporting Trials (CONSORT) statement.^41^ The primary effectiveness statistical analyses will be performed on an ITT basis, whereby participants will be analysed based on their randomisation allocation, regardless of their adherence with the protocol. Every effort will be made to follow-up all participants in both arms for research assessments.

The primary, secondary and all statistical exploratory tests will be two tailed, with alpha=0.05.

Baseline demographic (eg, age and gender), clinical (BMI, minutes of MVPA per day) and health-related QoL data (PHQ-9, GAD-7, ReQoL and EQ-5D-5L scores) will be described and summarised overall and for both treatment groups. No hypothesis testing will be performed for any baseline comparisons.

The primary aim of the trial is to determine the effectiveness of SPACES intervention to increase PA compared with treatment as usual (TAU) in people with SMI. The primary outcome is the accelerometry-determined minutes of MVPA per day at 6 months post randomisation (defined as a minimum of 16 hours/day of wear time for at least 3 days over a 10-day collection period).

An estimand is a clear and explicit description of precisely what treatment effect is to be estimated in an RCT.^42^ It is made up of five connected elements: (1) the population, (2) the treatments (we want to compare), (3) the outcome or endpoint, (4) how to account for intercurrent events and (5) population-level summary measures of how the outcomes between the different treatment conditions will be compared. Table 4 provides an outline of the estimand framework applied to the SPACES RCT.

**Table 4:** The estimand framework applied to SPACES randomised controlled trial

Estimand attribute	Description
Population	Adult patients (aged 18 years or older) with severe mental illness (SMI)
Treatment(s)	SPACES intervention/treatment in addition to usual care followed by any subsequent therapy/treatment (as needed) compared with participants randomised to usual care treatment group only followed by any subsequent therapy/treatment (as needed)
Outcome (endpoint)	Mean minutes of moderate to vigorous physical activity (MVPA) per day, measured at 6 months post randomisation
Handling intercurrent events	Stopping randomised treatment for any reason—treatment policy (as part of treatment) Switching treatments—treatment policy (as part of treatment) Changing randomised treatment or not receiving randomised treatment—treatment policy (as part of treatment) Use of other medications/treatments/therapy—treatment policy (as part of treatment) Death—while alive
Summary measures	Difference in mean minutes of MVPA per day between the two randomised groups

A treatment policy strategy to handle intercurrent events means that: *‘*Regardless of any post randomisation events, the treatment effect is described from the final outcome measure in all patients. Note that this approach cannot be used for truncated events, for example, where a variable cannot be measured due to death.’^42^

The while alive policy to handle deaths as an intercurrent event implies that we are interested in the effect on outcome until death, and that we will include participants’ outcome data in the analysis until they die and not include (or impute) any outcome data thereafter.^43^

#### Research questions answered by the estimand framework

In people with SMI, what is the difference in mean minutes of MVPA per day (as measured by an accelerometer) between participants randomised to the SPACES intervention/treatment group in addition to usual care followed by any subsequent therapy/treatment (as needed) compared with participants randomised to usual care treatment group only followed by any subsequent therapy/treatment (as needed), after 6 months from randomisation or death (whichever occurs first), regardless of study treatment discontinuation?

#### Primary statistical analysis

To address this aim, the primary effectiveness analysis on the ITT sample will compare mean accelerometer-reported minutes of MVPA per day at 6 months post randomisation between the SPACES intervention and TAU groups.^44^

For the outcome of minutes of MVPA per day, we will estimate the treatment effect at each post-randomisation follow-up time point using a three-level mixed-effects linear model. Randomised group, post-randomisation time point, randomised group by time point interactions, baseline minutes of MVPA per day and diagnosis at baseline will be included as fixed covariates, with site and participant included as random effects. The repeated post-randomisation observations for each participant at 3, 6 and 12 months (level 1) will be nested within participants (level 2), who will in turn be nested within sites (level 3). The model will include a random intercept at the site level (level 3) and a random intercept at the participant level (level 2). To be included in the primary analysis model, participants must have valid baseline accelerometry outcome data and at least one valid post-randomisation assessment of outcome (minutes of MVPA per day). We shall assume an exchangeable correlation between the repeated measures and an exchangeable covariance structure between the random effects. The point estimate and 95% CI for the mean difference in minutes of MVPA per day at 6 months post randomisation between the SPACES intervention and TAU arms will be reported from the longitudinal mixed-effects linear model.

Secondary outcomes (eg, clinical data, PHQ-9, GAD-7, ReQoL and EQ-5D-5L scores) at 3, 6 and 12 months post randomisation will be analysed in a similar way with a longitudinal mixed-effects linear regression model.^44^ For each outcome, we will estimate the treatment effect at each post-randomisation follow-up time point using a three-level mixed-effects linear model. Randomised group, post-randomisation time point, randomised group by time point interactions and baseline score will be included as fixed covariates, with site and participant included as random effects. The repeated post-randomisation observations for each participant at 3, 6 and 12 months (level 1) will be nested within participants (level 2), who will in turn be nested within sites (level 3). The model will include a random intercept at the site level (level 3) and a random intercept at the participant level (level 2). We shall assume an exchangeable correlation for the covariance structure between the random effects.

We will complement the ITT analysis of the primary outcome with a complier average causal effects (CACE) analysis as a secondary analysis to estimate the effectiveness of the SPACES intervention compared with TAU in participants with SMI focusing on those with higher uptake of group and individual sessions that formed part of the SPACES intervention. This analysis will only include data at the primary follow-up time point. For participants randomised to the SPACES intervention, we will explore the two definitions of compliance below:

- CACE1: intervention group participants will be considered compliant if they attended at least one session (this can be either an individual session or a group session).
- CACE2: intervention group participants will be considered compliant if they attended at least 50% of both group and individual sessions (at least nine group sessions and three individual sessions).

Participants randomised to the control group do not have access to the SPACES intervention and will be assumed to be adherent with usual care.

#### Health economics analysis

To evaluate the relative cost-effectiveness of the intervention and comparator, the economic analysis will take an NHS and personal social service perspective.^33^ The intervention and comparator will be costed by their components recorded alongside the study. The EQ-5D-5L^32^ will be used to calculate the quality-adjusted life year (QALY).

The use of a range of PAs and healthcare services costs will be collected during the study using participants’ self-reported questionnaire, which was piloted in the feasibility study, at baseline and follow-up. Secondary sources, for example, National Cost Collection,^45^ Unit Costs of Health and Social Care,^46^ Prescription Cost Analysis^45^ or English Prescribing Dataset,^47^ will be used to value the activities and medicines, as appropriate. This will enable an estimation of the total cost for each participant as well as the average total cost for each treatment group. As the follow-up period is 12 months, no discounting will be applied.

Economic analysis will be on an ITT basis. Between-group differences will be estimated using regression models. Mean incremental costs and QALYs will be combined into an incremental cost-effectiveness ratio when both are positive. Sampling uncertainty will be assessed using bootstrap technique and presented by plotting the cost-effectiveness plane and associated cost-effectiveness acceptability curves.^48 49^ To reflect uncertainty and enable valid inferences to be made, any missing cost and outcome data will be imputed using multiple imputation. This process will provide evidence to reach a decision on the acceptability of the intervention in terms of an effective use of NHS and personal social service resources.

#### Treatment fidelity

Obtaining reliable data on the fidelity of intervention delivery is necessary to assess whether the intervention is implemented as intended. We will distinguish between intervention fidelity, defined as the extent to which an intervention is implemented as intended,^50^ and implementation fidelity, defined as ‘active and planned efforts to mainstream an innovation within organisation’*.*^51^ This understanding will be critical for the successful implementation and large-scale delivery of the intervention as part of a multi-site RCT and its subsequent rollout across all trusts in the UK.

We have applied the best practices and recommendations for enhancing treatment fidelity in health behaviour change studies, as outlined by the National Institutes of Health Behaviour Change Consortium.^52^ The following strategies were used to ensure the fidelity of SPACES: co-production of intervention design; ongoing monitoring and improvement of PAC training; ongoing monitoring and improvement of intervention delivery through monthly ‘share and learn’ cross-site PAC meetings; separate ‘share and learn’ researcher meetings; monthly check-ins with individual site staff; observations of one to two sessions by the central SPACES team; and fidelity receipt and enactment. SPACES process evaluation protocol provides full detail on how we will assess fidelity.

#### Missing data

We will analyse the data on an ITT basis, whereby all participants recruited in the study will be entered into the analysis, regardless of whether they dropped out or partially adhered to their treatment method. Subjects will be included in the primary analysis model if they have baseline data and at least one post-randomisation measurement of MVPA. Sensitivity analyses for missing primary outcome data (minutes of MVPA per day) will be performed as described in the statistical analysis plan.

#### Planned subgroup analyses

A subgroup analysis using a mixed-effects linear regression model with the primary outcome (minutes of MVPA per day) at 6 months post randomisation as the response will be carried out. We will include an interaction term in the regression model between the randomised intervention group and subgroup to directly examine the strength of evidence for the treatment difference between the treatment groups (intervention vs TAU) varying between subgroups. The estimated regression coefficient and 95% CI for the interaction term(s) will be reported from the regression model. The point estimate and CIs for the subgroups from the regression will be graphically displayed alongside each other in a forest plot. No p values will be presented.

Gender (male or female) and diagnosis (psychosis, schizoaffective disorder, bipolar disorder or other SMI) will be the only a priori defined subgroups to be considered. Subgroup analysis will be performed regardless of the statistical significance on the overall intervention effect (intervention vs TAU).

Qualitative data will be analysed using an inductive approach and reflexive thematic analysis^53^ to explore both participants and intervention delivery team experiences of the SPACES intervention. These data will be included within a process evaluation of the study and reported under a separate protocol.

There is no planned interim analysis beyond checking the recruitment rate at the end of the internal pilot phase. See online supplemental file 4 for the full statistical analysis plan for this study.

### Study status

Recruitment to the SPACES study began on 28 October 2024. The planned recruitment end date is on 28 February 2027 and the final follow-up will be completed by March 2028. The study completion date is on 31 May 2028.

### Oversight and monitoring

This group will include the chief investigator, coapplicants, programme manager, trial coordinator, statistician and study researchers. They will monitor progress, including trial delivery and recruitment. Day-to-day management of the trial will be handled by the programme manager, with support from the chief investigators and the team at Sheffield CTRU. The group will provide regular updates to ethics committees on the study’s progress, completion or if the trial ended early.

An independent PSC will provide oversight. This group will include the chief investigator, an independent chair and at least two other independent members—one of whom will have lived experience.

The committee will be made up of independent experts, including a statistician and mental health professionals, who are not involved with the principal investigator or the study team. Its role is to oversee the trial data, focusing on quality control and data integrity and ensuring the study is being conducted according to the trial protocol. The committee will also be responsible for safeguarding the dignity, rights, safety and well-being of all participants throughout the study. Regular data reports, including any adverse events, will be provided to the committee, along with access to relevant summary data and documentation. The chair of the DMEC will be notified of any adverse events that occur during the study and will have the authority to recommend pausing or stopping the trial if the event is deemed serious. The DMEC, the research team and the statisticians will report to the PSC as needed.

To oversee the conduct of the study, the independent PSC will meet every 6 months, either in person or online, to review the trial’s progress. In addition, the Programme Management Team will hold monthly meetings with sites to monitor progress and address any arising issues.

### Protocol amendments

Any protocol amendments that affect study procedures or participant risk will be submitted for approval to the West of Scotland NHS REC. Where relevant, changes will be incorporated into an updated participant information sheet and consent form, which will be reviewed and re-signed by all current participants. All protocol modifications will also be reported to the NIHR Programme Grants for Applied Research (PGfAR) through regular progress updates.

## Ethics and dissemination

This trial was provided ethical approval by the West of Scotland NHS REC and the HRA on 14 May 2024 (ref: 24/WS0040). Approval for the revised recruitment target was provided on 11 May 2026 (ref: 24/WS0040). Any protocol amendments will be signed off by the REC and HRA and will be reported to PGfAR in progress reports. Regarding recruitment for this trial, all participants will provide written informed consent to participate. Participants will be informed they have the right to withdraw from the study at any time and for any reason, and all participants are made aware that withdrawal will not affect their usual care (see online supplemental file 2—participant consent form). This study will be shared publicly and disseminated through publication in peer-reviewed journals, conference presentations and social media outlets.

## Discussion

This protocol details the methodology for the SPACES trial—a two-arm, multi-site, pragmatic, powered RCT, assessing the clinical and cost-effectiveness of a co-produced and tailored PA intervention for people with SMI across NHS mental health trusts in England and Wales.

This study addresses a critical health need by exploring strategies to increase PA and therefore reduce the persistent health disparities between those with SMI and the wider population. Traditional approaches to improving physical health and QoL in this group have often fallen short.^9^ While prior research on PA interventions for people with SMI has shown promise in supporting behaviour change,^23^ many studies have been limited by small sample sizes and other design constraints. Furthermore, there is limited evidence on the feasibility and acceptability of delivering such interventions through NHS care pathways.

A limitation of the study design is the potential heterogeneity of TAU in the control group which may encompass widely differing levels of existing PA provision across NHS trusts. Although we are stratifying by site in the randomisation which should ensure a balance within sites and adjusting for site in the statistical analysis, we cannot completely rule out that the observed intervention effect may be confounded by site-level service ecology. A limitation of the study is that it is not possible to blind researchers conducting the follow-up interviews to allocation; this could introduce bias in the self-completion measures (PHQ-9, GAD-7, etc). However, our primary outcome is an objective measure and due to this we have not planned any sensitivity analyses.

This study will determine whether the SPACES PA intervention delivered by NHS staff is effective for people living with SMI and potentially cost-effective for the NHS. Enhancing opportunities for individuals with SMI to adopt healthier, more active lifestyles may help narrow the health inequality gap. Co-produced with people who have lived experience of SMI and the professionals who support them, the SPACES intervention is well placed to identify meaningful, effective strategies for promoting sustained PA in this population.

## Supplementary material

10.1136/bmjopen-2025-115528 online supplemental file 1

10.1136/bmjopen-2025-115528 online supplemental file 2

10.1136/bmjopen-2025-115528 online supplemental file 3

10.1136/bmjopen-2025-115528 online supplemental file 4

## References

[1] Hayes JF, Marston L, Walters K (2017). Mortality gap for people with bipolar disorder and schizophrenia: UK-based cohort study 2000-2014. Br J Psychiatry.

[2] Correll CU, Solmi M, Veronese N (2017). Prevalence, incidence and mortality from cardiovascular disease in patients with pooled and specific severe mental illness: a large‐scale meta‐analysis of 3,211,768 patients and 113,383,368 controls. World Psychiatry.

[3] Vancampfort D, Firth J, Schuch FB (2017). Sedentary behavior and physical activity levels in people with schizophrenia, bipolar disorder and major depressive disorder: a global systematic review and meta‐analysis. World Psychiatry.

[4] NHS (2019). The NHS Long Term Plan. https://www.longtermplan.nhs.uk/wp-content/uploads/2019/08/nhs-long-term-plan-version-1.2.pdf.

[5] Biswas A, Oh PI, Faulkner GE (2015). Sedentary Time and Its Association With Risk for Disease Incidence, Mortality, and Hospitalization in Adults. Ann Intern Med.

[6] WHO (2019). Physical inactivity: a global public health problem. https://www.who.int/dietphysicalactivity/factsheet_inactivity/en.

[7] Public Health Wales Supporting healthy behaviours. https://phw.nhs.wales/services-and-teams/primary-care-division/supporting-healthy-behaviours.

[8] Mishu MP, Peckham EJ, Heron PN (2019). Factors associated with regular physical activity participation among people with severe mental ill health. Soc Psychiatry Psychiatr Epidemiol.

[9] Firth J, Rosenbaum S, Stubbs B (2016). Motivating factors and barriers towards exercise in severe mental illness: a systematic review and meta-analysis. Psychol Med.

[10] Firth J, Siddiqi N, Koyanagi A (2019). Keeping the body in mind.

[11] Stubbs B, Vancampfort D, Hallgren M (2018). EPA guidance on physical activity as a treatment for severe mental illness: a meta-review of the evidence and Position Statement from the European Psychiatric Association (EPA), supported by the International Organization of Physical Therapists in Mental Health (IOPTMH). Eur Psychiatry.

[12] Machaczek KK, Firth J, Tew GA (2023). Towards the standardization of physical activity programs for severe mental ill health: A survey of current practice across 54 mental health trusts in England. Psychiatry Res.

[13] Jones G, Bailey L, Beeken RJ (2024). Supporting physical activity through co-production in people with severe mental ill health (SPACES): protocol for a randomised controlled feasibility trial. Pilot Feasibility Stud.

[14] Leamy M, Bird V, Le Boutillier C (2011). Conceptual framework for personal recovery in mental health: systematic review and narrative synthesis. Br J Psychiatry.

[15] Bowes E, Burke T, Faires M (2025). Better together: Service user and delivery staff experiences of the SPACES physical activity intervention for people with severe mental illness - a qualitative study of a feasibility trial. Ment Health Phys Act.

[16] Gov UK Mental capacity act 2005. https://www.legislation.gov.uk/ukpga/2005/9/contents.

[17] Smelt AFH, van der Weele GM, Blom JW (2010). How usual is usual care in pragmatic intervention studies in primary care? An overview of recent trials. Br J Gen Pract.

[18] Gov UK (2019). Chief Medical Officers’ Physical Activity Guidelines.

[19] Williams J, Stubbs B, Richardson S (2019). “Walk this way”: results from a pilot randomised controlled trial of a health coaching intervention to reduce sedentary behaviour and increase physical activity in people with serious mental illness. BMC Psychiatry.

[20] Ryan RM, Deci EL (2000). Self-determination theory and the facilitation of intrinsic motivation, social development, and well-being. Am Psychol.

[21] Michie S, Richardson M, Johnston M (2013). The behavior change technique taxonomy (v1) of 93 hierarchically clustered techniques: building an international consensus for the reporting of behavior change interventions. Ann Behav Med.

[22] Tew GA, Bailey L, Beeken RJ (2023). Physical Activity in Adults with Schizophrenia and Bipolar Disorder: A Large Cross-Sectional Survey Exploring Patterns, Preferences, Barriers, and Motivating Factors. Int J Environ Res Public Health.

[23] Walker L, Dawson S, Brady S (2023). Co-producing a physical activity intervention with and for people with severe mental ill health – the spaces story. Qualitative Research in Sport, Exercise and Health.

[24] Johnston M, Carey RN, Connell Bohlen LE (2021). Development of an online tool for linking behavior change techniques and mechanisms of action based on triangulation of findings from literature synthesis and expert consensus. Transl Behav Med.

[25] Heart Online Rating of perceived exertion: borg scales. https://www.heartonline.org.au/media/DRL/Rating_of_perceived_exertion_-_Borg_scale.pdf.

[26] Axivity Axivity ax6. https://axivity.com/product/ax6.

[27] van Hees V, Migueles J, Fang Z GGIR: raw accelerometer data analysis. https://cran.r-project.org/web/packages/GGIR/index.html.

[28] Migueles JH, Rowlands AV, Huber F (2019). GGIR: A Research Community–Driven Open Source R Package for Generating Physical Activity and Sleep Outcomes From Multi-Day Raw Accelerometer Data. Journal for the Measurement of Physical Behaviour.

[29] Team (2024). R: a language and environment for statistical computing. https://www.R-project.org/.

[30] Kroenke K, Spitzer RL, Williams JBW (2001). The PHQ-9: validity of a brief depression severity measure. J Gen Intern Med.

[31] Spitzer RL, Kroenke K, Williams JBW (2006). A brief measure for assessing generalized anxiety disorder: the GAD-7. *Arch Intern Med*.

[32] EuroQol Research Foundation EQ-5d-5l user guide: basic information on how to use the EQ-5D-5L instrument. https://euroqol.org/information-and-support/documentation/user-guides.

[33] National Institute for Health and Care Excellence (NICE) (2022). NICE health technology evaluations: the manual [internet]. process and methods (PMG36). https://www.nice.org.uk/process/pmg36/chapter/introduction-to-health-technology-evaluation.

[34] Hernández Alava M, Pudney S, Wailoo A (2023). Estimating the Relationship Between EQ-5D-5L and EQ-5D-3L: Results from a UK Population Study. Pharmacoeconomics.

[35] Brazier J, Connell J, Papaioannou D (2014). A systematic review, psychometric analysis and qualitative assessment of generic preference-based measures of health in mental health populations and the estimation of mapping functions from widely used specific measures. Health Technol Assess.

[36] Hughes ME, Waite LJ, Hawkley LC (2004). A Short Scale for Measuring Loneliness in Large Surveys: Results From Two Population-Based Studies. Res Aging.

[37] Conner MNP, Conner MSP (2005). Predicting Health Behaviour.

[38] Vlachopoulos SP, Ntoumanis N, Smith AL (2010). The basic psychological needs in exercise scale: Translation and evidence for cross‐cultural validity. Int J Sport Exerc Psychol.

[39] Ekelund U, Tarp J, Steene-Johannessen J (2019). Dose-response associations between accelerometry measured physical activity and sedentary time and all cause mortality: systematic review and harmonised meta-analysis. BMJ.

[40] Gov.UK UK GDPR and data protection act 2018. https://www.gov.uk/data-protection.

[41] Hopewell S, Chan AW, Collins GS (2025). CONSORT 2025 statement: updated guideline for reporting randomised trials. BMJ.

[42] Clark TP, Kahan BC, Phillips A (2022). Estimands: bringing clarity and focus to research questions in clinical trials. BMJ Open.

[43] Lawrance R, Degtyarev E, Griffiths P (2020). What is an estimand & how does it relate to quantifying the effect of treatment on patient-reported quality of life outcomes in clinical trials?. J Patient Rep Outcomes.

[44] Walters SJ (2009). Quality of Life Outcomes in Clinical Trials and Health-Care Evaluation A Practical Guide to Analysis and Interpretation.

[45] NHS Business Services Authority Prescription cost analysis – England – 2021/22. 2022. https://www.nhsbsa.nhs.uk/statistical-collections/prescription-cost-analysis-england/prescription-cost-analysis-england-202122.

[46] Jones K, Weatherly H, Castelli A (2022). Unit costs of health and social care 2022 manual.

[47] NHS Business Services Authority English prescribing dataset. https://www.nhsbsa.nhs.uk/prescription-data/prescribing-data/english-prescribing-data-epd.

[48] Fenwick E, Claxton K, Sculpher M (2001). Representing uncertainty: the role of cost-effectiveness acceptability curves. Health Econ.

[49] Severens JL, De Boo TM, Konst EM (1999). Uncertainty of incremental cost-effectiveness ratios. A comparison of Fieller and bootstrap confidence intervals. Int J Technol Assess Health Care.

[50] Carroll C, Patterson M, Wood S (2007). A conceptual framework for implementation fidelity. Implement Sci.

[51] Greenhalgh T, Robert G, Macfarlane F (2004). Diffusion of innovations in service organizations: systematic review and recommendations. Milbank Q.

[52] Bellg AJ, Borrelli B, Resnick B (2004). Enhancing treatment fidelity in health behavior change studies: best practices and recommendations from the NIH Behavior Change Consortium. Health Psychol.

[53] Braun V, Clarke V (2019). Reflecting on reflexive thematic analysis. Qualitative Research in Sport, Exercise and Health.

