# Supplementary material for: Supporting physical activity through co-production in people with severe mental illness (SPACES): study protocol for a pragmatic, parallel-group randomised controlled trial
Source: BMJ Open. 2026 Oct 8;16(10):e115528. doi: 10.1136/bmjopen-2025-115528 (PMC13656100; doi:10.1136/bmjopen-2025-115528)
Supplement: Supplementary file 1 [file bmjopen-16-10-s004.pdf]

Supporting Physical Activity through Co-production in people with Severe Mental Illness (SPACES): a pragmatic randomised controlled trial

Statistical Analysis Plan

Version 2.0

Based on Protocol 2.0 08/05/2026

ISRCTN23019026

Authored By

[redacted]

Approved By

[redacted]

SAP Version History

Changes will be recorded if and when version 2.0 or later versions are being developed.

Version Number	Date	Justification	
2.0	10 August 2026	Change to sample size and power of trial, redefinition of primary outcome (number of days of valid accelerometer data), details of CACE analysis.	

Contents

1 Introduction ... 8
1.1 Background and rationale ... 8
1.2 Objectives ... 8
2 Methods ... 9
2.1 Trial Design ... 9
2.1.1 Randomisation & Blinding ... 9
2.1.2 Sample Size ... 9
2.2 Data sources ... 10
2.3 Timing of analyses ... 11
2.3.1 Interim Analyses ... 11
2.3.2 Final Analysis ... 11
2.3.3 Timing of Outcome Assessments ... 11
3 Statistical Principles ... 13
3.1 Confidence Intervals ... 13
3.2 Adherence and Protocol Deviations ... 13
3.3 Analysis Populations ... 13
4 Description of the trial Population ... 15
4.1 Recruitment ... 15
4.2 Withdrawal and Follow-Up ... 16
4.3 Baseline Characteristics ... 16
5 Statistical Analysis ... 18
5.1 Outcome Definitions ... 19
5.1.1 Primary Outcomes ... 19
5.1.2 Other physical activity related outcomes ... 19
5.1.3 Secondary outcomes ... 20
5.2 Estimands framework ... 24
5.3 Primary effectiveness analysis ... 26
5.3.1 Primary analysis model ... 26
5.3.2 Model Checking ... 27
5.4 Analysis of Other Physical Activity and Secondary Efficacy Outcomes ... 27
5.5 Subgroup analysis ... 28

5.6 Missing data 28
5.6.1 Missing baseline data 29
5.7 Additional analyses 30
5.8 Safety and adverse events 30
5.9 Statistical Software 31
References 32
Appendix 1 Example Tables and Figures 36

List of Abbreviations

AE	Adverse Event
BMI	Body Mass Index
BPNES	Basic Psychological Needs in Exercise Scale
CACE	Complier average causal effect
CI	Confidence Interval
CMHT	Community mental health team
CONSORT	Consolidated Standards of Reporting Trials
CTRU	Clinical Trials Research Unit
DMEC	Data Monitoring and Ethics Committee
DSM-IV	Diagnostic and Statistical Manual of Mental Disorders, 4th edition
EQ5D	EuroQol 5D Questionnaire
GAD-7	Generalised Anxiety Disorder
GP	General practitioner
HEAP	Health Economics Analysis Plan
ICC	Intraclass correlation coefficient
ICD	International Classification of Diseases
ICH	International Conference of Harmonisation
IQR	Interquartile Range
ISRCTN	International Standard Randomised Controlled Trial Number
ITT	Intention to treat
MVPA	Moderate to Vigorous Physical Activity
OMGUI	Open Movement Graphical User Interface
PBQ	Predictive Behaviour Questionnaire
PHQ-9	9-item Patient Health Questionnaire
PMG	Project management group
QoL	Quality of Life
ReQoL	Recovering Quality of Life Questionnaire
RCT	Randomised Controlled Trial
SAE	Severe Adverse Event
SAP	Statistical Analysis Plan
SD	Standard Deviation

SIMPAQ	Simple Physical Activity Questionnaire
SMI	Severe Mental Illness
SOP	Standard Operating Procedure
SPACES	Supporting Physical Activity through Co-production in People with Severe Mental Illness
TPB	Theory and Planned Behaviour
UCLA	University of California, Los Angeles
VAS	Visual analogue scale
WS	Work Stream

Summary Table

**Study title**	Supporting Physical Activity through Co-production in people with Severe Mental Illness (SPACES): a pragmatic randomised controlled trial.
**Trial design**	Pragmatic randomised controlled trial.
**Trial participants**	**Inclusion**<br>• Aged 18 years or older<br>• ICD-10 or DSM-V diagnosis of SMI as documented in GP or psychiatric notes (schizophrenia (F20 and subcodes); schizoaffective disorder (F25 and subcodes); other psychotic and delusional disorders (not of organic origin) (F21-F24 and F26-F29 and subcodes); bipolar and manic episodes (F30 and F31 and subcodes)).<br>• Are able to walk unaided<br>• Willing to wear an accelerometer<br>**Exclusion**<br>• People who lack capacity to participate<br>• Primary diagnosis of drug or alcohol abuse<br>• Medical contraindication of physical activity<br>• Already physically active, defined as >300 minutes/week of self-reported moderate-to-vigorous physical activity (MVPA)<br>• Non-English speakers<br>• People who took part in the feasibility study
**Sample size**	550
**Intervention & control groups**	Control and intervention groups: Both groups will receive usual care.<br>Intervention group only: In addition, the intervention group will receive the offer of an 18-week group-based intervention and up to 6 one-to-one sessions (4 of these sessions can be split into 2), designed to increase physical activity and reduce sedentary behaviour.
**Primary outcome(s)**	6-month post randomisation accelerometer-assessed minutes of Moderate to Vigorous Physical Activity (MVPA) per day (defined as a minimum of 16-hour a day wear time for at least 3 days over the 10-day collection period).
**Secondary outcome(s)**	Physical activity and sedentary behaviours (accelerometer-derived, SIMPAQ), body mass index (BMI), depression (PHQ-9), anxiety (GAD-7), loneliness (UCLA 3-item loneliness scale), health-related quality of life (EQ5D, REQoL), predictive behaviour questionnaire and healthcare resource use, measured at 3, 6 and 12 months.
**Duration of follow-up**	12 months post-randomisation with follow-up at 3-, 6- and 12-months post-randomisation.

1 Introduction

This Statistical Analysis Plan (SAP) is written in conjunction with the International Conference of Harmonisation (ICH) topic E9 on statistical principles for clinical trials [1], guidelines on clinical trials SAPs [2] applicable Standard Operating Procedures (SOPs) from the Sheffield Clinical Trials Research Unit (CTRU), particularly ST001 [3], and the trial protocol and related amendments.

1.1 Background and rationale

People living with severe mental ill health (SMI) (e.g., schizophrenia, bipolar disorder or schizoaffective disorder) die on average 15-20 years earlier than do those who do not have these conditions [4], and over 70% of these deaths are attributed to preventable physical health conditions such as cardiovascular disease [5]. Whilst life expectancy in the general population has steadily increased over recent decades, life expectancy for people with SMI has declined [6]. Addressing this widening health inequality is a priority in the National Health Service (NHS) Long-Term Plan [7].

Previous research has demonstrated that people with SMI engage in substantially less physical activity and more sedentary behaviour than those who do not have SMI [6]. Additionally, research has shown that people with SMI experience several unique barriers to engaging in physical activity such as increased mental health symptoms, lack of social support, and the side effects of medication [8]. However, evidence about the acceptability, feasibility, and effectiveness of specific physical activity interventions is lacking.

As part of the SPACES programme, we have co-produced an intervention aimed at increasing physical activity for people with SMI [9]. We have tested this intervention in a feasibility study [10] and found the intervention to be feasible to deliver and acceptable to people with SMI. The next stage is to test the intervention in a full scale randomised controlled trial.

1.2 Objectives

The main aim of this project is to establish the clinical effectiveness of the SPACES intervention for people with SMI compared to usual care.

The secondary objective is to establish the cost effectiveness of the SPACES intervention for people with SMI compared to usual care.

2 Methods

2.1 Trial Design

The trial is a pragmatic parallel group, external randomised controlled trial (RCT). It will be conducted across geographically dispersed NHS mental health trusts and primary care services in England and Wales. Participants will be recruited via community mental health teams (CMHT) and general practitioner (GP) surgeries.

Participants who are recruited to the control arm will receive the usual NHS care as per local protocols. The intervention arm will also receive usual care. In addition, intervention participants will be offered up to 18 weekly group-based physical activity sessions and 6 one-to-one sessions that will be delivered by a trained physical activity coordinator and lasting up to 18 weeks. In the 6 one-to-one sessions, there are 4 long sessions that may be delivered over 2 sessions (totalling up to 10 one-to-one sessions).

Further details on the trial design can be found in the protocol.

2.1.1 Randomisation & Blinding

Full details on the randomisation and blinding procedures are given in the study protocol.

In brief, allocation will be through stratified block randomisation (with random permuted blocks of various integer multiples of size 2), with a separate schedule for each combination of site (up to 22) and diagnosis (four levels: Schizophrenia (F20 and subcodes) vs. Schizoaffective disorder (F25 and subcodes) vs. Other psychotic and delusional disorders (not of known organic origin) (F21 – F24 and F26 – F29 and subcodes) vs. Bipolar disorder and manic episodes (F30 and F31, and subcodes)).

By the nature of the interventions used within this study, it is not possible to blind the participants, clinicians or researchers to the intervention used. The trial statistician(s) will be blind to group allocation until the SAP is signed off. A senior statistician will be unblinded to be able to contribute to Data Monitoring and Ethics Committee (DMEC) meetings.

2.1.2 Sample Size

The primary outcome is the average minutes of MVPA per day at 6-month post-randomisation.

The Ekelund systematic review and harmonised meta-analysis [12] report the dose-response associations between accelerometery-measured physical activity and sedentary time and all-cause

mortality. This estimated that the hazard ratio for all-cause mortality was a 0.70 (0.62 to 0.79) reduction for a 6-minute increase in minutes of MVPA/day compared to 0 (minutes/day). A 30% relative risk reduction in mortality for a 6-minute increase in minutes of MVPA per day is likely to be clinically and practically important.

The initial sample size estimate for the SPACES RCT was based on a target difference of 6 minutes of MVPA between the groups, with a corresponding standard deviation of 22. 90% power and a two-sided 5% significance level were used, and the sample size estimation allowed for up to 15% loss to follow-up. Using an equation described by Julious [12] which assumes an ANCOVA evaluation of a trial with 1:1 allocation, these input parameters led to a sample of 670.

This estimate has been refined in light of outcomes from the SPACES feasibility trial. From the feasibility trial, the observed standard deviation of minutes of MVPA per day at six months was 28.2 (95% CI: 23.1 to 36.2). This was based on all valid MVPA readings at six months (n=42). In the feasibility study, the estimated correlation between the baseline and 6-month MVPA outcome for the 40 participants with valid accelerometer data at both assessments was 0.60 (95% CI: 0.35 to 0.77). Further, it was deemed appropriate to increase the attrition rate in estimating the sample size of the RCT to 20%, as the loss to follow-up in the feasibility trial was higher than the initially proposed 15%. The feasibility study did not indicate evidence of a relevant amount of intracluster correlation. Although the SPACES intervention is delivered by a trained Physical Activity Coordinator (PACs) at each site, and the outcomes may be clustered by site, we found no evidence of this in the pilot study and are not allowing for the effect of clustering in the sample size calculation (but will include site as random effect in the analysis).

Assuming a target difference of 6 minutes of MVPA/day, a SD of 28, equivalent to a small standardised effect size of 0.21, a baseline to the 6-month post-randomisation correlation of 0.60 in the primary outcome measure, 90% power, and 5% two-sided significance, then 588 participants (294 per group) would be required using an equation described by Julious (2023) which assumes an ANCOVA evaluation of a trial with clustering in one arm only and with 1:1 allocation [11]. Assuming 20% participant attrition, we would need to recruit and randomise 736 participants (368 per arm).

The recruitment and randomisation of participants to the trial was slower than anticipated. Following a review of recruitment and after discussions with the funder (NIHR) it was agreed to revise the target sample size downwards by reducing the power to 80% (compared to 90%) with all other parameters in the sample size calculation being unchanged. The revised target sample size **is 550.** The sample-size amendment was made without access to comparative outcome data by randomised group.

Assuming a target difference of 6 minutes of MVPA/day; a SD of 28, equivalent to a small standardised effect size of 0.21, a baseline to 6-month post randomisation correlation of 0.60 in the primary outcome measure and 80% power and 5% two-sided significance then 440 participants (220 per group) would be required. Assuming 20% participant attrition, we would need to recruit and randomise 550 participants (275 per arm) .

2.2 Data sources

The randomisation list will be accessed via the Sheffield CTRU's randomisation system (SCRAM). Manual data entry from the CRFs onto the CTRU's in-house clinical data management system (Prospect) will occur throughout the project. Accelerometer-collected data will be processed in R statistical software version 4.2.1 [13] using the 'GGIR' package version 2.9-0 [14] and then imported to Prospect by the data management team (see Section 5.1.1 and the accelerometer data processing protocol for further information). The data management team in the Sheffield CTRU will validate and query electronic data for inconsistencies during the trial (as stipulated in SOP DM005). The trial statistician will conduct any additional validation checks where appropriate before the data lock and sign-off (as guided by DM005 and DM012).

2.3 Timing of analyses

2.3.1 Interim Analyses

There is no planned interim analysis beyond checking the recruitment rate at the end of the internal pilot phase. The DMEC will review descriptive statistics of accumulating outcome data prepared by the data management team in the CTRU, including adverse events, by arm.

2.3.2 Final Analysis

The final analysis will take place after participants have completed their 12-month follow-up and sufficient time has been allowed for data collection and cleaning. All data will be analysed collectively.

2.3.3 Timing of Outcome Assessments

Data collection will take place across four-time points; baseline, 3-, 6- and 12-month follow-up post randomisation. Table 1 shows the outcome measures and different time points for collecting these data. A detailed description of the outcome assessments can be found in Section 5.1.

Table 1 Participant assessment and time point requirements throughout the study.

Assessment	Time required	Timeline (months post randomisation)			
		Baseline	3-month Follow-up	6-month Follow-up	12-month Follow-up
**Eligibility and consent**					
Eligibility	NA	X			
Consent	15 minutes	X			
**Background**					
Relevant contact details	5 minutes	X			
Body Mass Index	5 minutes	X	X	X	X
**Mental health details**					
Mental health history		X			
Current mental health status		X	X	X	X
Current medications		X	X	X	X
Referrals to mental health services		X	X	X	X
Admissions to hospital related to mental health		X	X	X	X
**Physical Activity**					
Accelerometer data collection	10 days	X	X	X	X
Adverse event reporting		Ongoing collection			
**Questionnaires**					
Patient Health Questionnaire (PHQ-9)		X	X	X	X
Generalised Anxiety Disorder- 7 (GAD-7)		X	X	X	X
Simple Physical Activity Questionnaire (SIMPAQ)		X	X	X	X
Health-state utility (EuroQol - EQ5D)		X	X	X	X
Recovering Quality of Life (ReQol)		X	X	X	X
UCLA 3-item Loneliness Scale		X	X	X	X
Healthcare resource use		X	X	X	X
Predictive behaviour questionnaire		X	X	X	X

3 Statistical Principles

3.1 Confidence Intervals

We will use a statistical significance level of 5%. Where appropriate, the differences in mean outcomes between the two treatment arms will be presented with their 95% CIs for continuous outcomes. For categorical outcomes, the contrast or differences between groups will be estimated using odds ratios, or some other appropriate metric, together with their 95% CIs.

3.2 Adjustments for Multiplicity

No adjustments for multiplicity will be made to any of the analyses. This acknowledges that the trial has a single, clearly defined primary endpoint. All secondary endpoints are only interpreted in conjunction with the primary endpoint.

3.3 Adherence and Protocol Deviations

Engagement with the courses will be presented as the average number of group and individual sessions attended, and the average group sizes for each of the 18 scheduled group sessions.

We will also show the number and percentage of participants who fall into the following engagement categories:

- attended all sessions (including both group and individual sessions),
- attended >70% of sessions (including both group and individual sessions),
- attended at least 50% of both group and individual sessions (at least 9 group and 3 individual sessions),
- attended at least 50% of the group or individual sessions,
- attended at least one session (including both group and individual sessions).

3.4 Analysis Populations

We will analyse the data on an as randomised (intention-to-treat – ITT) basis, whereby all participants recruited in the study with a baseline and least one valid follow-up outcome datapoint will be included

in the analysis, regardless of their adherence to the protocol. Valid wear time is defined as a minimum of 16-hour wear time for at least 3 days over the 10-day collection period.

In addition, we will conduct a complier average causal effects analysis (CACE) on the primary outcome to explore how adherence to the intervention affects its effectiveness [15] [16].

For participants randomised to the SPACES intervention, we will explore two definitions of compliance, as defined below:

- CACE1: Intervention group participants will be considered compliant if they attended at least one session (this can be either an individual session or a group session),
- CACE2: Intervention group participants will be considered compliant if they attended at least 50% of both group and individual sessions (at least 9 group sessions and 3 individual sessions).

Participants randomised to the control group do not have access to the SPACES intervention and will be assumed to be adherent with usual care.

3.5 Timing of outcome assessments

The analysis models will use protocol stipulated time points in the analysis model (i.e. 3, 6, 12 months), regardless of the actual time of data completion.

If an assessment is closer to a previous or subsequent assessment timepoint than the one it was entered onto the database under, they may be moved to that assessment if no data were collected for this timepoint. (i.e. if the accelerometer data entered for 3 months were collected at 5 months, and no 6-month accelerometer data were available, then these data would be treated as if they were collected at the 6-month follow-up.)

Moving accelerometer data between time points will be based on the start date of the period the accelerometer is worn for, and will be considered if these fall outside the following time windows:

- ≥ 0 and < 4.5 months to the 3-month protocol stipulated follow up time point
- ≥ 4.5 and < 9 months to the 6-month protocol stipulated follow up time point
- ≥ 9 and < 15 months to the 12-month protocol stipulated follow up time point

An average month is 365/12 is 30.42 days. In our calculations, the following number of days will be used:

- ≥ 0 and < 137 days to the 3-month protocol stipulated follow up time point

- ≥ 137 days and < 274 days to the 6-month protocol stipulated follow up time point
- ≥ 274 days and < 456 days to the 12-month protocol stipulated follow up time point

If more than 15% of data fall outside the above listed data collection window, additional sensitivity analysis excluding these observations, will be performed for the primary outcome only.

4 Description of the trial Population

4.1 Recruitment

Using guidelines from the CONSORT statement [13], the summaries outlined in Table 2 will be calculated to construct a CONSORT flowchart. Data will be presented overall and by the treatment arm to show if any differences are present due to treatment allocation. An example CONSORT flowchart is shown in Appendix 1.

Table 2: *CONSORT flowchart summary*

**Screening**	Number initially screened & screening route
**Eligibility**	Number and proportion eligible (relative to those screened).<br>Number and proportion ineligible & reasons.
**Consent**	Number and proportion consented (relative to those eligible).<br>Number and proportion not consented & reasons.
**Randomisation**	Number and proportion randomised to each treatment including the number who received/did not receive each treatment.
**Withdrawal/Follow-Up**	Number and proportion followed-up at 3-, 6- and 12- months follow-up.<br>Number and proportion not followed-up at 3, 6 and 12- months follow-up.<br>Number and proportion with available MVPA data at 3, 6, and 12-months follow-up.<br>Number and proportion included in primary outcome analysis.<br>Number and proportion excluded from primary outcome analyses with reasons.

4.2 Withdrawal and Follow-Up

Details of potential reasons for withdrawal can be found in the protocol (Section 8.2.2) and summaries of these reasons plus any additional reasons found within the trial will be presented overall and split by treatment arm. Withdrawal numbers will also be summarised depending on whether the participant has withdrawn from the treatment and/or from the 3- , 6- or 12-month follow-up.

4.3 Baseline Characteristics

Baseline demographics will be described and summarised overall and by treatment arm. Appendix 1 gives an example of how this will be presented.

Table 3 lists the variables to be presented. For continuous data, mean and standard deviation (SD), as well as median, interquartile range (IQR), and minimum and maximum will be presented. Categorical variables will be summarised using numbers and related percentages in each category.

No statistical significance testing will be used to test baseline imbalances between the two groups, but any noteworthy differences will be descriptively reported.

Table 3: *Baseline characteristics*

**BMI (kg/m^2)**
Height (cm)
Weight (kg)
**Demographics**
Age (years)
Sex at birth (male vs. female)
Gender identity (man vs. woman vs prefer to self-describe vs. prefer not to say)
Ethnicity (options listed with additional free text)
Highest education qualification
Employment status
Approximate gross (before tax) household income
**Health risk behaviours**
Smoking status (never smoked vs current smoker vs previous smoker)

If current smoker

- Product type
- Cigarettes per day (1-10 vs 10-20 vs 20+)

Previous smoker

- When stopped smoking (within last year vs. 1 to 5 years ago vs. 5+ years ago)

Portions of fruit and vegetables per day

Consume carbonated soft drinks (yes vs no)

If yes (consume carbonated drinks)

- Carbonated soft drinks per day

Mental Health Status

Most recent SMI diagnosis

Condition (currently well vs. currently unwell vs. unsure)

Accelerometer-derived outcomes

Minutes of moderate or vigorous physical activity (MVPA) per day

Minutes of vigorous physical activity per day

Minutes of moderate physical activity per day

Minutes of light intensity physical activity per day

Inactive minutes per day

Mean acceleration

Intensity gradient

Questionnaires (Full details of scoring and interpretation, and any prespecified cut offs, are described in Section 5.1 and listed in Appendix 2)

SIMPAQ

- Average hours in bed per night
- Average hours sedentary per day
- Average hours walking per day
- Average hours of sport/exercise per day
- Average hours of other activities per day

PHQ-9

- Total score
- Proportion in each category (none (0 to 4), mild depression (5 to 9), moderate depression (10 to 14), moderately severe depression (15 to 19), and severe depression (20 to 27))

GAD-7

- Total score
- Proportion in each category (no (0 to 4), mild (5 to 9), moderate (10 to 14), and severe anxiety (15 to 21))

EQ-5D

- EQ-5D-5L score
- VAS score

ReQoL

- ReQoL-10 index score
- ReQoL-20 overall index score

UCLA 3-item loneliness scale

- Total score

Predictive behaviour questionnaire

- BPNES Autonomy score
- BPNES Competence score
- BPNES Relatedness score
- TPB Instrumental Attitude score
- TPB Experiential Attitude score
- TPB Injunctive Norm score
- TPB Descriptive Norm score
- TPB Autonomy score
- TPB Capacity score
- TPB Intention score

5 Statistical Analysis

This pragmatic parallel-group randomised controlled trial will be reported and presented in accordance with the CONSORT 2025 statement [17] [18].

5.1 Outcome Definitions

5.1.1 Primary Outcomes

The primary outcome will be objective accelerometer-derived minutes of MVPA per day at 6 months post randomisation (a minimum of 16-hour a day wear time for at least 3 days will be accepted as valid wear time over the 10-day collection period).

Data on physical activity is collected by Axivity AX6 wrist-worn accelerometers, worn on either wrist (i.e., not limited to the dominant hand). A minimum of 16-hour a day wear time for at least 3 days will be accepted as valid wear time over the 10-day collection period. Intensity gradient cut-off points of <30mg, 30-100mg, 101-400mg and 401>mg (inactive, light, moderate and vigorous activity respectively) will be used [19] [20] [21]. Mean MVPA per day is calculated on 5-second epochs with a 1-minute bout duration, inclusion criteria of >80% (which defines what fraction of a bout needs to be above the relevant intensity threshold).

The accelerometers will utilise 100-Hz measurement frequency for a measurement period of 10 days, starting at 12 am the day after a participant clinic visit. OMGUI software will be used by site staff to download data from the watches after each 10-day wear period before secure transfer to the central study team. Data will be further processed in *R* [13] using the GGIR version 2.9-0 [14]. This involves selecting relevant variables, making timepoint names consistent (Baseline, 3 months, 6 months, 12 months), adding site codes, adjusting the precision of continuous variables, identifying reinitialised data and creating separate datasets for each timepoint. We also perform checks on the data for the validity of wear time and to ensure the variables are within their relevant range of values. Further details on this software and the process of deriving accelerometer data are available in the Accelerometer data processing protocol.

5.1.2 Other physical activity related outcomes

Data on physical activity (light physical activity and MVPA) and sedentary behaviour will be collected by Axivity AX6 wrist worn accelerometers at baseline, 3-, 6- and 12-months post randomisation and checks are performed similarly to Section 5.1.1. The following measures of physical activity will be reported:

- Combined minutes of MVPA per day,
- Minutes of vigorous physical activity per day,
- Minutes of moderate physical activity per day,

- Minutes of light-intensity physical activity per day,
- Inactive minutes per day (time spent sedentary),
- Mean acceleration,
- Intensity gradient,

using mean per day over all available valid days (See 5.1.1 for calculation details).

The Simple Physical Activity Questionnaire (SIMPAQ) [22] will be used to measure self-reported physical activity and sedentary behaviour at baseline, 3-, 6- and 12-months post randomisation. The SIMPAQ is a brief measure of physical activity and sedentary behaviour that can be reliably and validly administered by health professionals/researchers. The SIMPAQ consists of five questions to provide a snapshot of a 24-hour period representative of the past week. It reports 'Average hours in bed per night', 'Average hours sedentary per day', 'Average hours walking per day', 'Average hours sport/exercise per day', and 'Average hours other activities per day'. Average hours sedentary will be calculated as 24 hours minus the four other scores, where all scores are completed[22]. MVPA, as assessed by the SIMPAQ, is calculated by summing 'Average hours walking per day' and 'Average hours sport/exercise per day' [22].

5.1.3 Secondary outcomes

The following measures will be assessed at baseline, 3-, 6- and 12-months post-randomisation.

Body mass index (BMI): We will calculate BMI by measuring height at baseline and body weight (using portable scales) at baseline and follow-up. The height at baseline will always be used as it is not measured again at subsequent visits. BMI is calculated using the following formula:

$$\text{BMI}\ (\text{kg/m}^2) = \text{Weight}\ (\text{kg})\ /\ \text{Height}\ (\text{m})^2$$

Patient Health Questionnaire-9 (PHQ-9)[23]: The PHQ-9 measures depression severity and response to treatment, utilising a three-point Likert scale across nine items. Values 0, 1, 2, 3 are assigned to the response categories 'Not at all', 'Several days', 'More than half the days' and 'Nearly every day', respectively. Higher scores indicate more severe depressive symptoms. PHQ-9 is measured by a total score on a scale from 0 to 27, with higher scores indicating higher levels of depression.

The total score is generated by summing the scores for the nine questions, dividing by the number of questions answered, multiplying by 9 and rounding to the nearest whole number. The total score will not be calculated if more than 2 items are missing.

The total score can be categorised with respect to increasing depression: none (0 to 4), mild depression (5 to 9), moderate depression (10 to 14), moderately severe depression (15 to 19), and severe depression (20 to 27).

General Anxiety Disorder-7 (GAD-7) [24]:The GAD-7 is a brief measure of generalised anxiety disorder and its severity, utilising a three-point Likert scale across seven items. Values 0, 1, 2, and 3 are assigned to the response categories of 'not at all', 'several days', 'more than half the days', and 'nearly every day', respectively. GAD-7 is measured by a total score on a scale from 0 to 21, by summing the scores for the seven questions, dividing by the number of questions answered, multiplying by 9 and rounding to the nearest whole number.

Higher scores represent increasing severity of anxiety and can be categorised as: no anxiety (0 to 4), mild (5 to 9), moderate (10 to 14) and severe (15 to 21). The scoring algorithm does not provide guidance on handling of missing data. For this study, we have decided that the total score will not be calculated if more than two items (out of the 7) are missing (i.e. the score will be calculated if at least five questions have been answered) [25].

EuroQoL 5D-5L (EQ5D-5L) [26]:The EQ-5D-5L is an instrument for measuring quality of life. It measures five domains of health, including mobility, self-care, usual activities, pain/discomfort, and anxiety/depression, using a five-point Likert scale and a visual analogue scale that measures overall health.

The EQ-5D-5L consists of five questions and the overall EQ-5D-5L score. At the time of the analysis, we will follow the most current NICE guidance [27] on scoring EQ-5D-5L. At the time of writing this statistical analysis plan, this is to use the EuroQol mapping function developed by Hernández *et al.* [28] to derive utility values by mapping from the EQ-5D-3L UK set to EQ-5D-5L. The range of the mapped utility is -0.529 to 0.987, where 1 indicates full health and lower than 0 represents a state worse than death.

The utility score will not be calculated if any of the five items are missing in line with the EQ-5D-5L guidance where there is no single, universally agreed-upon method for handling missing data [26].

EQ-5D VAS is measured on a scale from 0 (worst imaginable health state) to 100 (best imaginable health state).

Recovering Quality of Life (ReQol) [29]: The ReQoL is a new 20-item Patient Reported Outcome Measure (PROM) that has been developed to assess the quality of life for people with different mental health conditions. This measure is not reliant on diagnosis and is valid across a range of conditions for people who use mental health services. As we are recruiting people with a range of diagnoses, the ReQoL has been chosen as a measure of the impact of mental health.

The ReQoL is a 20-item quality of life measure, split into the 10-item index score (first 10 questions) and an overall score. Each question is scored from 'None of the time' to 'Most or all of the time'. There are nine positively worded questions (Q2, Q4, Q5, Q7, Q8, Q10, Q11, Q15 and Q19) scored 0, 1, 2, 3, and 4 corresponding to 'None of the time', 'Only occasionally', 'Sometimes', 'Often', and 'Most or all of the time', respectively. There are eleven negatively worded questions (Q1, Q3, Q6, Q9, Q12, Q13, Q14, Q15, Q16, Q17, Q18, and Q20) scored 4, 3, 2, 1, and 0 corresponding to 'None of the time', 'Only occasionally', 'Sometimes', 'Often', and 'Most or all of the time', respectively.

The ReQoL-10 index score can be calculated by summing the scores for the first 10 questions. If a single question is unanswered in the first 10 questions, the mean value of the other responses can be used to fill the gap. The score will not be calculated if there is more than one unanswered question. The minimum score for ReQoL-10 is 0 and the maximum is 40, where 0 indicates the poorest quality of life and 40 indicates the highest quality of life.

The total score can be calculated by summing the scores for questions 11-20, in line with the scoring algorithm described above, and adding to the ReQoL-10 score to produce the ReQoL-20 overall index score. In the case of ReQoL-20, the minimum score is 0 and the maximum is 80, where 0 indicates the poorest quality of life and 80 indicates the highest quality of life.

If a single question is unanswered between items 11-20, the mean value of the other responses can be used to fill the gap. If more than one question is unanswered, then the overall index score will not be calculated.

UCLA 3-item loneliness scale [30]:The UCLA 3-item Loneliness Scale is an instrument for measuring loneliness. The scale comprises 3 questions that measure three dimensions of loneliness (companionship, feeling left out and isolation) and has three response categories: hardly ever, some

of the time and often, corresponding to scores 1, 2 and 3 respectively. The total score is calculated by summing the scores from the three questions, which ranges from 3 to 9 with a higher score indicating more loneliness. The total score will not be calculated if any item is unanswered.

Healthcare resource use: A bespoke healthcare service use questionnaire will be used to provide information on the participant's use of primary, secondary and community healthcare services, with special attention paid to physical activity and mental health-related services.

This questionnaire will be used in the health economic analysis and is not discussed in detail in this document.

Predictive behaviour questionnaire: A questionnaire designed to predict participant physical activity has been created based on the Theory of Planned Behaviour (TPB) [31] and the Basic Psychological Needs in Exercise Scale (BPNES) [32]. The questionnaire was designed with lived experience co-applicants and PPIE members to ensure appropriate wording and questionnaire length. The questionnaire comprises 26 questions, answered on a 7-point Likert scale. In all responses, higher scores are associated with more favourable attributes. In both questionnaires, the mean scores of each construct (BPNES) or sub-construct (TPB) will be calculated by averaging the reported scores within the (sub-)constructs. For this study, where there are missing items within (sub-) constructs, we will use mean imputation, whereby we will impute missing values with the average score of the participant's remaining items in the (sub-)construct. (Sub-)Construct scores will not be calculated if all items within the (sub-)construct are missing. No overall scores will be calculated. Questionnaire items will be subdivided into the different (sub-) constructs as follows:

BPNES For the BPNES, the 7-point Likert scale is rated from 1 (Not true at all) to 7 (Very true).

Construct	Items
Autonomy	2: I feel like I am free to decide for myself how to do physical activity.<br>5: I frequently feel I have a choice in regard to the physical activity I do.<br>8: I feel confident in being myself when I do physical activity.<br>11: I feel I have the opportunity to choose the way I do physical activity.
Competence	1: Through doing physical activity, I feel a sense of accomplishment.<br>3: I feel successful when I do physical activity.<br>6: I feel physical activity is something which I am good at.<br>9: I feel I am able to do physical activity on a regular basis.
Relatedness	4: When I do physical activity, the people I do it with are friendly.

	7: I have a good rapport with peers I do physical activity with.<br>10: There are people that I do physical activity with that I consider my friends.

TPB

Construct	Sub-construct	Items
Attitude	Instrumental attitude	12: If I do regular physical activity, this would be... (Bad – Good)<br>13: I feel doing regular physical activity is... (Harmful – Beneficial)<br>14: I feel doing regular physical activity is... (Foolish – Wise)
	Experiential attitude	15: When I do regular physical activity, it is... (Unpleasant – Pleasant)<br>16: I feel doing regular physical activity is... (Unenjoyable – Enjoyable)
Subjective Norm	Injunctive norm	17: I think people generally would approve or disapprove of me doing regular physical activity (Disapprove – Approve)<br>18: People generally think I should or should not do regular physical activity (They think I should not – They think I should)
	Descriptive norm	19: Of the people I know, how many do I know do regular physical activity? (None – All)<br>20: Most people I know do regular physical activity. (Disagree – Agree)
Perceived behavioural control	Autonomy	21: I feel in complete control over whether or not I do regular physical activity. (Disagree – Agree)<br>22: How much control do you have over whether or not you do regular physical activity? (No control – Complete control)
	Capacity	23: If I want to, doing regular physical activity would be... (Difficult – Easy)<br>24: If I wanted to, I could easily do regular physical activity. (Disagree – Agree)
Intention	-	25: I intend to do regular physical activity (Definitely do not – Definitely do)<br>26: How likely is it that you will do regular physical activity? (Very unlikely – Very likely)

5.2 Estimands framework

An estimand is a clear and explicit description of the treatment effect to be estimated in an RCT. It is made up of five connected elements: i) the population, ii) the treatments (we want to compare), iii) the outcome or endpoint, iv) how to account for intercurrent events and a v) population-level summary measures of how the outcome between the different treatment conditions will be compared. The estimands framework described in Table 1 answers the following question:

In people with SMI, what is the difference in the average minutes of MVPA per day (as measured by an accelerometer) between participants randomised to the SPACES intervention/treatment group in addition to usual care, followed by any subsequent therapy/treatment (as needed), compared with participants randomised to the usual care treatment group only followed by any subsequent therapy/treatment (as needed), after 6 months from randomisation or death (whichever occurs first), regardless of study treatment discontinuation?

Table 4: Estimand framework applied to SPACES RCT

**Estimand attribute**	**Description**
Population	Adult patients (aged 18 years or older) with Severe Mental illness (SMI)
Treatment(s)	SPACES intervention in addition to usual care followed by any subsequent therapy/treatment (as needed) compared with participants randomised to usual care treatment group only followed by any subsequent therapy/treatment (as needed)
Outcome (endpoint)	Mean minutes of MVPA per day, measured at 6-months post-randomisation.
Handling Intercurrent events<br>(see below for definitions)	1. Stopping randomised treatment for any reason – treatment policy (as part of treatment)<br>2. Switching treatments – treatment policy (as part of treatment)<br>3. Changing randomised treatment or not receiving randomised treatment - treatment policy (as part of treatment)<br>4. Use of other medications/treatments/therapy - treatment policy (as part of treatment)<br>5. Death – while alive
Summary measures	Difference in mean minutes of MVPA per day between the two randomised groups

Definitions:

A treatment policy strategy to handle intercurrent events means that participants will be analysed in line with their randomised treatment, regardless of the relevant intercurrent event occurring [33].

The while alive policy to handle deaths as an intercurrent event implies that we are interested in the effect on the outcome until death, and that we will include participants' outcome data in the analysis until they die, and not include (or impute) any outcome data thereafter [34].

5.3 Primary effectiveness analysis

See Appendix 1 for examples of the presentation of tables and figures. The observed minutes of MVPA per day will be presented descriptively (e.g. n, mean, standard deviation) in a table for each time point (baseline, 3, 6 and 12 months) by the treatment arm.

We will present the data graphically, using line graphs or boxplots to show how the data differs between treatment arms at different time points.

The number (and percentage) of participants with valid outcome data (defined as a minimum of 16 hours or more a day for a minimum of 3 days, across the ten-day collection period) at each time point will be presented, by the treatment arm and overall.

5.3.1 Primary analysis model

The primary analysis will be on the as randomised (ITT) sample, comparing mean accelerometer-reported minutes of MVPA per day at 6 months post-randomisation between the SPACES intervention and control groups. Between group differences will be estimated from a three-level mixed effect linear model with the randomised group, time point post-randomisation, randomised group by time point interactions, baseline minutes of MVPA per day and diagnosis at baseline (Schizophrenia (F20 and subcodes), schizoaffective disorder (F25 and subcodes), other psychotic and delusional disorders (not of known organic origin) (F21 – F24 and F26 – F29 and subcodes), bipolar disorder and manic episodes (F30 and F31, and subcodes)) as fixed covariates and site and participant as random effects, with the repeated post-randomisation observations for each participant (at 3, 6, and 12 months) (level 1) nested within participants (level 2) nested within sites (level 3) and with a random intercept at the site level (level 3) and a random intercept at the participant level (level 2). To be included in the primary analysis model participants must have valid baseline accelerometery outcome data and at least one valid post-randomisation assessment of outcome (minutes of MVPA per day). Persons delivering the intervention (here: physical activity coordinators (PACs)) are not included as random effects in the model because there are expected to be low numbers of PACs per site and different PACs may deliver different sessions or components of the intervention. Recruiting centre/site is considered the most relevant factor for clustering in this trial and is included in the model.

The randomised group, time point, site and participant are factor variables, and MVPA variables are continuous. We shall assume an exchangeable correlation between the repeated measures and an

exchangeable covariance structure between the random effects for participant and site[35]. Restricted maximum likelihood estimation will be used, and P-values (and confidence intervals) for regression coefficients will be derived from Wald tests (ratio of estimate divided by the standard error of the estimate) and compared to the standard Normal distribution. A package such as lme4 or similar will be used to implement the models [36].

The point estimate and 95% confidence interval (CI) for the difference in mean minutes of MVPA per day, at 6 months post-randomisation, and the other follow-up time points, between the SPACES intervention and control arm from the longitudinal mixed effects linear model will be reported.

5.3.2 Model Checking

The assumptions of the mixed effects linear regression model will be assessed graphically using the following methods:

- We will plot and tabulate the primary outcome over time (baseline, 3m, 6m, 12m) by group for all available data and for complete cases. Complete cases are participants without missing primary outcome data at baseline or any follow-up. We acknowledge that interpretation of the complete case analysis may be limited as a large population of the sample may potentially be excluded (including participants with available primary outcome data).
- Normality checks will be performed using a Normal probability plot of the (standardised) residuals or a histogram of the residuals.
- Homoscedasticity checks will be performed by plotting the residuals against the fitted values.
- If the 3-level mixed effect linear model fails to converge, we will fit a 2-level model ignoring site.

5.4 Analysis of Other Physical Activity and Secondary Efficacy Outcomes

Average minutes of sedentary activity and physical activity as measured by SIMPAQ (defined in Section 5.1.2), and other accelerometer-assessed outcomes (defined in Section 5.1.2) will be analysed in a similar way to accelerometer minutes of MVPA.

All secondary outcomes will be presented and analysed in line with the primary endpoint, as described in Section 5.3.1. Where available, each model will include a covariate for the baseline values of the independent variable instead of baseline MVPA.

The number and percentage of participants with relevant outcome data will be summarised by treatment arm.

5.5 Subgroup analysis

Subgroup analyses will be performed only for the primary endpoint (minutes of MVPA per day at 6 months).

The subgroup analysis will be done using a mixed-effects linear regression model with the primary outcome (minutes of MVPA per day) at 6 months post-randomisation, and the response will be carried out using a model similar to the primary analysis model. This model will only include the 6-month follow-up time point, and no additional follow-up data. We will include an interaction term in the regression model between the randomised intervention group and subgroup to directly examine the strength of evidence for the treatment difference between the treatment groups (Intervention versus Control) varying between subgroups. The estimated regression coefficient and 95% CI for the interaction term(s) will be reported from the regression model. The point estimate and confidence intervals for the subgroups from the regression will be graphically displayed alongside each other in a forest plot. No *P*-values will be presented [37].

Gender (male or female) and diagnosis (psychosis, schizoaffective disorder, bipolar disorder or other SMI) will be the only a priori defined subgroups to be considered. Subgroup analysis will be performed regardless of the statistical significance of the overall intervention effect (Intervention versus Control).

5.6 Missing data

We will report and describe the proportion of missing data in each group and overall and present descriptive statistics for baseline variables by treatment group and missing data status for the primary endpoint only (comparing those with and without MVPA data at 6 months). We will present baseline data by randomised group stratified by those with and without available primary outcome data (i.e. 6-month post randomisation accelerometer-assessed minutes of Moderate to Vigorous Physical Activity (MVPA) per day) and report any notable differences.

The primary analysis assumes a missing at random mechanism (MAR), whereby missing outcome data can be explained by other variables included in the model i.e. randomisation factors, site/centre and baseline and available follow-up MVPA data, but does not depend on the unobserved missing value itself.

Therefore, we will perform sensitivity analyses considering the impact of missing not at random (MNAR) scenarios. Specifically, we will assess how the conclusions from this trial may change if participants with missing 6-month data had outcomes up to 6 minutes of MVPA per day less than assumed under missing at random (MAR). This will allow us to assess how robust our conclusions are in relation to assumptions made about missing data in the primary analysis [38]. Six-minutes of MVPA was chosen in line with the clinically relevant difference used in the sample size estimation.

This missing data sensitivity analysis will be implemented using the 'rctmiss' [39] package in Stata or similar software. The estimates of the treatment effects and their associated confidence intervals from the different analysis approaches will be graphically displayed for the primary outcome alongside the results for the observed data.

5.6.1 Missing baseline accelerometery and MVPA outcome data

Subjects will be included in the primary analysis model provided they have at baseline and least one post-randomisation measurement of MVPA. As part of a sensitivity of the primary outcome, missing baseline MVPA outcome data will be handled by mean imputation, which will be done by the recruiting centre to ensure that all participants with any valid post-randomisation outcome data are included in the analyses [40]. In the event of only one participant per recruiting centre and that participant has missing baseline MVPA outcome data we will use the baseline mean minutes of MVPA per day for the randomised group. Summary statistics of baseline data will be restricted to the observed data only. As described above, some analysis models will be adjusted for baseline data within which missing data have been mean-imputed. This approach will be clarified in all relevant tables and other study outputs.

5.6.2 CACE analysis

As part of a sensitivity analysis for the primary ITT analysis of the primary outcome, we shall also conduct a Complier Average Causal Effects (CACE) analysis. This analysis will only include primary outcome data (minutes of MVPA per day) at the primary follow-up time point (six months post-randomisation). For participants randomised to the SPACES intervention, we will explore two definitions of compliance, as defined below:

- CACE1: Intervention group participants will be considered compliant if they attended at least one session (this can be either an individual session or a group session),
- CACE2: Intervention group participants will be considered compliant if they attended at least 50% of both group and individual sessions (at least 9 group sessions and 3 individual sessions).

Participants randomised to the control group do not have access to the SPACES intervention and will be assumed to be adherent with usual care.

The CACE analysis will use the randomised treatment allocation (exogenous or instrumental variable) and covariates (e.g. age, gender and baseline minutes of MVPA per day) to predict treatment receipt/compliance (endogenous), before using this prediction in place of treatment in the primary analysis model. CACE analysis will use a two-stage least squares regression with age, gender, and baseline minutes of MVPA as covariates and robust standard errors (at each of the two stages) that allow for the clustering by centre. The CACE sensitivity analysis aims to yield estimates of the effects of the SPACES intervention for individuals who complied with treatment.

5.7 Additional analyses

Health economics analyses will be reported in the Health Economics Analysis Plan (HEAP).

The health economist will perform their analysis independently from the statistical team and will receive the same final data exported from the trial database. Numbers included in key analyses, and descriptive statistics for key variables may be compared between both analyses.

5.8 Safety and adverse events

We will summarise all adverse events (AEs) and serious adverse events (SAEs) reported during the 12 months after randomisation outlined in the table below. We will report the number (%) of events, the number (%) of participants with at least one (S)AE and the number (%) of (S)AEs by type. We will also report the number (%) of AEs and SAEs related to intervention and procedure. AE and SAE categories include:

Adverse events	Serious adverse events
• Muscle soreness which fails to resolve 72 hours following participation at a physical activity session,<br>• Substantial increases in pre-existing pain which fails to resolve 72 hours following a physical activity session,	• Death,<br>• Life threatening,<br>• Inpatient hospitalisation,<br>• Prolongs hospitalisation,<br>• Persistent or significant disability or incapacity,<br>• Considered medically significant by the investigator.

- Development of new musculoskeletal pain which fails to resolve 72 hours following a physical activity session,
- Injuries sustained at the site of intervention delivery on the day of intervention delivery,
- Skin irritation as a result of wearing the accelerometer,
- Deterioration in mental health

Other (S)AEs may be categorised according to the free text by the chief investigator if relevant.

For observed SAEs, summary statistics on the seriousness criteria, frequency, intensity, outcome, expectedness and whether the event resulted in hospitalisation will also be reported.

5.9 Statistical Software

This analysis will use suitable packages and software such as R Version 4.5.1 [13] or Stata 18 [41] The software used will be documented in the statistical report.

References

[1] ICH, "ICH E9: Statistical Principles for Clinical Trials," 1998.

[2] C. et al. Gamble, "Guidelines for the content of statistical analysis plans in clinical trials," JAMA, vol. 318, no. 23, pp. 2337–2343, 2017, doi: 10.1001/jama.2017.18556.

[3] Sheffield CTRU, "ST001: The Statistical Analysis Plan." 2021.

[4] J. F. Hayes, L. Marston, K. Walters, M. B. King, and D. P. J. Osborn, "Mortality gap for people with bipolar disorder and schizophrenia: UK-based cohort study 2000-2014," Br. J. Psychiatry, vol. 211, no. 3, pp. 175–181, 2017, doi: 10.1192/bjp.bp.117.202606.

[5] C. U. Correll et al., "Prevalence, incidence and mortality from cardiovascular disease in patients with pooled and specific severe mental illness: a large-scale meta-analysis of 3,211,768 patients and 113,383,368 controls," World Psychiatry, vol. 16, no. 2, pp. 163–180, 2017, doi: 10.1002/wps.20420.

[6] D. Vancampfort et al., "Sedentary behavior and physical activity levels in people with schizophrenia, bipolar disorder and major depressive disorder: a global systematic review and meta-analysis," World Psychiatry, vol. 16, no. 3, pp. 308–315, 2017, doi: 10.1002/wps.20458.

[7] NHS, "The NHS Long Term Plan," 2019, doi: 10.38192/12.1.4.

[8] J. Firth, S. Rosenbaum, B. Stubbs, P. Gorczynski, A. R. Yung, and D. Vancampfort, "Motivating factors and barriers towards exercise in severe mental illness: A systematic review and meta-analysis," Psychol. Med., vol. 46, no. 14, pp. 2869–2881, 2016, doi: 10.1017/S0033291716001732.

[9] L. Walker et al., "Co-producing a physical activity intervention with and for people with severe mental ill health–the spaces story," Qual. Res. Sport. Exerc. Heal., vol. 15, no. 2, pp. 235–247, 2023, doi: 10.1080/2159676X.2022.2161610.

[10] G. Jones et al., "Supporting physical activity through co-production in people with severe mental ill health (SPACES): protocol for a randomised controlled feasibility trial," Pilot Feasibility Stud., vol. 10, no. 1, pp. 1–14, 2024, doi: 10.1186/s40814-024-01460-0.

[11] U. Ekelund et al., "Dose-response associations between accelerometry measured physical activity and sedentary time and all cause mortality: Systematic review and harmonised meta-analysis," BMJ, vol. 366, pp. 1–10, 2019, doi: 10.1136/bmj.l4570.

[12] S. A. Julious, Sample Sizes for Clinical Trials. Second edition. CRC Press, Taylor & Francis Group., 2023.

[13] R Core Team, "R: A Language and Environment for Statistical Computing." 2024. [Online]. Available: https://www.r-project.org/

[14] van H. V. Migueles JH, Rowlands AV, Huber F, Sabia S, "GGIR: A research community-Driven open source R package for generating physical acitivty and sleep outcomes from multi-day raw accelerometer data," J. Meas. Phys. Behav., vol. 2, pp. 188–196, 2019, doi: 10.1123/jmpb.2018-0063.

[15] C. E. Hewitt, D. J. Torgerson, and J. N. V Miles, "Is there another way to take account of noncompliance in randomized controlled trials?," CMAJ, vol. 175, no. 4, pp. 347–348, 2006, doi: 10.1503/cmaj.051625.

[16] G. Dunn, B. Group, and M. Maracy, "Estimating treatment effects from randomized clinical trials with noncompliance and loss to follow-up : the role of instrumental variable methods," Stat Methods Med Res, vol. 14, no. 4, pp. 369–95, 2005, doi: 10.1191/0962280205sm403oa.

[17] K. F. Schulz, D. G. Altman, and D. Moher, "CONSORT 2010 Statement: updated guidelines for reporting parallel group randomised trials," BMJ, vol. 340, p. c332, Mar. 2010, doi: 10.1136/bmj.c332.

[18] D. Moher et al., "CONSORT 2010 explanation and elaboration: Updated guidelines for reporting parallel group randomised trials," Int. J. Surg., vol. 10, no. 1, pp. 28–55, 2012, doi: 10.1016/j.ijsu.2011.10.001.

[19] A. V Rowlands et al., "Accelerometer-assessed Physical Activity in Epidemiology : Are Monitors Equivalent ?," no. 4, pp. 257–265, 2018, doi: 10.1249/MSS.0000000000001435.

[20] B. Matter, A. V Rowlands, T. O. M. Yates, M. Davies, K. Khunti, and C. L. Edwardson, "Raw Accelerometer Data Analysis with," pp. 1935–1941, 2016, doi: 10.1249/MSS.0000000000000978.

[21] M. Hildebrand, V. T. Van Hees, B. Hermann Hansen, and U. Ekelund, "Age Group Comparability of Raw Accelerometer Output from Wrist- and Hip-Worn Monitors," Med. Sci. Sport. Exerc., pp. 1816–1824, 2014, doi: 10.1249/MSS.0000000000000289.

[22] S. Rosenbaum et al., "Assessing physical activity in people with mental illness: 23-country reliability and validity of the simple physical activity questionnaire (SIMPAQ)," BMC Psychiatry, vol. 20, no. 1, pp. 1–12, 2020, doi: 10.1186/s12888-020-2473-0.

[23] K. Kroenke, R. L. Spitzer, and J. B. W. Williams, "The PHQ-9: Validity of a brief depression severity measure," J. Gen. Intern. Med., vol. 16, no. 9, pp. 606–613, 2001, doi: 10.1046/j.1525-1497.2001.016009606.x.

[24] R. L. Spitzer, K. Kroenke, J. B. W. Williams, and B. Löwe, "A brief measure for assessing generalized anxiety disorder: the GAD-7," Arch. Intern. Med., vol. 166, no. 10, pp. 1092–1097, 2006, doi: 10.1001/archinte.166.10.1092.

[25] NHS, "GENERALISED ANXIETY DISORDER SCORE," Data Dictionary. [Online]. Available: https://archive.datadictionary.nhs.uk/DD Release June 2014/data_dictionary/data_field_notes/g/generalised_anxiety_disorder_score_de.asp@shownav=0.html

[26] EUROQoL, "EuroQol Research Foundation. EQ-5D-5L User Guide, 2025." [Online]. Available: https://euroqol.org/information-and-support/documentation/user-guides.

[27] National Institute for Health and Care Excellence (NICE), "NICE health technology evaluations: the manual (PMG36).," Process Methods, no. January, 2022.

[28] M. Hernández Alava, S. Pudney, and A. Wailoo, "Estimating the Relationship Between EQ-5D-5L and EQ-5D-3L: Results from a UK Population Study," Pharmacoeconomics, vol. 41, no. 2, pp. 199–207, 2023, doi: 10.1007/s40273-022-01218-7.

[29] J. Brazier et al., "A systematic review, psychometric analysis and qualitative assessment of generic preference-based measures of health in mental health populations and the estimation of mapping functions from widely used specific measures," Health Technol. Assess. (Rockv)., vol. 18, no. 34, 2014, doi: 10.3310/hta18340.

[30] M. E. Hughes, L. J. Waite, L. C. Hawkley, and J. T. Cacioppo, "A Short Scale for Measuring Loneliness in Large Surveys: Results From Two Population-Based Studies," Res. Aging, vol. 26, no. 6, pp. 655–672, 2004, doi: 10.1177/0164027504268574.

[31] M. Conner and P. Sparks, "The theory of planned behaviour and health behaviours," Predict. Heal. Behav., vol. 2, pp. 170–222, 2005.

[32] S. P. Vlachopoulos, N. Ntoumanis, and A. L. Smith, "The basic psychological needs in exercise scale: Translation and evidence for cross-cultural validity," Int. J. Sport Exerc. Psychol., vol. 8, no. 4, pp. 394–412, 2010, doi: 10.1080/1612197X.2010.9671960.

[33] T. P. Clark, B. C. Kahan, A. Phillips, I. White, and J. R. Carpenter, "Estimands: Bringing clarity and focus to research questions in clinical trials," BMJ Open, vol. 12, no. 1, pp. 1–7, 2022, doi: 10.1136/bmjopen-2021-052953.

[34] R. Lawrance et al., "What is an estimand & how does it relate to quantifying the effect of treatment on patient-reported quality of life outcomes in clinical trials?," J. Patient-Reported Outcomes, vol. 4, no. 1, 2020, doi: 10.1186/s41687-020-00218-5.

[35] M. J. Gurka, L. J. Edwards, and K. E. Muller, "Avoiding bias in mixed model inference for fixed effects Matthew," Stat Med., vol. 30, no. 22, pp. 2696–2707, 2011, doi: 10.1002/sim.4293.Avoiding.

[36] Bate.s D, M. Mächler, B. Bolker, and S. Walker, "Fitting Linear Mixed-Effects Models Using lme4," J. Stat. Softw., vol. 67, pp. 1–48, 2015, doi: 10.18637/jss.v067.i01.

[37] J. Cuzick, "Forest plots and the interpretation of subgroups," Lancet, vol. 365, no. 9467, p. 1308, 2005, doi: 10.1016/S0140-6736(05)61026-4.

[38] I. R. White, N. J. Horton, J. Carpenter, and S. J. Pocock, "Strategy for intention to treat analysis in randomised trials with missing outcome data," Bmj, vol. 342, no. 7803, pp. 910–912, 2011, doi: 10.1136/bmj.d40.

[39] I. White, "RCTMISS: Stata module to analyse a randomised controlled trial (RCT) allowing for informatively missing outcome data." 2017.

[40] I. R. White, P. Royston, and A. M. Wood, "Multiple imputation using chained equations : Issues and guidance for practice," Tutor. Biostat., 2010, doi: 10.1002/sim.4067.

[41] StataCorp, "Stata Statistical Software: Release 18." College Station, TX: StataCorpLLC, 2023.

Appendix 1 Example Tables and Figures

Figure: *CONSORT flow diagram*

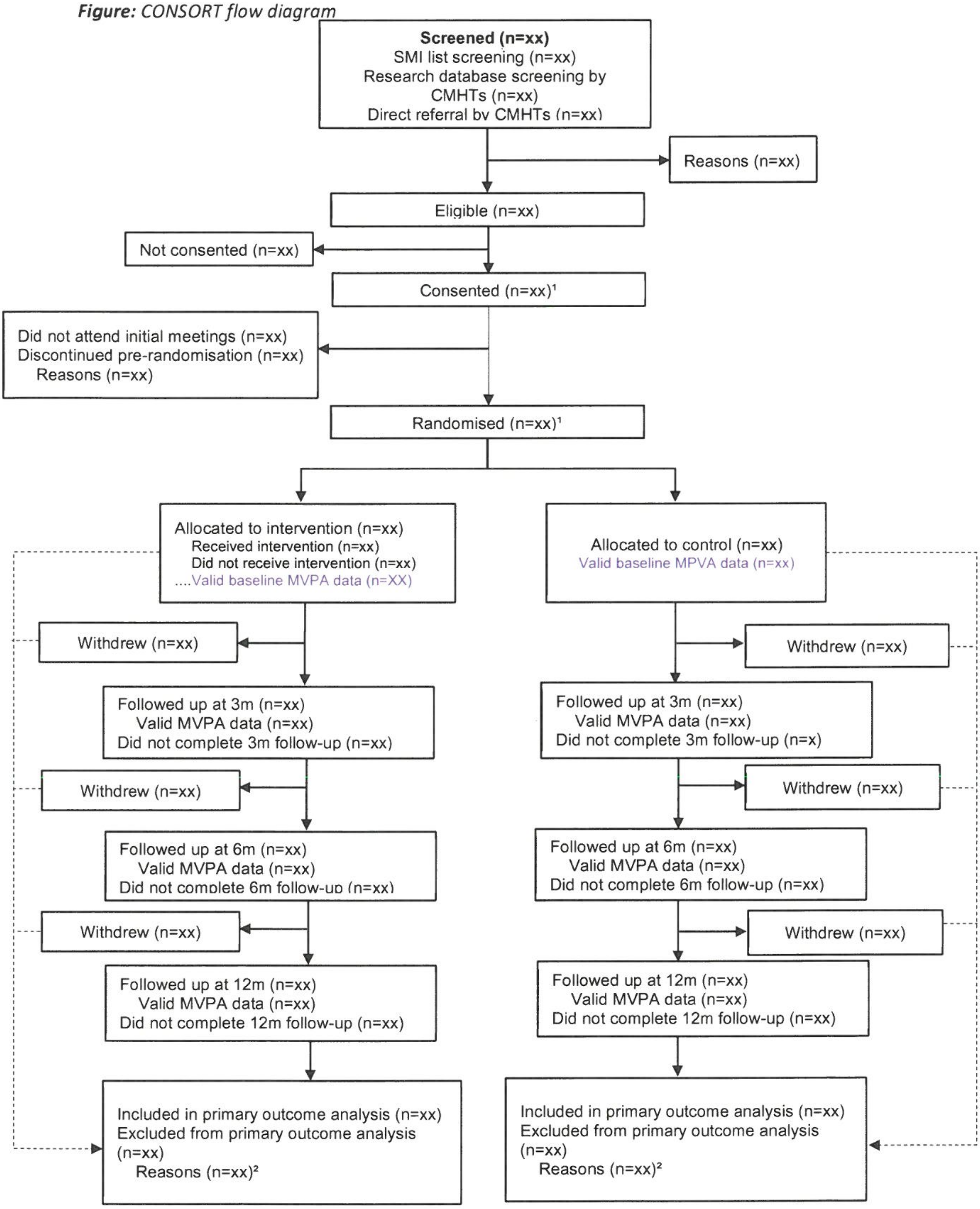

1. If number consented and number randomised are equal, these boxes may be collapsed into a single item: 'Randomised and consented (n=xx)'.
2. Reasons include 'Did not complete any follow-up (n=xx)' and 'No valid baseline MVPA data".

Table: *Baseline characteristics by treatment arm*

	Variable	Scoring	Control (n=xx)	Intervention (n=xx)	All (N=xx)
**BMI**	BMI (kg/m^2)	Mean (SD)	xx (xx)	xx (xx)	xx (xx)
		Median (IQR)	xx (xx)	xx (xx)	xx (xx)
		Min, Max	xx, xx	xx, xx	xx, xx
	Weight (kg)	Mean (SD)	xx (xx)	xx (xx)	xx (xx)
		Median (IQR)	xx (xx)	xx (xx)	xx (xx)
		Min, Max	xx, xx	xx, xx	xx, xx
**Demographics**	Diagnosis	Schizophrenia (F20 and subcodes)	xx (xx%)	xx (xx%)	xx (xx%)
		Schizoaffective disorder (F25 and subcodes)	xx (xx%)	xx (xx%)	xx (xx%)
		Other psychotic and delusional disorders (not of organic origin) (F21-F24 and F26-F29 and subcodes)	xx (xx%)	xx (xx%)	xx (xx%)
		Bipolar and manic episodes (F30 and F31 and subcodes)).	xx (xx%)	xx (xx%)	xx (xx%)
	Age (years)	Mean (SD)	xx (xx)	xx (xx)	xx (xx)
		Median (IQR)	xx (xx)	xx (xx)	xx (xx)
		Min, Max	xx, xx	xx, xx	xx, xx
	Sex at birth	Male	xx (xx%)	xx (xx%)	xx (xx%)
		Female	xx (xx%)	xx (xx%)	xx (xx%)
	Gender identity	Man	xx (xx%)	xx (xx%)	xx (xx%)
		Woman	xx (xx%)	xx (xx%)	xx (xx%)
		Prefer to self-describe	xx (xx%)	xx (xx%)	xx (xx%)
		Prefer not to say	xx (xx%)	xx (xx%)	xx (xx%)
	Ethnicity[1]	White[2]	xx (xx%)	xx (xx%)	xx (xx%)
		Mixed/multiple[3]	xx (xx%)	xx (xx%)	xx (xx%)

	Asian/Asian British[4]	xx (xx%)	xx (xx%)	xx (xx%)
	Black/African/Caribbean/Black British[5]	xx (xx%)	xx (xx%)	xx (xx%)
	Other[6]	xx (xx%)	xx (xx%)	xx (xx%)
	Prefer not to say	xx (xx%)	xx (xx%)	xx (xx%)
Highest education qualification	CSE/GCSE/O Level	xx (xx%)	xx (xx%)	xx (xx%)
	No qualifications	xx (xx%)	xx (xx%)	xx (xx%)
	GCSEs or equivalent	xx (xx%)	xx (xx%)	xx (xx%)
	A levels or equivalent	xx (xx%)	xx (xx%)	xx (xx%)
	NVQ or equivalent	xx (xx%)	xx (xx%)	xx (xx%)
	Degree or equivalent	xx (xx%)	xx (xx%)	xx (xx%)
	Apprenticeship	xx (xx%)	xx (xx%)	xx (xx%)
	Master's Degree	xx (xx%)	xx (xx%)	xx (xx%)
	PhD	xx (xx%)	xx (xx%)	xx (xx%)
	Other	xx (xx%)	xx (xx%)	xx (xx%)
Employment status	Employed full-time	xx (xx%)	xx (xx%)	xx (xx%)
	Employed part-time	xx (xx%)	xx (xx%)	xx (xx%)
	Self-employed	xx (xx%)	xx (xx%)	xx (xx%)
	Retired	xx (xx%)	xx (xx%)	xx (xx%)
	Looking after family or home	xx (xx%)	xx (xx%)	xx (xx%)
	Student (full or part-time)	xx (xx%)	xx (xx%)	xx (xx%)
	Voluntary worker	xx (xx%)	xx (xx%)	xx (xx%)
	Not employed and seeking work	xx (xx%)	xx (xx%)	xx (xx%)
	Not employed and not seeking work because of ill health	xx (xx%)	xx (xx%)	xx (xx%)
	Not employed and not seeking work for some other reason	xx (xx%)	xx (xx%)	xx (xx%)
	Other	xx (xx%)	xx (xx%)	xx (xx%)
Approximate gross (before tax) household income	£0 - £300 per week	xx (xx%)	xx (xx%)	xx (xx%)
	£301 - £490 per week	xx (xx%)	xx (xx%)	xx (xx%)
	£491 - £740 per week	xx (xx%)	xx (xx%)	xx (xx%)
	£741 - £1,111 per week	xx (xx%)	xx (xx%)	xx (xx%)
	£1,112 or more per week	xx (xx%)	xx (xx%)	xx (xx%)
	Did not want to disclose	xx (xx%)	xx (xx%)	xx (xx%)

**Health risk behaviours**	Ever smoked	Yes	xx (xx%)	xx (xx%)	xx (xx%)
		No	xx (xx%)	xx (xx%)	xx (xx%)
	If yes, smoking status	Current smoker	xx (xx%)	xx (xx%)	xx (xx%)
		Previous smoker	xx (xx%)	xx (xx%)	xx (xx%)
	If previous, stopped	Within last year	xx (xx%)	xx (xx%)	xx (xx%)
		1 to 5 years ago	xx (xx%)	xx (xx%)	xx (xx%)
		5+ years ago	xx (xx%)	xx (xx%)	xx (xx%)
	If yes, product type	Packet cigarettes	xx (xx%)	xx (xx%)	xx (xx%)
		Hand-rolled cigarettes/tobacco	xx (xx%)	xx (xx%)	xx (xx%)
		Cigars	xx (xx%)	xx (xx%)	xx (xx%)
		Pipe	xx (xx%)	xx (xx%)	xx (xx%)
	If yes, number of cigarettes per day	1-10	xx (xx%)	xx (xx%)	xx (xx%)
		10-20	xx (xx%)	xx (xx%)	xx (xx%)
		20+	xx (xx%)	xx (xx%)	xx (xx%)
	Portions of fruit and vegetables per day	0	xx (xx%)	xx (xx%)	xx (xx%)
		1	xx (xx%)	xx (xx%)	xx (xx%)
		2	xx (xx%)	xx (xx%)	xx (xx%)
		3	xx (xx%)	xx (xx%)	xx (xx%)
		5+	xx (xx%)	xx (xx%)	xx (xx%)
	Consume carbonated drinks	Yes	xx (xx%)	xx (xx%)	xx (xx%)
		No	xx (xx%)	xx (xx%)	xx (xx%)
	If yes, number of times per day	Less than 1 time per day	xx (xx%)	xx (xx%)	xx (xx%)
		1 time per day	xx (xx%)	xx (xx%)	xx (xx%)
		2 times per day	xx (xx%)	xx (xx%)	xx (xx%)
		3 times per day	xx (xx%)	xx (xx%)	xx (xx%)
		4 times per day	xx (xx%)	xx (xx%)	xx (xx%)
		5+ times per day	xx (xx%)	xx (xx%)	xx (xx%)
**Mental health status**	Condition	Currently well	xx (xx%)	xx (xx%)	xx (xx%)
		Currently unwell	xx (xx%)	xx (xx%)	xx (xx%)
		Unsure	xx (xx%)	xx (xx%)	xx (xx%)
**Accelerometer**	MVPA	Mean (SD)	xx (xx)	xx (xx)	xx (xx)
		Median (IQR)	xx (xx)	xx (xx)	xx (xx)
		Min, Max	xx, xx	xx, xx	xx, xx

	Vigorous activity per day	Mean (SD)	xx (xx)	xx (xx)	xx (xx)
		Median (IQR)	xx (xx)	xx (xx)	xx (xx)
		Min, Max	xx, xx	xx, xx	xx, xx
	Moderate activity per day	Mean (SD)	xx (xx)	xx (xx)	xx (xx)
		Median (IQR)	xx (xx)	xx (xx)	xx (xx)
		Min, Max	xx, xx	xx, xx	xx, xx
	Light activity per day	Mean (SD)	xx (xx)	xx (xx)	xx (xx)
		Median (IQR)	xx (xx)	xx (xx)	xx (xx)
		Min, Max	xx, xx	xx, xx	xx, xx
	Inactive minutes per day	Mean (SD)	xx (xx)	xx (xx)	xx (xx)
		Median (IQR)	xx (xx)	xx (xx)	xx (xx)
		Min, Max	xx, xx	xx, xx	xx, xx
	Mean acceleration	Mean (SD)	xx (xx)	xx (xx)	xx (xx)
	Intensity gradient	Median (IQR)	xx (xx)	xx (xx)	xx (xx)
		Min, Max	xx, xx	xx, xx	xx, xx
**SIMPAQ**	Average hours in bed per night	Mean (SD)	xx (xx)	xx (xx)	xx (xx)
		Median (IQR)	xx (xx)	xx (xx)	xx (xx)
		Min, Max	xx, xx	xx, xx	xx, xx
	Average hours sedentary per day	Mean (SD)	xx (xx)	xx (xx)	xx (xx)
		Median (IQR)	xx (xx)	xx (xx)	xx (xx)
		Min, Max	xx, xx	xx, xx	xx, xx
	Average hours walking per day	Mean (SD)	xx (xx)	xx (xx)	xx (xx)
		Median (IQR)	xx (xx)	xx (xx)	xx (xx)
		Min, Max	xx, xx	xx, xx	xx, xx
	Average hours exercise per day	Mean (SD)	xx (xx)	xx (xx)	xx (xx)
		Median (IQR)	xx (xx)	xx (xx)	xx (xx)
		Min, Max	xx, xx	xx, xx	xx, xx
	Average hours other activities per day	Mean (SD)	xx (xx)	xx (xx)	xx (xx)
		Median (IQR)	xx (xx)	xx (xx)	xx (xx)
		Min, Max	xx, xx	xx, xx	xx, xx
**PHQ-9**	Total score	Mean (SD)	xx (xx)	xx (xx)	xx (xx)
		Median (IQR)	xx (xx)	xx (xx)	xx (xx)
		Min, Max	xx, xx	xx, xx	xx, xx

**GAD-7**	Total score	Mean (SD)	xx (xx)	xx (xx)	xx (xx)
		Median (IQR)	xx (xx)	xx (xx)	xx (xx)
		Min, Max	xx, xx	xx, xx	xx, xx
**EQ-5D**	EQ-5D-5L	Mean (SD)	xx (xx)	xx (xx)	xx (xx)
		Median (IQR)	xx (xx)	xx (xx)	xx (xx)
		Min, Max	xx, xx	xx, xx	xx, xx
	VAS score	Mean (SD)	xx (xx)	xx (xx)	xx (xx)
		Median (IQR)	xx (xx)	xx (xx)	xx (xx)
		Min, Max	xx, xx	xx, xx	xx, xx
**ReQoL**	ReQoL-10 index score	Mean (SD)	xx (xx)	xx (xx)	xx (xx)
		Median (IQR)	xx (xx)	xx (xx)	xx (xx)
		Min, Max	xx, xx	xx, xx	xx, xx
	ReQoL-20 overall index score	Mean (SD)	xx (xx)	xx (xx)	xx (xx)
		Median (IQR)	xx (xx)	xx (xx)	xx (xx)
		Min, Max	xx, xx	xx, xx	xx, xx
**UCLA 3-item**	Total score	Mean (SD)	xx (xx)	xx (xx)	xx (xx)
		Median (IQR)	xx (xx)	xx (xx)	xx (xx)
		Min, Max	xx, xx	xx, xx	xx, xx
**Predictive behaviour questionnaire**	BPNES Autonomy score	Mean (SD)	xx (xx)	xx (xx)	xx (xx)
		Median (IQR)	xx (xx)	xx (xx)	xx (xx)
		Min, Max	xx, xx	xx, xx	xx, xx
	BPNES Competence score	Mean (SD)	xx (xx)	xx (xx)	xx (xx)
		Median (IQR)	xx (xx)	xx (xx)	xx (xx)
		Min, Max	xx, xx	xx, xx	xx, xx
	BPNES Relatedness score	Mean (SD)	xx (xx)	xx (xx)	xx (xx)
		Median (IQR)	xx (xx)	xx (xx)	xx (xx)
		Min, Max	xx, xx	xx, xx	xx, xx
	TPB Instrumental Attitude score	Mean (SD)	xx (xx)	xx (xx)	xx (xx)
		Median (IQR)	xx (xx)	xx (xx)	xx (xx)
		Min, Max	xx, xx	xx, xx	xx, xx
	TPB Experiential Attitude score	Mean (SD)	xx (xx)	xx (xx)	xx (xx)
		Median (IQR)	xx (xx)	xx (xx)	xx (xx)
		Min, Max	xx, xx	xx, xx	xx, xx

TPB Injunctive Norm score	Mean (SD)	xx (xx)	xx (xx)	xx (xx)
	Median (IQR)	xx (xx)	xx (xx)	xx (xx)
	Min, Max	xx, xx	xx, xx	xx, xx
TPB Descriptive Norm score	Mean (SD)	xx (xx)	xx (xx)	xx (xx)
	Median (IQR)	xx (xx)	xx (xx)	xx (xx)
	Min, Max	xx, xx	xx, xx	xx, xx
TPB Autonomy score	Mean (SD)	xx (xx)	xx (xx)	xx (xx)
	Median (IQR)	xx (xx)	xx (xx)	xx (xx)
	Min, Max	xx, xx	xx, xx	xx, xx
TPB Capacity score	Mean (SD)	xx (xx)	xx (xx)	xx (xx)
	Median (IQR)	xx (xx)	xx (xx)	xx (xx)
	Min, Max	xx, xx	xx, xx	xx, xx
TPB Intention score	Mean (SD)	xx (xx)	xx (xx)	xx (xx)
	Median (IQR)	xx (xx)	xx (xx)	xx (xx)
	Min, Max	xx, xx	xx, xx	xx, xx

[1] The main ethnic groups could be collapsed depending on the observed distribution

[2] English/Welsh/Scottish/Northern Irish/British, Irish, Gypsy or Irish Traveller, Roma, and Any other

[3] White and Black Caribbean, White and Black African, White and Asian, and Any other

[4] Indian, Pakistani, Bangladeshi, Chinese, and Any other

[5] African, Caribbean, and Any other Black/African/Caribbean/Black British background

[6] Arab, and Any other ethnic groups

Table: Summary of SPACES intervention

Variable	Scoring	Intervention (n=xx)
**Number of SPACES intervention groups**	n	xx
**Group session size**	Mean (SD)	xx (xx)
	Median (IQR)	xx (xx)
	Min, Max	xx, xx
**Type of physical activity**	Indoor circuit style	xx (xx%)
	Walking	xx (xx%)
	Community session	xx (xx%)
	Playing sport	xx (xx%)
	Other	xx (xx%)
**Group sessions attended (out of a possible 18)**	All 6 sessions	xx (xx%)
	At least 9 sessions	xx (xx%)
	At least 1 session	xx (xx%)
	0 sessions	xx (xx%)
**Individual meetings attended (out of a possible 6)**	All 6 sessions	xx (xx%)
	At least 3 sessions	xx (xx%)
	At least 1 session	xx (xx%)
	0 sessions	xx (xx%)
**Accompanied in at least one group session**	Yes	xx (xx%)
	No	xx (xx%)
	Never attended	xx (xx%)
**If accompanied, how many sessions?**	Mean (SD)	xx (xx)
	Median (IQR)	xx (xx)
	Min, Max	xx, xx
**Took part in physical activity**	All 18 sessions	xx (xx%)
	At least 9 sessions	xx (xx%)
	At least 1 session	xx (xx%)
	0 sessions	xx (xx%)
**Took up offer of pedometer**	Yes	xx (xx%)
	No	xx (xx%)

**Engaged in community sessions outside of SPACES**	Yes	xx (xx%)
	No	xx (xx%)

Figure: *Box plot showing number of participants attending a session, by session number*

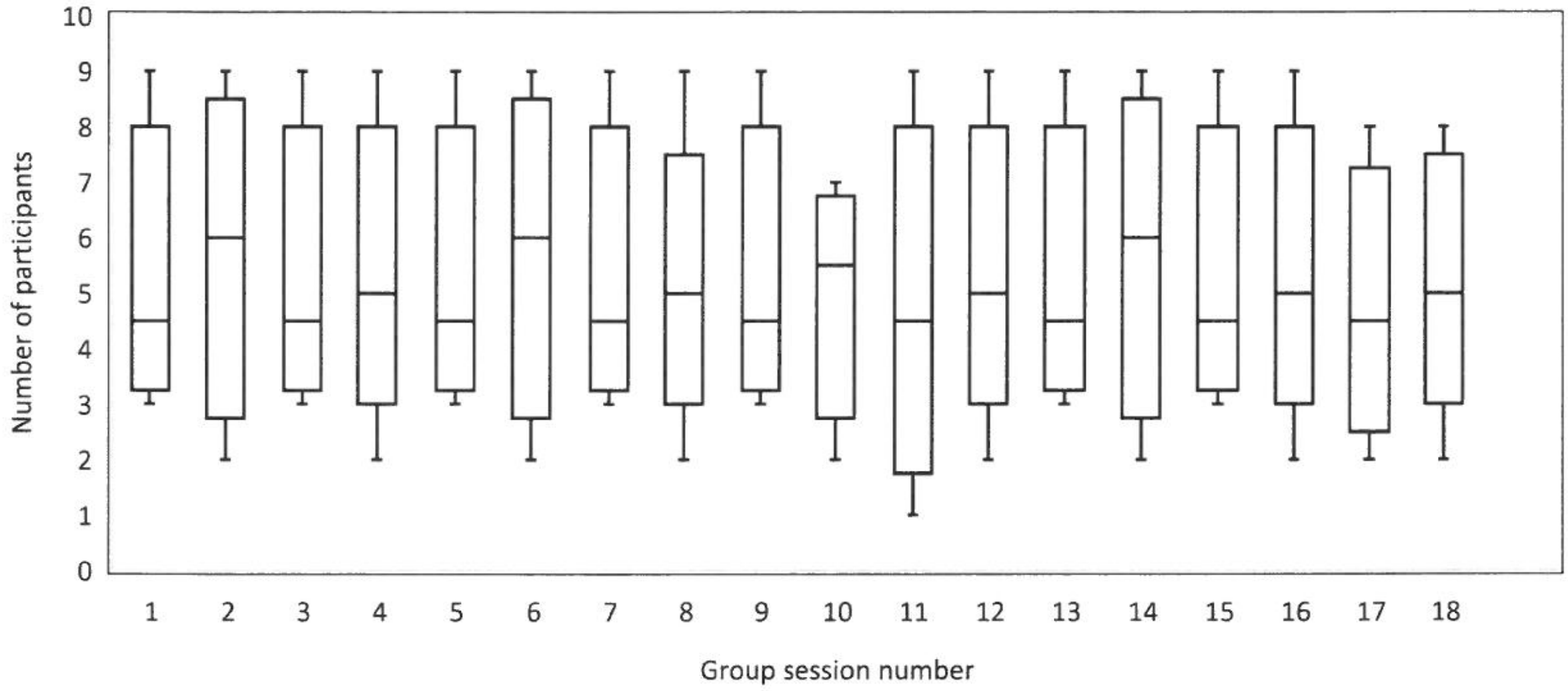

Table: *Reasons for discontinuation and withdrawal*

Variable	Scoring	Control (n=xx)	Intervention (n=xx)	Total (n=xx)
**Participant discontinuation**	Participant withdrew consent	xx (xx%)	xx (xx%)	xx (xx%)
	Lost to follow-up	xx (xx%)	xx (xx%)	xx (xx%)
	Investigator decision	xx (xx%)	xx (xx%)	xx (xx%)
**Participant reason for withdrawal**	n	xx	xx	xx
	No longer wants to participate in a randomised trial	xx (xx%)	xx (xx%)	xx (xx%)
	Feels too ill	xx (xx%)	xx (xx%)	xx (xx%)
	No longer interested	xx (xx%)	xx (xx%)	xx (xx%)
	Other	xx (xx%)	xx (xx%)	xx (xx%)

Table: *Reasons for intervention non-attendance and withdrawal*

Variable	Scoring	Intervention (n=xx)

**Reason for non-attendance**	n	xxx
	Participant illness	xx (xx%)
	Participant holiday	xx (xx%)
	Unable to contact participant	xx (xx%)
	Unknown reason	xx (xx%)
	Other	xx (xx%)
**Participant decision to withdraw from intervention**	n	xx
	Too time consuming / too much effort	xx (xx%)
	Too far to travel	xx (xx%)
	Did not find intervention worthwhile	xx (xx%)
	Didn't like being in a group	xx (xx%)
	Doesn't like doing physical activity	xx (xx%)
	Prefers not to say	xx (xx%)
	Other	xx (xx%)
	Not stated	xx (xx%)
**Investigator decision to withdraw**	n	xx
	Loss of capacity to consent	xx (xx%)
	Physical illness/acquired medical condition that prevents engagement	xx (xx%)
	Major escalation of mental health symptoms preventing engagement	xx (xx%)
	Other	xx (xx%)

**Variable**	**Scoring**	**Intervention attendance**		
		**≥9 group and ≥3 individual sessions (n=xx)**	**<9 group or <3 individual sessions (n=xx)**	**Total (N=xx)**
**Age (years)**	Mean (SD)	xx (xx)	xx (xx)	xx (xx)
	Median (IQR)	xx (xx)	xx (xx)	xx (xx)
	Min, Max	xx, xx	xx, xx	xx, xx
**Sex at birth**	Male	xx (xx%)	xx (xx%)	xx (xx%)
	Female	xx (xx%)	xx (xx%)	xx (xx%)

**Mental health status**	Currently well	xx (xx%)	xx (xx%)	xx (xx%)
	Currently unwell	xx (xx%)	xx (xx%)	xx (xx%)
	Unsure	xx (xx%)	xx (xx%)	xx (xx%)

Table: *Participant characteristics by intervention attendance status for participants randomised to the intervention arm*

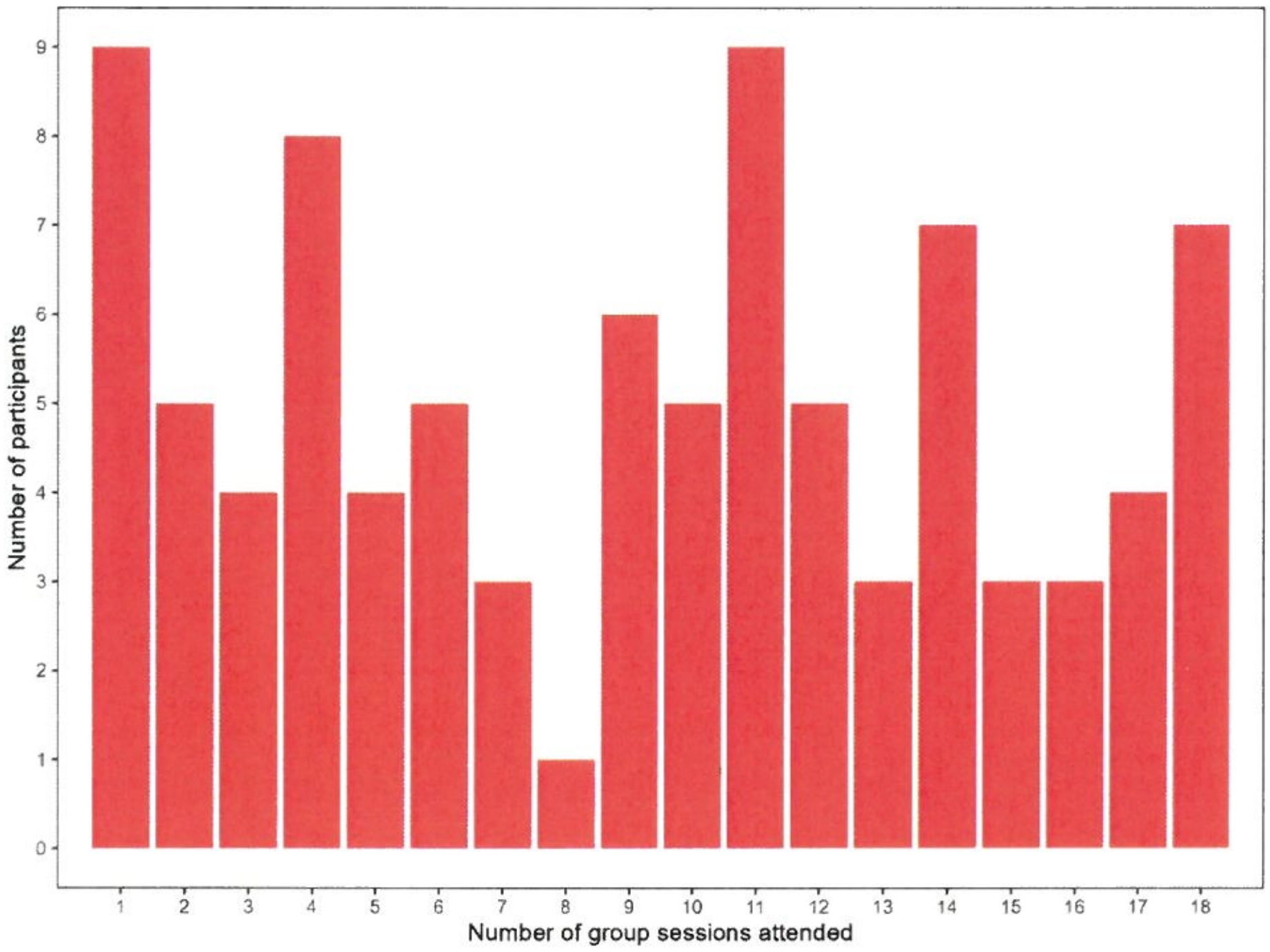

Figure: *Histogram of number of group sessions that participants attended*

Table: *Accelerometer-assessed outcomes per day (averaged over 10 days) at 3-, 6- and 12-months follow-up*

	Control (n=xx)		Intervention (n=xx)		Unadjusted mean difference (95% CI)	Adjusted mean difference (95% CI)
	n	Mean (SD)	n	Mean (SD)		
**3 months**						
**MVPA per day**	xx	xx (xx)	xx	xx (xx)	xx (xx to xx)	xx (xx to xx)

**Sedentary activity (inactive minutes)**	xx	xx (xx)	xx	xx (xx)	xx (xx to xx)	xx (xx to xx)
**...***						
**6 months**						
**MVPA per day**	xx	xx (xx)	xx	xx (xx)	xx (xx to xx)	xx (xx to xx)
**Sedentary activity (inactive minutes)**	xx	xx (xx)	xx	xx (xx)	xx (xx to xx)	xx (xx to xx)
**...***						
**12 months**						
**MVPA per day**	xx	xx (xx)	xx	xx (xx)	xx (xx to xx)	xx (xx to xx)
**Sedentary activity (inactive minutes)**	xx	xx (xx)	xx	xx (xx)	xx (xx to xx)	xx (xx to xx)
**...***						

**Vigorous activity, moderate activity, light activity, mean acceleration, intensity gradient*

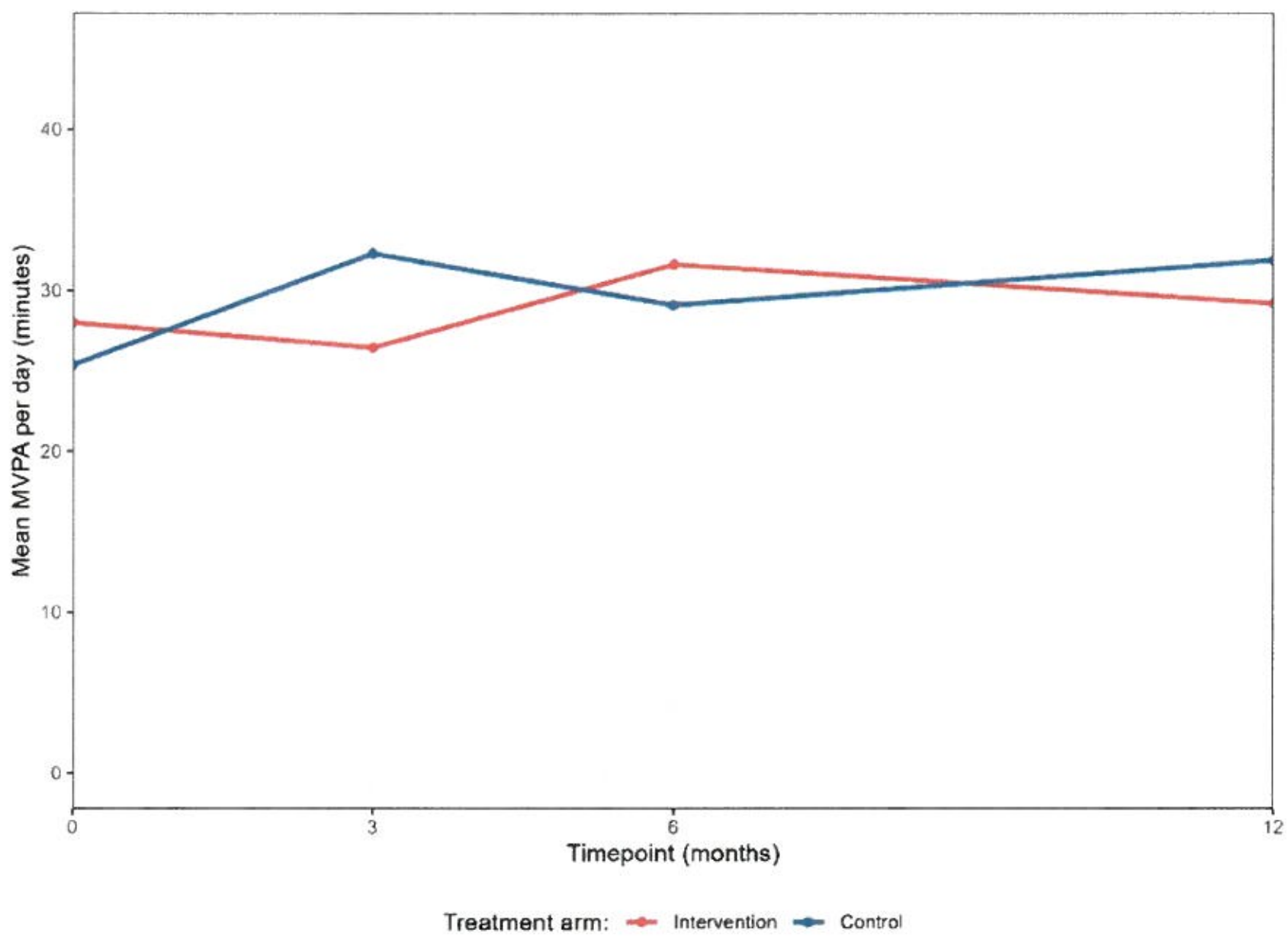

Figure: *Line graph of mean minutes of MVPA by treatment arm*

Figure: *Boxplots summarising minutes of MVPA at each time point*

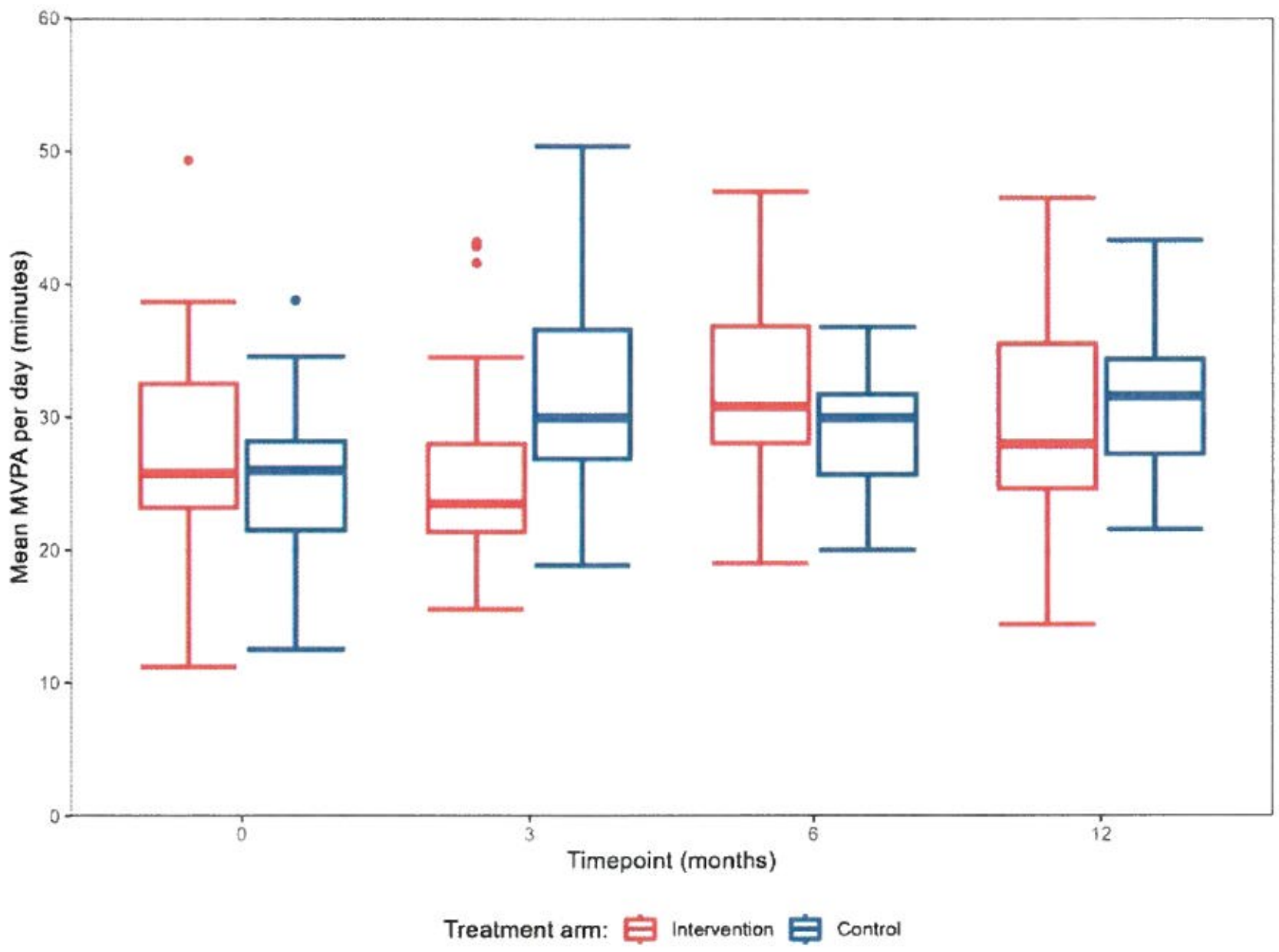

Table: *Validity of accelerometers*

Variable	Scoring	Control (n=xx)	Intervention (n=xx)	All (n=xx)
**Baseline**	Valid wear time[1]	xx (xx%)	xx (xx%)	xx (xx%)
	Did not have valid wear time (but had some data)	xx (xx%)	xx (xx%)	xx (xx%)
	No data	xx (xx%)	xx (xx%)	xx (xx%)
**3-month**	Valid wear time[1]	xx (xx%)	xx (xx%)	xx (xx%)
	Did not have valid wear time (but had some data)	xx (xx%)	xx (xx%)	xx (xx%)
	No data	xx (xx%)	xx (xx%)	xx (xx%)
**6-month**	Valid wear time[1]	xx (xx%)	xx (xx%)	xx (xx%)
	Did not have valid wear time (but had some data)	xx (xx%)	xx (xx%)	xx (xx%)
	No data	xx (xx%)	xx (xx%)	xx (xx%)

[1]. A minimum of 16 -hour a day wear time for at least 3 days over the 10-day collection period.

Table: *Body mass index (BMI) at at follow-up by treatment arm*

	Control (n=xx)		Intervention (n=xx)		Mean difference
	**n**	**Mean (SD)**	**n**	**Mean (SD)**	**(95% CI)**
**Baseline**	xx	xx(xx)	xx	xx (xx)	xx (xx to xx)
**3-month**	xx	xx (xx)	xx	xx (xx)	xx (xx to xx)
**6-month**	xx	xx (xx)	xx	xx (xx)	xx (xx to xx)
**12-month**	xx	xx (xx)	xx	xx (xx)	xx (xx to xx)

Table: *Questionnaires at baseline and at follow-up by treatment arm*

			Control (n=xx)		Intervention (n=xx)		Mean difference (95% CI)
			**n**	**Mean (SD)**	**n**	**Mean (SD)**	
**Baseline**	PHQ-9		xx	xx (xx)	xx	xx (xx)	xx (xx to xx)
	GAD-7		xx	xx (xx)	xx	xx (xx)	xx (xx to xx)
	SIMPAQ	Bed	xx	xx (xx)	xx	xx (xx)	xx (xx to xx)
		Sedentary	xx	xx (xx)	xx	xx (xx)	xx (xx to xx)
		Walking	xx	xx (xx)	xx	xx (xx)	xx (xx to xx)
		Exercise	xx	xx (xx)	xx	xx (xx)	xx (xx to xx)
		Other	xx	xx (xx)	xx	xx (xx)	xx (xx to xx)
	EQ5D	EQ-5D-5L	xx	xx (xx)	xx	xx (xx)	xx (xx to xx)
		VAS	xx	xx (xx)	xx	xx (xx)	xx (xx to xx)
	ReQoL	ReQoL-10	xx	xx (xx)	xx	xx (xx)	xx (xx to xx)
		ReQoL-20	xx	xx (xx)	xx	xx (xx)	xx (xx to xx)
	UCLA 3-item		xx	xx (xx)	xx	xx (xx)	xx (xx to xx)
	PBQ BPNES	Autonomy	xx	xx (xx)	xx	xx (xx)	xx (xx to xx)
		Competence	xx	xx (xx)	xx	xx (xx)	xx (xx to xx)
		Relatedness	xx	xx (xx)	xx	xx (xx)	xx (xx to xx)
	PBQ TPB	Instrumental Attitude	xx	xx (xx)	xx	xx (xx)	xx (xx to xx)
		Experiential Attitude	xx	xx (xx)	xx	xx (xx)	xx (xx to xx)
		Injunctive Norm	xx	xx (xx)	xx	xx (xx)	xx (xx to xx)

		Descriptive Norm	xx	xx (xx)	xx	xx (xx)	xx (xx to xx)
		Autonomy	xx	xx (xx)	xx	xx (xx)	xx (xx to xx)
		Capacity	xx	xx (xx)	xx	xx (xx)	xx (xx to xx)
		Intention	xx	xx (xx)	xx	xx (xx)	xx (xx to xx)
**3-month**	PHQ-9		xx	xx (xx)	xx	xx (xx)	xx (xx to xx)
	GAD-7		xx	xx (xx)	xx	xx (xx)	xx (xx to xx)
	SIMPAQ	Bed	xx	xx (xx)	xx	xx (xx)	xx (xx to xx)
		Sedentary	xx	xx (xx)	xx	xx (xx)	xx (xx to xx)
		Walking	xx	xx (xx)	xx	xx (xx)	xx (xx to xx)
		Exercise	xx	xx (xx)	xx	xx (xx)	xx (xx to xx)
		Other	xx	xx (xx)	xx	xx (xx)	xx (xx to xx)
	EQ5D	EQ-5D-5L	xx	xx (xx)	xx	xx (xx)	xx (xx to xx)
		VAS	xx	xx (xx)	xx	xx (xx)	xx (xx to xx)
	ReQoL	ReQoL-10	xx	xx (xx)	xx	xx (xx)	xx (xx to xx)
		ReQoL-20	xx	xx (xx)	xx	xx (xx)	xx (xx to xx)
	UCLA 3-item		xx	xx (xx)	xx	xx (xx)	xx (xx to xx)
	PBQ BPNES	Autonomy	xx	xx (xx)	xx	xx (xx)	xx (xx to xx)
		Competence	xx	xx (xx)	xx	xx (xx)	xx (xx to xx)
		Relatedness	xx	xx (xx)	xx	xx (xx)	xx (xx to xx)
	PBQ TPB	Instrumental Attitude	xx	xx (xx)	xx	xx (xx)	xx (xx to xx)
		Experiential Attitude	xx	xx (xx)	xx	xx (xx)	xx (xx to xx)
		Injunctive Norm	xx	xx (xx)	xx	xx (xx)	xx (xx to xx)
		Descriptive Norm	xx	xx (xx)	xx	xx (xx)	xx (xx to xx)
		Autonomy	xx	xx (xx)	xx	xx (xx)	xx (xx to xx)
		Capacity	xx	xx (xx)	xx	xx (xx)	xx (xx to xx)
		Intention	xx	xx (xx)	xx	xx (xx)	xx (xx to xx)
**6-month**	PHQ-9		xx	xx (xx)	xx	xx (xx)	xx (xx to xx)
	GAD-7		xx	xx (xx)	xx	xx (xx)	xx (xx to xx)

	SIMPAQ	Bed	xx	xx (xx)	xx	xx (xx)	xx (xx to xx)
		Sedentary	xx	xx (xx)	xx	xx (xx)	xx (xx to xx)
		Walking	xx	xx (xx)	xx	xx (xx)	xx (xx to xx)
		Exercise	xx	xx (xx)	xx	xx (xx)	xx (xx to xx)
		Other	xx	xx (xx)	xx	xx (xx)	xx (xx to xx)
	EQ5D	EQ-5D-5L	xx	xx (xx)	xx	xx (xx)	xx (xx to xx)
		VAS	xx	xx (xx)	xx	xx (xx)	xx (xx to xx)
	ReQoL	ReQoL-10	xx	xx (xx)	xx	xx (xx)	xx (xx to xx)
		ReQoL-20	xx	xx (xx)	xx	xx (xx)	xx (xx to xx)
	UCLA 3-item		xx	xx (xx)	xx	xx (xx)	xx (xx to xx)
	PBQ BPNES	Autonomy	xx	xx (xx)	xx	xx (xx)	xx (xx to xx)
		Competence	xx	xx (xx)	xx	xx (xx)	xx (xx to xx)
		Relatedness	xx	xx (xx)	xx	xx (xx)	xx (xx to xx)
	PBQ TPB	Instrumental Attitude	xx	xx (xx)	xx	xx (xx)	xx (xx to xx)
		Experiential Attitude	xx	xx (xx)	xx	xx (xx)	xx (xx to xx)
		Injunctive Norm	xx	xx (xx)	xx	xx (xx)	xx (xx to xx)
		Descriptive Norm	xx	xx (xx)	xx	xx (xx)	xx (xx to xx)
		Autonomy	xx	xx (xx)	xx	xx (xx)	xx (xx to xx)
		Capacity	xx	xx (xx)	xx	xx (xx)	xx (xx to xx)
		Intention	xx	xx (xx)	xx	xx (xx)	xx (xx to xx)
**12-month**	PHQ-9		xx	xx (xx)	xx	xx (xx)	xx (xx to xx)
	GAD-7		xx	xx (xx)	xx	xx (xx)	xx (xx to xx)
	SIMPAQ	Bed	xx	xx (xx)	xx	xx (xx)	xx (xx to xx)
		Sedentary	xx	xx (xx)	xx	xx (xx)	xx (xx to xx)
		Walking	xx	xx (xx)	xx	xx (xx)	xx (xx to xx)
		Exercise	xx	xx (xx)	xx	xx (xx)	xx (xx to xx)
		Other	xx	xx (xx)	xx	xx (xx)	xx (xx to xx)
	EQ5D	EQ-5D-5L	xx	xx (xx)	xx	xx (xx)	xx (xx to xx)
		VAS	xx	xx (xx)	xx	xx (xx)	xx (xx to xx)

ReQoL	ReQoL-10	xx	xx (xx)	xx	xx (xx)	xx (xx to xx)
	ReQoL-20	xx	xx (xx)	xx	xx (xx)	xx (xx to xx)
UCLA 3-item		xx	xx (xx)	xx	xx (xx)	xx (xx to xx)
PBQ BPNES	Autonomy	xx	xx (xx)	xx	xx (xx)	xx (xx to xx)
	Competence	xx	xx (xx)	xx	xx (xx)	xx (xx to xx)
	Relatedness	xx	xx (xx)	xx	xx (xx)	xx (xx to xx)
PBQ TPB	Instrumental Attitude	xx	xx (xx)	xx	xx (xx)	xx (xx to xx)
	Experiential Attitude	xx	xx (xx)	xx	xx (xx)	xx (xx to xx)
	Injunctive Norm	xx	xx (xx)	xx	xx (xx)	xx (xx to xx)
	Descriptive Norm	xx	xx (xx)	xx	xx (xx)	xx (xx to xx)
	Autonomy	xx	xx (xx)	xx	xx (xx)	xx (xx to xx)
	Capacity	xx	xx (xx)	xx	xx (xx)	xx (xx to xx)
	Intention	xx	xx (xx)	xx	xx (xx)	xx (xx to xx)

Table: *Summary of adverse and serious adverse events (AEs and SAEs) experienced by treatment arm*

Variable	Scoring	Control (n=xx)	Intervention (n=xx)	All (N=xx)
**Number of AEs**		xx (xx%)	xx (xx%)	xx (xx%)
**Number of AEs related to intervention**		xx (xx%)	xx (xx%)	xx (xx%)
**Number of AEs related to trial**		xx (xx%)	xx (xx%)	xx (xx%)
**Participants with ≥ 1 AEs**		xx (xx%)	xx (xx%)	xx (xx%)
**Type of AEs**				
	Muscle soreness	xx (xx%)	xx (xx%)	xx (xx%)
	Substantial increase in pre-existing pain	xx (xx%)	xx (xx%)	xx (xx%)
	Development of musculoskeletal pain	xx (xx%)	xx (xx%)	xx (xx%)
	Injuries sustained as a result of an accident incurred	xx (xx%)	xx (xx%)	xx (xx%)
	...	...	...	...
**Number of SAEs**		xx (xx%)	xx (xx%)	xx (xx%)
**Number of SAEs related to intervention**		xx (xx%)	xx (xx%)	xx (xx%)
**Number of SAEs related to trial**		xx (xx%)	xx (xx%)	xx (xx%)
**Participants with ≥ 1 SAEs**		xx (xx%)	xx (xx%)	xx (xx%)
**Type of SAEs**	...	...	...	...

Appendix 2 Questionnaire Outcome Summary Table

Name	No. of items	Score range	Description	Interpretation of score
EQ-5D-5L value index	5	(-0.224, 1)	Measure of health status. 5 domains include mobility, self-care, usual activities, pain/discomfort, and anxiety/depression.	A score of zero means death, 1 is full health, negative score is a state worse than death.
EQ-5D-5L VAS	1	(0, 100)	Measure of health status.	A score of zero means worst health and 100 means best health.
GAD-7	7	0-21	Measures severity of generalised anxiety disorder.	Higher score indicates increasing severity of anxiety; no (0 to 4), mild (5 to 9), moderate (10 to 14), and severe anxiety (15 to 21).
PHQ-9	9	0-27	Measures severity of depression.	Higher score indicates increasing severity of depression; none (0 to 4), mild depression (5 to 9), moderate depression (10 to 14), moderately severe depression (15 to 19), and severe depression (20 to 27)
ReQoL	20	0-80	Assess quality of life for people with different mental health conditions.	Higher score indicates better quality of life.
SIMPAQ	5	NA (domains should sum to 24 hours)	Measure of physical activity and sedentary behaviour.	NA
UCLA 3-item	3	3-9	Measures loneliness. 3 dimensions: companionship, feeling left out, isolation.	Higher score indicates more lonely.
Predictive behaviour questionnaire	26	NA (no overall score)	Predict participant physical activity. BPNES and TPB split into constructs/sub-constructs in which mean scores are calculated.	Higher scores are associated with more favourable attributes.
