# Supplementary material for: Supporting physical activity through co-production in people with severe mental illness (SPACES): study protocol for a pragmatic, parallel-group randomised controlled trial
Source: BMJ Open. 2026 Oct 8;16(10):e115528. doi: 10.1136/bmjopen-2025-115528 (PMC13656100; doi:10.1136/bmjopen-2025-115528)
Supplement: Supplementary file 2 [file bmjopen-2025-115528.reviewer_comments.pdf]

PEER REVIEW HISTORY

BMJ Open publishes all reviews undertaken for accepted manuscripts. Reviewers are asked to complete a checklist review form and are provided with free text boxes to elaborate on their assessment. These free text comments are reproduced below.

ARTICLE DETAILS

Title (Provisional)

Supporting physical activity through co-production in people with severe mental illness (SPACES): study protocol for a pragmatic parallel group randomised controlled trial

Authors

Burke, Trys; Hodgson, Philip; Jones, Gareth; Beeken, Rebecca J; Bowes, Eleanor; Cooper, Cindy; Copeland, Robert; Dawson, Sam; Faires, Matthew; Guy, Zoe; Hill, Andrew; Hillison, Emily; Horspool, Michelle; Li, Jinshuo; Machaczek, Katarzyna Karolina; Marks, Freya; Parrott, Steve; Quirk, Helen; Rombach, Ines; Stubbs, B; Tew, Garry; Traviss-Turner, Gemma; Turton, Emily; Walters, Stephen J; Weich, Scott; Wildbore, Ellie; Peckham, Emily Jane

---

VERSION 1 - REVIEW

**Reviewer**	**1**
**Name**	**Kilonzo, Mary**
**Affiliation**	**University of Aberdeen, Health Economics Research Unit**
**Date**	**20-Feb-2026**
**COI**	**None**

The key elements of the health economic analysis have been addressed. It would have been useful for the HE to have addressed how missing data will be handled.

**Reviewer**	**2**
**Name**	**Ren, Zhanbing**
**Affiliation**	**Shenzhen University, Physical Education**
**Date**	**21-Apr-2026**
**COI**	**None**

While the aim of the SPACES trial is commendable and the co-production approach to intervention development is a notable strength, several methodological and conceptual issues in the protocol warrant careful consideration prior to publication.

Page 9, Lines 38–44, the authors acknowledge substantial variation in usual care across sites and appropriately include site as a random effect in the statistical model. However, the Discussion section does not engage with this critical source of confounding in sufficient depth. Given that the treatment as usual condition may encompass widely differing levels of existing physical activity provision across NHS trusts, the observed intervention effect may be confounded by site-level service ecology. It would be important to address the anticipated heterogeneity of treatment effects and to outline a priori plans for exploring effect modification by usual care intensity.

Page 16, Lines 11–22, I have concerns regarding the clinical anchoring of the target difference. The 6-minute increase in moderate-to-vigorous physical activity per day is derived from a meta-analysis of all-cause mortality in the general population. While this provides a useful benchmark, its direct application as the minimal clinically important difference in a population with severe mental illness requires further justification. Individuals with SMI exhibit distinct physiological responses, lower baseline activity profiles, and iatrogenic metabolic burdens that may alter the relationship between activity increments and meaningful health outcomes. Moreover, the trial's primary endpoint is 6-month accelerometry-measured MVPA, not mortality. It remains unclear whether an increase of 6 minutes per day in this specific population translates into perceptible improvements in mental health symptoms, functional recovery, or quality of life， the very domains the intervention aims to influence. The protocol would benefit from a more transparent discussion of this inferential leap and, ideally, corroborative evidence from the feasibility study linking such a change to patient-reported outcomes.

Page 18, Lines 27–33, the authors correctly state that blinding of participants and clinicians is not feasible in this behavioural intervention trial. However, I am less convinced that blinding of all researchers is precluded. The protocol states that researchers will not be blinded, which raises a non-trivial risk of ascertainment bias, particularly for secondary outcomes that rely on participant self-report (e.g., PHQ-9, GAD-7, ReQoL). Even with an objective primary outcome, differential enthusiasm or inadvertent cueing by unblinded assessors could systematically inflate effect estimates for subjective measures. It would be prudent to clarify whether outcome assessors responsible for collecting questionnaire data will be blinded to treatment allocation, or, if this is truly impossible, to explicitly frame this as a key limitation and to describe planned sensitivity analyses to gauge the impact of potential bias.

Page 47, Lines 1–12, appears to presume that the trial design is equally powered and structured to establish both clinical and economic superiority. While the health economics analysis plan is detailed, the protocol does not adequately address the inherent

uncertainties in translating a 6-month activity change into a credible estimate of long-term cost-effectiveness for the NHS. Important cost drivers， such as informal caregiver time, participant out-of-pocket expenses, and the often-substantial training and staff turnover costs associated with implementing a new physical activity coordinator role， are not foregrounded. The conclusion that the study "will determine" cost-effectiveness may therefore overstate the design's capability. A more circumspect phrasing, such as "will evaluate the potential cost-effectiveness," would better reflect the exploratory nature of the economic component.

VERSION 1 - AUTHOR RESPONSE

Comment	Response
Reviewer: 1<br>Ms. Mary Kilonzo, University of Aberdeen<br>Comments to the Authors:<br>The key elements of the health economic analysis have been addressed. It would have been useful for the HE to have addressed how missing data will be handled.	We have described "missing cost and outcome data will be imputed using multiple imputation" in the second to last sentence under health economics analysis on p24. We have now moved it to the end of the second paragraph on p19 to make it clearer.
Reviewer: 2<br>Prof. Zhanbing Ren, Shenzhen University, Shenzhen University<br>Comments to the Authors:<br>While the aim of the SPACES trial is commendable and the co-production approach to intervention development is a notable strength, several methodological and conceptual issues in the protocol warrant careful consideration prior to publication.<br><br>Page 9, Lines 38–44, the authors acknowledge substantial variation in usual care across sites and appropriately include site as a random effect in the statistical model. However, the Discussion section does not engage with this critical source of	Thanks for this comment, we have added the following to the discussion:<br><br>'*A limitation of the study design is the potential heterogeneity of treatment as usual in the control group which may encompass widely differing levels of existing physical activity provision across NHS trusts. Although we are stratifying by site in the randomisation which should ensure a balance within sites and adjusting for site in the statistical analysis we cannot completely rule out that the observed intervention effect may be confounded by site-level service ecology.*'

confounding in sufficient depth. Given that the treatment as usual condition may encompass widely differing levels of existing physical activity provision across NHS trusts, the observed intervention effect may be confounded by site-level service ecology. It would be important to address the anticipated heterogeneity of treatment effects and to outline a priori plans for exploring effect modification by usual care intensity.	
Page 16, Lines 11–22, I have concerns regarding the clinical anchoring of the target difference. The 6-minute increase in moderate-to-vigorous physical activity per day is derived from a meta-analysis of all-cause mortality in the general population. While this provides a useful benchmark, its direct application as the minimal clinically important difference in a population with severe mental illness requires further justification. Individuals with SMI exhibit distinct physiological responses, lower baseline activity profiles, and iatrogenic metabolic burdens that may alter the relationship between activity increments and meaningful health outcomes. Moreover, the trial's primary endpoint is 6-month accelerometry-measured MVPA, not mortality. It remains unclear whether an increase of 6 minutes per day in this specific population translates into perceptible improvements in mental health symptoms, functional recovery, or quality of life, the very domains the intervention aims to influence. The protocol would benefit from a more transparent discussion of this inferential leap and, ideally, corroborative evidence from the feasibility study linking such a change to patient-reported outcomes.	We have added the following to the discussion<br><br>'*The trial is powered on a target difference of a 6-minute increase in moderate-to-vigorous physical activity per day and a potential link with mortality. However, a limitation of the sample size calculation is that it is unclear whether an increase of 6 minutes per day in this specific population translates into perceptible improvements in mental health symptoms (e.g. PHQ-9, GAD-7), functional recovery (ReQoL), or quality of life (EQ-5D), but we are measuring all of these as secondary outcomes).*'
Page 18, Lines 27–33, the authors correctly state that blinding of participants and clinicians is not feasible in this behavioural intervention trial. However, I am less convinced that blinding of all researchers is precluded. The protocol states that researchers will not be blinded, which raises a non-trivial risk of ascertainment bias, particularly for secondary outcomes that rely on participant self-report (e.g., PHQ-9, GAD-7, ReQoL). Even with an objective primary outcome, differential enthusiasm	Thank you for highlighting this important point. It is not possible to blind the researcher because the researcher who conducts the baseline interview and randomises the participant, does the randomisation whilst they are with the participant so that they can inform them of their allocation and what will

or inadvertent cueing by unblinded assessors could systematically inflate effect estimates for subjective measures. It would be prudent to clarify whether outcome assessors responsible for collecting questionnaire data will be blinded to treatment allocation, or, if this is truly impossible, to explicitly frame this as a key limitation and to describe planned sensitivity analyses to gauge the impact of potential bias	happen next. Thus, the researcher is not blinded. We have explained this in the protocol:<br><br>*'When the second baseline data is collected (i.e., when the accelerometer is returned after 10 days), the SPACES researcher will contact the central team to obtain the participant's randomisation assignment. Participants will then be informed by the researcher at the second baseline interview of their group allocation.'*<br><br>On consultation with people with lived experience, plus our experience of other studies in this population we have learnt that people prefer and are more likely to complete the follow up with someone they already know and trust. Therefore, where possible the same person who conducts the baseline interview conducts the follow up meaning that it is not possible to blind the researcher. We accept that this is a limitation and have added to the limitations section in the discussion.<br><br>*'It is not possible to blind researchers conducting the follow up interviews to allocation, this could introduce bias in the self-completion measures (PHQ-9, GAD-7 etc). However, our primary outcome is an objective measure and due*

	to this we have not planned any sensitivity analyses.'
Page 47, Lines 1–12, appears to presume that the trial design is equally powered and structured to establish both clinical and economic superiority. While the health economics analysis plan is detailed, the protocol does not adequately address the inherent uncertainties in translating a 6-month activity change into a credible estimate of long-term cost-effectiveness for the NHS. Important cost drivers, such as informal caregiver time, participant out-of-pocket expenses, and the often-substantial training and staff turnover costs associated with implementing a new physical activity coordinator role, are not foregrounded. The conclusion that the study "will determine" cost-effectiveness may therefore overstate the design's capability. A more circumspect phrasing, such as "will evaluate the potential cost-effectiveness," would better reflect the exploratory nature of the economic component.	Thank you for the comment. We agree that within a trial setting, the long-term cost-effectiveness for the NHS could not be determined definitively. The sample size was not estimated based on economic outcomes. We have now rephrased the statement as: '*and evaluate the potential cost effectiveness of the intervention for the NHS*' However, we would like to point out that we did not include informal caregiver time, participants out of pocket expenses because they are not costs from NHS perspective. We did not intend to evaluate the intervention from participants and their family perspective. We included training costs for the intervention in our data collection but neglected to describe it in the article. We are sorry about it. Though it might not reflect the real costs of training and staff turnover in practice, it could provide some references. We added the following description in the first paragraph under Health economics analysis: "*…alongside the study, including training for the intervention*."

VERSION 2 - REVIEW

**Reviewer**	**2**
**Name**	**Ren, Zhanbing**
**Affiliation**	**Shenzhen University, Physical Education**
**Date**	**23-Jun-2026**
**COI**	

This paper aims to evaluate the clinical effectiveness and cost-effectiveness of a co-produced, tailored physical activity intervention (SPACES) in improving objectively measured MVPA among people with SMI, through a pragmatic randomised controlled trial, thereby providing high-quality evidence to help narrow the health inequality gap between this population and the general public, but the following issues remain:

Page 15 of 136, Lines 8-12: Reducing the statistical power from 90% to 80% due to slow recruitment significantly increases the risk of Type II error, fundamentally undermining the credibility of the study results. It is imperative to clearly justify the clinical or ethical rationale for accepting 80% power, or to extend the recruitment period to maintain 90% power.

Page 20 of 136, Lines 45-52: The definition of compliers in the CACE analysis was not pre-specified before the trial began, leaving room for post-hoc adjustment of analysis criteria based on results, which seriously threatens objectivity. A clear definition of compliers(e.g., ≥75% attendance) must be pre-registered in the Statistical Analysis Plan (SAP) before it is finalised.

Page 4 of 136, Line 24: Failure to quantify or effectively control for assessor bias introduced by unblinded researchers when collecting subjective secondary outcomes (e.g., PHQ-9). Independent blinded assessors should be introduced to reassess a subset of the sample to quantify bias, and secondary outcomes should be treated as exploratory supportive evidence.

Page 9 of 136, Lines 53-57: The title emphasises co-production, but the main text does not provide specific examples of contributions made by patients and the public (PPI) to the intervention design, weakening transparency. At least 2-3 specific, verifiable examples of design contributions made by PPI members should be added to the manuscript.

Page 11 of 136， Line 40-42 ： The description of the accelerometer data processing pipeline (e.g., the algorithm for non-wear time) is insufficiently detailed, compromising the standardisation and reproducibility of this objective primary outcome measure.The algorithm for non-wear time and the criteria for valid days in the GGIR package must be reported in detail to ensure standardised data processing.

Page 24 of 136, Lines 20-26: The sample size calculation was based on mortality benefit, yet the conclusions attempt to link to improvements in psychiatric symptoms; there is a lack of a clear theoretical logical chain between the primary and secondary outcomes. The

introduction should clearly articulate that improvements in MVPA are a precursor to subsequent mental health benefits, and the interpretation of secondary outcomes should be adjusted accordingly.

Page 65 of 136, Lines 25-29: The use of outdated mean imputation for missing baseline covariate data will underestimate data variability and may produce biased statistical results. Mean imputation should be replaced with multiple imputation (MI) or full information maximum likelihood (FIML) to handle missing baseline data.

VERSION 2 - AUTHOR RESPONSE

Response to Reviewer Comments

Reviewers' comments	Responses
Reviewer 2<br>Page 15 of 136, Lines 8-12: Reducing the statistical power from 90% to 80% due to slow recruitment significantly increases the risk of Type II error, fundamentally undermining the credibility of the study results. It is imperative to clearly justify the clinical or ethical rationale for accepting 80% power, or to extend the recruitment period to maintain 90% power.	We agree with the reviewer that reducing the target sample size and statistical power increases the risk of a Type II error.<br>However, we disagree with the reviewer's assertion, that this significantly increases the risk of a Type II error or fundamentally undermines the credibility of the study results.<br>The revised sample size and lower power of the study has been approved by the independent members of the TSC, and DMEC, as well as the funder, sponsor and research ethics committee.<br>If the trial does not recruit to its revised target sample size of 550 then this may undermine the credibility of the study results. We have added the following to the protocol to clarify this (page 14):<br><br>'*The revised sample size and lower power of the study has been approved by the independent members of the TSC, and DMEC, as well as the funder and sponsor. The study protocol has been amended in light of this and this amendment to the protocol has been approved by the West of Scotland NHS Research Ethics Committee who originally approved the study and by the Health Research Authority*.'
Page 20 of 136, Lines 45-52: The definition of compliers in the CACE analysis was not pre-specified before the	We have added the following to page 20 to clarify that the definition of compliers has been prespecified.

trial began, leaving room for post-hoc adjustment of analysis criteria based on results, which seriously threatens objectivity. A clear definition of compliers(e.g., ≥75% attendance) must be pre-registered in the Statistical Analysis Plan (SAP) before it is finalised.	'*The definition of compilers in the CACE analysis has been prespecified in the SAP which has been signed by the PSC*.'
Page 4 of 136, Line 24: Failure to quantify or effectively control for assessor bias introduced by unblinded researchers when collecting subjective secondary outcomes (e.g., PHQ-9). Independent blinded assessors should be introduced to reassess a subset of the sample to quantify bias, and secondary outcomes should be treated as exploratory supportive evidence.	The primary outcome - minutes of MVPA - is collected by accelerometer and is objective and blinded.<br>The protocol (with unblinded assessors) has been approved by the independent members of the TSC, and DMEC, as well as the funder, sponsor and research ethics committee, we are unable to change the protocol and have blinded data collection of the secondary outcomes.
Page 9 of 136, Lines 53-57: The title emphasises co-production, but the main text does not provide specific examples of contributions made by patients and the public (PPI) to the intervention design, weakening transparency. At least 2-3 specific, verifiable examples of design contributions made by PPI members should be added to the manuscript.	We have added additional text as suggested and included an infographic of the intervention development as a supplementary file (page 8).<br><br>'*People with lived experience of SMI, carers and physical and mental health clinicians took part in i. focus groups to identify barriers and enablers and determine the key elements of the intervention, ii. a separate consensus group that advised on each stage of intervention development (see supplementary file 3 for an infographic depicting the intervention development)*.'
Page 11 of 136, Line 40-42：The description of the accelerometer data processing pipeline (e.g., the algorithm for non-wear time) is insufficiently detailed, compromising the standardisation and reproducibility of this objective primary outcome measure. The algorithm for non-wear time and the criteria for valid days in the GGIR package must be reported in detail to ensure standardised data processing.	We have added the following additional information about the accelerometer data processing pipeline (page 10).<br><br>'*A minimum of 16-hour a day wear time for at least 3 days will be accepted as valid wear time over the 10-day collection period. Intensity gradient cut-off points of <30mg, 30-100mg, 101-400mg and 401>mg (inactive, light, moderate and vigorous activity respectively) will be used. Mean MVPA per day is calculated on 5-second epochs with 1-minute bout duration, inclusion criteria of >80%.*'
Page 24 of 136, Lines 20-26: The sample size calculation was based on mortality	The introduction is very much in line with our ethically approved protocol. we

benefit, yet the conclusions attempt to link to improvements in psychiatric symptoms; there is a lack of a clear theoretical logical chain between the primary and secondary outcomes. The introduction should clearly articulate that improvements in MVPA are a precursor to subsequent mental health benefits, and the interpretation of secondary outcomes should be adjusted accordingly.	believe the text in the introduction is appropriate, as it highlights the limited existing research in this area.<br><br>In line with the reviewer's comments, we have removed references to the link between physical activity and mental health symptoms from the discussion.
Page 65 of 136, Lines 25-29: The use of outdated mean imputation for missing baseline covariate data will underestimate data variability and may produce biased statistical results. Mean imputation should be replaced with multiple imputation (MI) or full information maximum likelihood (FIML) to handle missing baseline data.	We agree with the reviewer that mean imputation for the post-randomisation outcome assessments will underestimate data variability and may produce biased statistical results. However, we are expecting very limited missing baseline data.<br>The protocol for the study has been approved by the independent members of the TSC, and DMEC, as well as the funder, sponsor and research ethics committee, who were happy with our proposed analysis of the outcome data.
COI statement(s):<br>If you have selected ‘Yes’ above, please provide details of any competing interests.: Not applicable.	There are no competing interests and have made sure to select ‘no’.

VERSION 3 - REVIEW

**Reviewer**	**2**
**Name**	**Ren, Zhanbing**
**Affiliation**	**Shenzhen University, Physical Education**
**Date**	**03-Aug-2026**
**COI**	

The authors have satisfactorily addressed several previous concerns, particularly those relating to ethical approval, patient and public involvement, lack of blinded follow-up assessment, and the economic evaluation. The trial remains important and potentially publishable. However, several prespecification and cross-document consistency issues require resolution before acceptance.

1、The main manuscript reports Protocol Version 2.0, a target sample size of 550 and 80% power, whereas the supplementary structured summary and SAP retain the previous protocol version, 736 participants, 90% power and an outdated recruitment timeline. Please reconcile the manuscript, SAP, trial registration and all supplementary materials, provide a dated amendment history, and confirm that the sample-size amendment was made without access to comparative outcome data by randomised group.

2、The accelerometer-validity criteria remain inconsistent. The manuscript and main SAP require at least 16 hours of wear per day on at least three days, whereas the SAP appendix requires three weekdays plus one weekend day. Please adopt one definition throughout and prespecify the exact GGIR version, principal processing settings, acceleration thresholds and the meaning of the “>80%” bout criterion.

3、The manuscript states that the CACE adherence definitions have been prespecified but subsequently states that they “will be discussed.” Please report the prespecified definitions directly and remove the contradictory wording. “Compilers” should be corrected to “compliers.”

4、The proposed “while-alive” estimand is not fully aligned with the endpoint and statistical model because MVPA will not normally be measured at death. Please align the estimand, handling of death, missing-data strategy and estimator. One transparent option would be to define the six-month outcome among participants alive at six months and report deaths separately.

5、The study-status statement remains incomplete: “Recruitment to the SPACES study began on .” Please provide the actual recruitment start date and ensure that all recruitment, follow-up and completion dates are consistent across the protocol, registry and supplementary files.

6、The response concerning mean/mode imputation does not address missing baseline covariates. Please identify the affected covariates, justify centre-specific mean/mode imputation, explain how small centres or unavailable centre-specific values will be handled, and prespecify an appropriate sensitivity analysis.

7、Because recruitment is ongoing, “All participants provided written informed consent” should be revised to “All participants will provide written informed consent before participation.”

VERSION 3 - AUTHOR RESPONSE

Reviewers’ comments	Responses
Reviewer: 2<br>Prof. Zhanbing Ren, Shenzhen University, Shenzhen University	We are glad we have satisfactorily addressed several previous concerns and hope we have now fully addressed the pre-specification

The authors have satisfactorily addressed several previous concerns, particularly those relating to ethical approval, patient and public involvement, lack of blinded follow-up assessment, and the economic evaluation. The trial remains important and potentially publishable. However, several prespecification and cross-document consistency issues require resolution before acceptance.	and cross document consistency issues raised.
1、The main manuscript reports Protocol Version 2.0, a target sample size of 550 and 80% power, whereas the supplementary structured summary and SAP retain the previous protocol version, 736 participants, 90% power and an outdated recruitment timeline. Please reconcile the manuscript, SAP, trial registration and all supplementary materials, provide a dated amendment history, and confirm that the sample-size amendment was made without access to comparative outcome data by randomised group.	The manuscript, SAP, and trial registration are now all reconciled. A dated amendment history is included at the front of the SAP.<br><br>We confirm that the sample-size amendment was made without access to comparative outcome data by randomised group.
2、The accelerometer-validity criteria remain inconsistent. The manuscript and main SAP require at least 16 hours of wear per day on at least three days, whereas the SAP appendix requires three weekdays plus one weekend day. Please adopt one definition throughout and prespecify the exact GGIR version, principal processing settings, acceleration thresholds and the meaning of the “>80%” bout criterion.	The accelerometer-validity criteria within the manuscript and SAP are now all reconciled.<br><br>The exact GGIR version (2.9-0) has also been added to the manuscript, together with the versions of R and RStudio being used.<br><br>The principal processing settings, acceleration thresholds and the meaning of the “>80%” bout criterion have also been provided in both the manuscript and SAP.
3、The manuscript states that the CACE adherence definitions have been prespecified but subsequently states that they “will be discussed.” Please report the prespecified definitions directly and remove the contradictory wording. “Compilers” should be corrected to “compliers.”	We have added the two definitions of compliers from the SAP to the paper.<br><br>For participants randomised to the SPACES intervention, we will explore two definitions of compliance, as defined below:<br>• CACE1: Intervention group participants will be considered compliant if they attended at least one session (this can be either an individual session or a group session),<br>• CACE2: Intervention group participants will be considered compliant if they attended at least 50% of both group and individual sessions (at least 9 group sessions and 3 individual sessions).

	Participants randomised to the control group do not have access to the SPACES intervention and will be assumed to be adherent with usual care.
4、The proposed “while-alive” estimand is not fully aligned with the endpoint and statistical model because MVPA will not normally be measured at death. Please align the estimand, handling of death, missing-data strategy and estimator. One transparent option would be to define the six-month outcome among participants alive at six months and report deaths separately.	Handling death events with a treatment policy strategy implies that the endpoint (minutes of MVPA per day at 6-months) is considered regardless of deaths, which is simply impossible and as such would never be a plausible strategy for handling this type of intercurrent event for accelerometer data. The proposed “while alive estimand is fully aligned with the proposed statistical model and implies that we are interested in the effect on outcome until death, and that we will include participants’ outcome data in the analysis until they die, and not include (or impute) any outcome data thereafter.
5、The study-status statement remains incomplete: “Recruitment to the SPACES study began on .” Please provide the actual recruitment start date and ensure that all recruitment, follow-up and completion dates are consistent across the protocol, registry and supplementary files.	The manuscript has been updated to say ‘Recruitment to the SPACES study began on the 28th October 2024’.
6、The response concerning mean/mode imputation does not address missing baseline covariates. Please identify the affected covariates, justify centre-specific mean/mode imputation, explain how small centres or unavailable centre-specific values will be handled, and prespecify an appropriate sensitivity analysis.	We have clarified that subjects will be included in the primary analysis model provided they have at baseline and least one post-randomisation measurement of MVPA.<br><br>The revised SAP outlines how we will deal with missing baseline and primary outcome data as part of a series of sensitivity analyses. We do not expect any missing data for randomised group and recruiting centre.<br><br>The SAP now says<br><br>5.6.1 Missing baseline accelerometery and MVPA outcome data<br>Subjects will be included in the primary analysis model provided they have at baseline and least one post-randomisation measurement of MVPA. As part of a sensitivity of the primary outcome, missing baseline MVPA outcome data will be handled by mean imputation, which will be done by the recruiting centre to ensure that all participants with any valid post-randomisation outcome data are included in the analyses [41]. In the event of only one

	participant per recruiting centre and that participant has missing baseline MVPA outcome data we will use the baseline mean minutes of MVPA per day for the randomised group. Summary statistics of baseline data will be restricted to the observed data only. As described above, some analysis models will be adjusted for baseline data within which missing data have been mean-imputed. This approach will be clarified in all relevant tables and other study outputs.
7、Because recruitment is ongoing, “All participants provided written informed consent” should be revised to “All participants will provide written informed consent before participation.”	Amended to say “All participants will provide written informed consent before participation.”
COI statement(s):<br><br>Reviewer: 2<br>If you have selected ‘Yes’ above, please provide details of any competing interests.: Not applicable.	‘No’ has been selected and the wording ‘not applicable’ used previously will not be used in the field below this question box as I fear it may be causing the issue raised here.
