# Supplementary material for: Supporting physical activity through co-production in people with severe mental illness (SPACES): study protocol for a pragmatic, parallel-group randomised controlled trial
Source: BMJ Open. 2026 Oct 8;16(10):e115528. doi: 10.1136/bmjopen-2025-115528 (PMC13656100; doi:10.1136/bmjopen-2025-115528)
Supplement: Supplementary file 3 [file bmjopen-16-10-s003.docx]

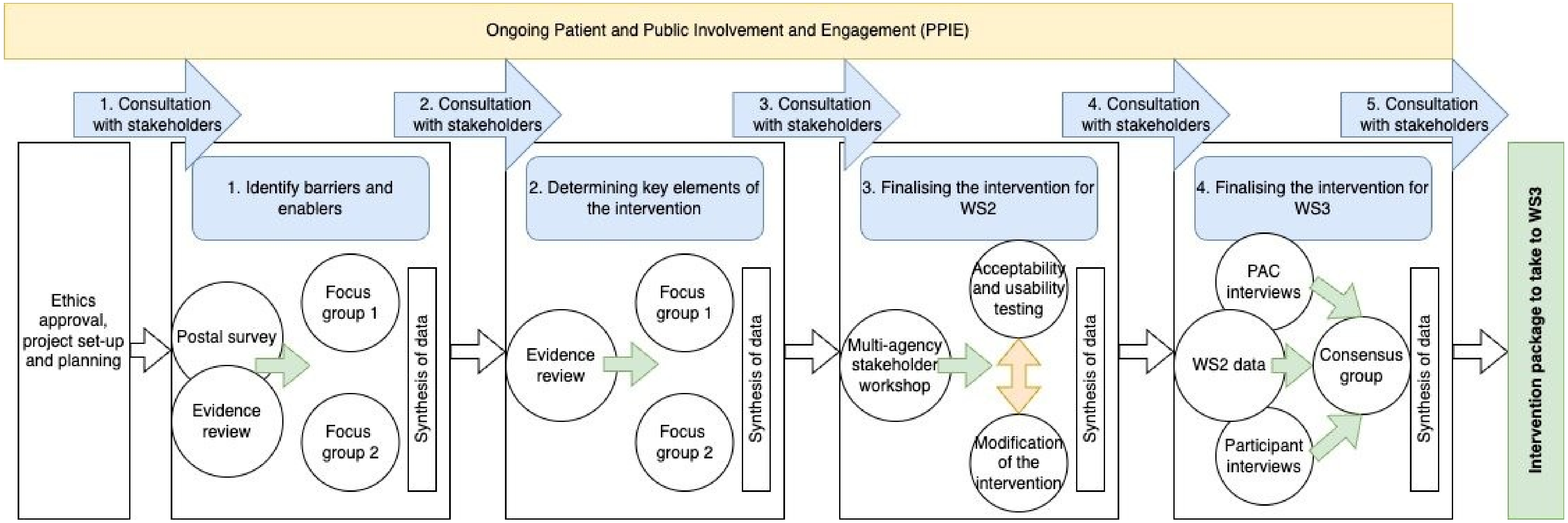

Ongoing Patient and Public Involvement and Engagement (PPIE)
1. Consultation with stakeholders
2. Consultation with stakeholders
3. Consultation with stakeholders
4. Consultation with stakeholders
5. Consultation with stakeholders
Ethics approval, project set-up and planning
1. Identify barriers and enablers
Postal survey
Evidence review
Focus group 1
Focus group 2
Synthesis of data
2. Determining key elements of the intervention
Evidence review
Focus group 1
Focus group 2
Synthesis of data
3. Finalising the intervention for WS2
Multi-agency stakeholder workshop
Acceptability and usability testing
Modification of the intervention
Synthesis of data
4. Finalising the intervention for WS3
PAC interviews
WS2 data
Participant interviews
Consensus group
Synthesis of data
Intervention package to take to WS3
