# Supplementary material for: Supporting physical activity through co-production in people with severe mental illness (SPACES): study protocol for a pragmatic, parallel-group randomised controlled trial
Source: BMJ Open. 2026 Oct 8;16(10):e115528. doi: 10.1136/bmjopen-2025-115528 (PMC13656100; doi:10.1136/bmjopen-2025-115528)
Supplement: Supplementary file 3 [file bmjopen-16-10-s001.docx]

**Structured summary of the SPACES RCT**

| **Item** | **Description** |
| --- | --- |
| Primary Registry and Trial Identifying Number | ISRCTN23019026 <http://doi.org/10.1186/ISRCTN23019026>; registered on 25 July 2024. |
| Secondary Identifying Numbers | None |
| Source(s) of Monetary or Material Support | National Institute for Health Research Programme for Applied Research (NIHR PGfAR, RP-PG-102618)  Contact: Sarah Thompson  Email: sarah.thompson@nihr.ac.uk |
| Primary Sponsor and contact information | Sheffield Health Partnership University NHS Foundation Trust  Wendy Swann, Research Manager  Sheffield Health Partnership University NHS Foundation Trust, Centre Court, Atlas Way, Sheffield, S4, 7QQ  Telephone: 0114 2716605  Email: [rdu@sheffieldpartnership.nhs.uk](mailto:rdu@sheffieldpartnership.nhs.uk) |
| Role of sponsor and funder | The sponsor played no part in study design and will play no part in the collection, management, analysis, interpretation of data, the writing of the report, or the decision to submit the report for publication. |
| Contact for Public Queries | Matthew Faires  [matthew.faires@yorksj.ac.uk](mailto:matthew.faires@yorksj.ac.uk)  <https://www.spacesproject.co.uk/> |
| Contact for Scientific Queries | Dr Emily Peckham  [e.peckham@bangor.ac.uk](mailto:e.peckham@bangor.ac.uk) |
| Public Title | SPACES: Supporting Physical Activity through Co-production in people with Severe Mental Illness |
| Scientific title | Supporting Physical Activity through Co-production in people with Severe Mental Illness (SPACES): a pragmatic parallel group randomised controlled trial |
| Countries of Recruitment | UK |
| Health Condition(s) or Problem(s) Studied | Physical activity behaviour among individuals living with severe mental illness such as schizophrenia, bipolar disorder, and schizoaffective disorder. |
| Intervention(s) | Control and intervention groups: Both groups will receive usual care.    Intervention group only: In addition, the intervention group will receive the offer of an 18-week group-based intervention comprising walking, indoor activity, designated social time, community taster sessions, and up to 6 one-to-one sessions, designed to increase physical activity and reduce sedentary behaviour |
| Key Inclusion and Exclusion Criteria | Inclusion criteria   1. Age: 18 years or over. 2. ICD-10 or DSM-V diagnosis of SMI as documented in GP or psychiatric notes (schizophrenia (F20 and subcodes); schizoaffective disorder (F25 and subcodes); other psychotic and delusional disorders (not of organic origin) (F21-F24 and F26-F29 and subcodes); bipolar and manic episodes (F30 and F31 and subcodes)). 3. Are able to walk unaided. 4. Are willing to wear an accelerometer.   Exclusion criteria   1. People who lack capacity to participate in the trial as guided by the Mental Capacity Act (2005) 2. Primary diagnosis of drug or alcohol abuse 3. Medical contraindication of physical activity as ascertained by GP or mental health team. 4. Already physically active (e.g., >300 minutes/week of self-reported MVPA) 5. Non-English speakers 6. People who took part in the feasibility study |
| Study Type | A two-arm, multi-site pragmatic parallel group, randomised controlled superiority trial. Due to the nature of the interventions in this study, blinding of participants, clinicians, and researchers is not feasible. The trial statistician will be blind to group allocation until the statistical analysis plan is signed off. The senior statistician will be unblinded to be able to contribute to Data Monitoring and Ethics Committee meetings. |
| Current protocol details | Version 2.0 dated 8^th^ May 2026 |
| Date of First Enrollment (planned) | Patient recruitment began 28 October 2024 and was due to be completed on 30 June 2026. Approval was granted on 8 May 2026 by the West of Scotland Research Ethics Service for the end date of the trial period to be extended to 31 March 2028, and for the recruitment end date to be extended to 28 February 2027. |
| Sample Size | Initially 736 participants (368 per arm) were to be randomised 1:1 into the intervention or usual care arms, providing 90% power. Approval was granted on 8 May 2026 for a revised target sample size of 550 participants (275 pr arm) providing 80% power. The authors confirm that the sample-size amendment was made without access to comparative outcome data by randomised group. |
| Primary outcome(s) | Objective accelerometer-derived minutes per day of Moderate to Vigorous Physical Activity (MVPA) at 6 months post-randomisation. |
| Key Secondary outcome(s) | Secondary outcomes are include light physical activity, sedentary behaviour, self-reported physical activity and sedentary behaviour, smoking behaviour, body mass index, depression, anxiety, loneliness, health-related quality of life, healthcare resource use, predictive behaviour and sedentary behaviour, body mass index, depression, anxiety, loneliness, health-related quality of life, healthcare use, and adverse events. Measures will be taken at baseline and at three-, six-, and twelve-months post-randomisation. |
| Ethics Review | Ethical approval provided by the West of Scotland NHS Research Ethics Service (WoSRES) and the HRA on the 14 May 2024; ref: 24/WS0040. Further ethical approval provided by WoSRES and HRA on the 8 May 2026 to revise the end date of the trial period to 31 March 2028, recruitment end date to 28 February 2027 and revised the target sample size downwards to 550. Any protocol amendments will be signed off by the REC and HRA and will be reported to PGfAR in progress reports. |
| Individual Trial Participant Data sharing statement | This document constitutes a structured summary of the full protocol. Following completion of the RCT, datasets and statistical code used in this RCT will be available from the Clinical Trials Unit at the Sheffield University ([ctru@sheffield.ac.uk](mailto:ctru@sheffield.ac.uk)) on reasonable request. |
