# Supplementary material for: Supporting physical activity through co-production in people with severe mental illness (SPACES): study protocol for a pragmatic, parallel-group randomised controlled trial
Source: BMJ Open. 2026 Oct 8;16(10):e115528. doi: 10.1136/bmjopen-2025-115528 (PMC13656100; doi:10.1136/bmjopen-2025-115528)
Supplement: Supplementary file 4 [file bmjopen-16-10-s002.docx]

**S**upporting **P**hysical **A**ctivity through **C**o-production in p**e**ople with **S**evere Mental Illness (SPACES)

**Consent Form**

**Part 1 (Main Consent)**

|  | **Participant Initials** | **Researcher Initials** |
| --- | --- | --- |
| 1. I have read and understand the information sheet (enter version and date), have discussed the study with a SPACES team member and have had the chance to ask any questions and I understand that the research involves possible participation in a physical activity programme. 2. I understand that I will receive a physical activity programme or usual care (my normal care from the NHS) and that this will be selected at random and not by the SPACES team. 3. I understand that I will need to spend up to 60 minutes on completing forms and questionnaires 4 times over 12 months and that I might need to take part in a physical activity programme. 4. I agree that researchers may use my anonymised data in publications, reports, web pages, and other research outputs. 5. I understand that other researchers outside of the SPACES team can have access to my anonymised data for research purposes only. 6. I understand that the SPACES team will share information about my participation with my GP/CMHT care team. 7. I understand that my participation is voluntary and that I am free to withdraw at any time without giving any reason, and without my medical care or legal rights being affected. I understand that I need to let the study team know if I wish to withdraw from the study. 8. I agree to The University of Sheffield holding my contact details in order to contact me about the study. I understand that my contact details will not be shared with anyone outside the research team. 9. I give my consent to taking part in this SPACES study given all the information I have received so far. |  |  |

**Part 2 (Additional items)**

Please be aware, you will still be able to consent to taking part in the SPACES study even if you answer ‘No’ to the following items:

|  | **Yes** | **No** | **Participant Initials** | **Researcher Initials** |
| --- | --- | --- | --- | --- |
| 1. I am happy for the research team to contact me about taking part in an interview to talk about my experiences of being part of the study. |  |  |  |  |
| 2. I am happy to be contacted by the research team with the study findings once the research is complete. |  |  |  |  |

**Contact Details (please complete in BLOCK CAPITALS)**

First name: ________________________________________________________________________

Surname: __________________________________________________________________________

Address: __________________________________________________________________________

___________________________________________________________________________

___________________________________________________________________________

Postcode: _________________________________________________________________________

Phone number:	_____________________________________________________________________

Email address: ______________________________________________________________________

**Signature (participant):**

**(Please sign here)**

**Date:** DD	/	MM	/	YYYY

**Signature (researcher):**

**(Please sign here)**

**Date:** DD	/	MM	/	YYYY

Thank you for completing the consent form. If you have any queries or would like any further information about this study, please contact:

**How to get in touch with us**

*<Insert contact details>*

Office Use

Participant ID: _________ Consent Taken By: _______________________________________

Date: ________________

Copy of consent form (tick):

☐ Research File	☐ Participant	☐ GP
